# From gut to brain: effects of fecal microbiota transplants from humans to rats on hippocampal gene regulation - a study on anorexia nervosa

**DOI:** 10.1038/s41398-026-04056-9

**Published:** 2026-04-30

**Authors:** N. M. Korten, L. Blischke, A. C. Thelen, A. Schulze Eckel, M. van Egmond, V. Verspohl, M. Neumann, L. Kneisel, M. Tran, C. Beyer, B. Herpertz-Dahlmann, L. Keller, C. Bang, N. A. Andreani, J. Seitz, S. Trinh, C. Voelz

**Affiliations:** 1 Institute of Neuroanatomy, Uniklinik RWTH Aachen, Wendlingweg 2, 52074 Aachen, Germany; 2 Department of Child and Adolescent Psychiatry, Psychosomatics and Psychotherapy, Uniklinik RWTH Aachen, Pauwelsstraße 30, 52064 Aachen, Germany; 3 Department of Child and Adolescent Psychiatry, Psychosomatics and Psychotherapy, LVR University Hospital Essen, University Duisburg-Essen, Wickenburgstr. 21, 45147 Essen, Germany; 4 Institute of Clinical Molecular Biology, Christian Albrechts University of Kiel and University of Schleswig-Holstein, Campus Kiel, Rosalind-Franklin-Str. 12, 24105 Kiel, Germany; 5 Institute for Experimental Medicine, Christian-Albrechts-University of Kiel, 24105 Kiel, Germany; 6 Department of Biology and Biotechnology Charles Darwin, Via dei Sardi, 70, Rome, 00185 Italy; 7 Hannover Medical School (MHH), Institute of Functional and Applied Anatomy, Carl-Neuberg-Str. 1, 30625 Hannover, Germany; 8 nextGENERATION Medical Scientist Program, Dean’s Office for Academic Career Development, Hannover Medical School, Hannover, Germany

**Keywords:** Molecular neuroscience, Psychiatric disorders

## Abstract

Fecal microbiota transplantation (FMT) has emerged as a novel approach for understanding anorexia nervosa (AN), a complex eating disorder characterized by severe underweight, fear of weight gain and distorted body image. Patients with AN show alterations in the gut microbiome, brain structure, and inflammatory processes, indicating the importance of the microbiome‒gut‒brain axis in AN pathology. This study aimed to investigate whether FMT from patients with AN into antibiotic-treated rats could transfer a phenotype associated with the disease inducing AN-like symptoms and hippocampal alterations. Female Wistar rats received antibiotics followed by FMT from healthy controls, patients with AN, or water. Gut microbiota effects were assessed through 16S rRNA gene sequencing, alongside post-mortem analyses of glial cells, neurogenesis markers, and inflammatory markers. The results revealed dysregulated microbial diversity after antibiotic treatment, which was partially restored after FMT. Successful transfer of human bacterial species was observed, but AN-like symptoms and changes in glial/neuronal counts were not detected. Notably, a decrease in hippocampal *Bdnf* expression was detected in the antibiotic control group, which was reversed by healthy control stool transplantation but not in the AN-transplanted group. Similar patterns were observed for neuroinflammation and *Mki67*, a marker of cell neogenesis. These findings suggest potential links between microbial changes, neuroinflammation and neuroplasticity in the hippocampus with the potential to correct deficits with FMT. Future studies should extend these findings by exploring the combination of FMT and starvation phases to better understand the roles of specific microbial populations in neuroinflammatory processes and, ultimately, clinical outcomes in AN.

## Introduction

Anorexia nervosa (AN) is one of the most common (often) chronic mental disorders in adolescents [1] characterized by low caloric intake and strong urge to exercise, leading to extensive weight loss. Patients with AN typically suffer from body image distortion and weight phobia [2]. Furthermore, cognitive deficits, defined by memory loss, impaired decision-making skills, and a loss of cognitive flexibility, are often found [3–5]. Current treatment options, including a combination of weight recovery, psychotherapy, and for adolescents, family interventions, have shown limited treatment success [6]. Furthermore, pharmacotherapy continues to concentrate on the treatment of psychiatric comorbidities as depression or anxiety disorder rather than exerting an influence on AN directly [6, 7]. Unfortunately, relapse rates and the risk of disease chronification are still high [1]. Considering the multifactorial pathogenesis of AN, microbial shifts, hormonal and endocrine pathways, genetics, epigenetic profiles, and gut–brain organ-crosstalk are considered key players [8–10]. Therefore, in recent years the classification of AN as a metabo-psychiatric disorder with metabolic pathways being closely interlinked with psychiatric symptoms has been debated [11–13]. In this context alterations of the gut microbiome in patients with AN have been studied more extensively [14]. The human microbiome is a complex community of trillions of microorganisms that play crucial roles in maintaining health conditions [15]. Gut microbial composition analyses of patients with AN have revealed heterogeneous alterations in alpha- (measuring species evenness) and beta-diversity (measuring species composition) compared with healthy controls [14, 16]. Starvation-induced dysbiosis show that some gut bacteria might influence AN development and further enhance its progression [17–19]. Precisely, a decreased abundance of butyrate-producing bacteria like *Bacteroidetes* and *Roseburia* and augmented levels of mucin-degrading species such as *Firmicutes* are described in AN. As a result, mucin reduction and the loss of protective acids through dysbiosis may lead to elevated intestinal permeability and inflammation [20]. Thereby, the shift of bacterial taxa can contribute to organ crosstalk and influence the progression and facilitation of AN and interestingly, both, the appetite-stimulating hormone ghrelin and the satiety hormone leptin are linked to an altered microbial composition [21]. Performing a longitudinal study with more than 50 adolescent patients with AN, Andreani et al. identified an association between the abundance of certain bacterial taxa in patients’ feces at hospital admission and these patients´ re-admission after one year (calculated via SDS-BMI) [18].

The extent to which the microbiome influences cognitive impairment via the gut-brain axis still needs to be decoded. However, previous studies already connected the gut-microbiome to psychiatric disorders [22–25]. In AN, cognitive flexibility is impaired. This impairment could be associated with brain volume loss affecting both gray and white matter including the hippocampus [26, 27], where volume reduction is associated with memory loss in AN [5, 28]. Interestingly, with weight recovery, most differences appear reversible [29]. In terms of cognitive impairment, the hippocampal dentate gyrus has emerged as a unique player in remodeling processes in the mature brain, with potential roles in proliferation and regeneration [30–32]. It plays central functional roles in memory, learning, and decision-making—processes that are impaired in the acute phase of AN. Previous studies detected reduced hippocampal volumes in not only patients with AN but also animal models, highlighting the hippocampus as an interesting target in AN research [5, 28, 33]. To study AN in animals, different models have been established, of which the activity-based anorexia (ABA) is the most common. It combines starvation and increased physical activity [34–36]. Animal models have successfully demonstrated and validated the findings observed in human studies: Trinh et al. reported that starvation itself rather than increased physical activity causes microbial dysbiosis in an ABA animal model in rats [37]. Additionally, with this model, brain volume and cell reductions could be detected, similar to the pseudoatrophy observed in human patients. Also, inflammatory processes increased in the hippocampal area, which might impact its function [38]. Brain matter consists of several different cell types especially neurons and glia cells. In AN, (reversible) glia cell reduction was observed, that could play a role in the development of cognitive impairment in patients with acute AN [39, 40]. Glial cells play essential roles in supporting neuronal function, including maintaining homeostasis, forming myelin, and modulating immune responses in the central nervous system [41]. In addition to their supporting function, astrocytes, a type of glial cell, are important for the transport and uptake of neurotransmitters and metabolic products, as well as maintaining the blood‒brain barrier [42]. The reduction in astrocytes could therefore offer an approach to explain the neuropsychological deficits found in patients with AN [39]. Furthermore, microglia are resident macrophages and thus constitute part of the immune defense within the central nervous system [43]. In AN, the activation of microglia, characterized by increased inflammatory markers, has been detected in previous animal studies and could additionally influence neuropsychological deficits [44, 45]. With respect to white matter alterations, changes in the oligodendrocyte cell count following the ABA protocol were demonstrated by our laboratory [38, 46]. Regarding psychiatric disorders, a reduction of oligodendrocytes and changes in myelination reflect behavioral changes as seen in disorders like schizophrenia [47]. Also, myelination by oligodendrocytes has been shown to be dynamically regulated by microbiota changes in the prefrontal cortex, potentially also impacting AN pathology [48].

In recent years, upcoming studies on AN have increasingly focused on this microbiome-gut-brain-axis in order to gain a deeper understanding of the complex pathogenetic interaction in the development and maintenance of AN. As a novel approach for studying the impact of microbes on (mental) disease progression, fecal microbiota transplantation (FMT) has attracted scientific interest. The transfer of bacterial feces from specific (healthy) donors into a new host organism is performed to induce a donor-like phenotype via the microbiome‒gut‒brain axis. Other metabo-psychiatric diseases such as depression and obesity have been targeted for FMT treatment, with promising results [49, 50]. Concerning AN, seven animal studies as well as two human case reports have been published presenting heterogenous results [51–59]: Only three out of seven animal studies reported phenotypic changes resulting from fecal transplants [51, 52, 58], while the remaining four studies showed (at least) partial alterations in the microbial composition [53–55, 59]. Regarding the human case reports, one of the two patients showed an improvement in AN symptoms after transplantation [56, 57]. Thus, even though several groups have performed FMT from patients with AN into rodents, the great heterogeneity in not only the studies´ protocols but also their results highlight the need of further research to firstly gain a deeper understanding of the crucial key players for an efficient bacterial transfer and, secondly, the induction of an AN-like phenotype [60].

The present study aims to gain a deeper understanding of the regulatory role of the microbiome‒gut‒brain axis during the development and persistence of AN. Therefore, the primary objective of this study was to ascertain whether FMT from patients with AN can be successfully transferred into rats using our protocol. Subsequently, it was assessed whether transplantation would induce an AN-like phenotype. The secondary objective of this study was to investigate the influence of the gut microbiome on cell composition in the hippocampus including glial cell expression and altered cell neogenesis. Additionally, to identify the influence of the AN-derived microbiota on the dysregulation of inflammatory parameters, different markers of inflammation were exhibited. To our knowledge, this is the first study to focus on changes in the hippocampus after FMT from patients with AN particularly. With a well-established, good functioning FMT protocol, influences on the whole metabolism including the gut-brain axis can be studied in more detail leading to promising new insights in AN research.

## Materials and methods

### Donors: patients and recruitment

Four stool sample donors were selected from patients recruited as part of the *MiGBAN* study at the Department of Child and Adolescent Psychiatry, Uniklinik RWTH Aachen [61]. All patients were female, diagnosed with AN according to DSM-5 [62], and aged between 12 and 19 years. Selection was based on symptom severity, as indicated by a low body mass index at admission (see Table 1). Additionally, five age- and sex-matched control donors with normal body weights were recruited. Ethical approval for the *MiGBAN* study was obtained from the respective ethics committee (approval no. EK 194-19).

**Table 1:** Information on human fecal donors.

Donor	Age admission	BMI admission	BMI-SDS admission (KiGGS data)	Illness duration (weeks)	Diet	EDE-Q total score	EDI-2 total score
P01	13.82	11.64	−5.33	12.14	omnivorous	0.39	160
P02	15.32	14.15	−3.70	21.14	omnivorous	4.68	242
P03	14.22	16.04	−1.83	16.00	vegetarian	5.05	373
P04	16.16	13.75	−4.72	16.86	pescetarian	1.33	192
*mean Ps*	14.88	13.90**	−3.9	16.54		2.86	241.75
HC01	15.29	20.24	−0.31		omnivorous	0.21	219
HC02	15.79	26.14	1.17		omnivorous	0.67	227
HC03	13.78	17.69	−0.98		pescetarian	0.11	145
HC04	13.36	20.04	0.03		vegetarian	0.37	164
HC05	14.81	19.38	−0.54		omnivorous	0.76	241
*mean HCs*	14.61	20.70	−0.13			0.42	199.2

*P* patients with anorexia nervosa, *HC* healthy controls.

*BMI* body mass index.

*BMI-SDS* BMI-Standard-Deviation (KiGGS data: nationally reference sample).

*EDE-Q score* eating disorder examination questionnaire.

*EDI-2 score* eating disorder inventory 2.

*p* < *0.01* = **, in comparison to control group.

### Fecal sample collection, purification, and application

Fecal material was collected in a container with anaerobic sachets (Oxoid^TM^ AnaeroGen^TM^ Compact, Thermo Fisher Scientific, MA, USA). After dilution (1:10) in pre-reduced phosphate-buffered saline (PBS) glycerol buffer and homogenization, the material was centrifuged to remove debris (5 min, 500 rpm), filtered through a 70 µm sterile filter and stored at −80 °C.

### Recipients: animals

Female Wistar rats, aged four weeks, were used for the experiments (RjHAN:WI, Janvier, France). The study adhered to the FELASA guidelines and complied with DIN ISO 9001:2015 certification. The animals were housed individually in cages equipped with a built-in running wheel and tachometer (Sigma BC 5.12). The environmental conditions were maintained at a stable temperature of 22 °C, 55% humidity, and a 12h light/dark cycle (lights on at 7 am). The animal protocol received approval from the Governmental Animal Care and Use Committee of North Rhine-Westphalia (LAVE). All procedures conformed to German animal experimentation laws [63] and Directive 2010/63/EU on the protection of animals used in scientific research (Official Journal of the European Union, 2010).

### Study design

**Fig. 1:**
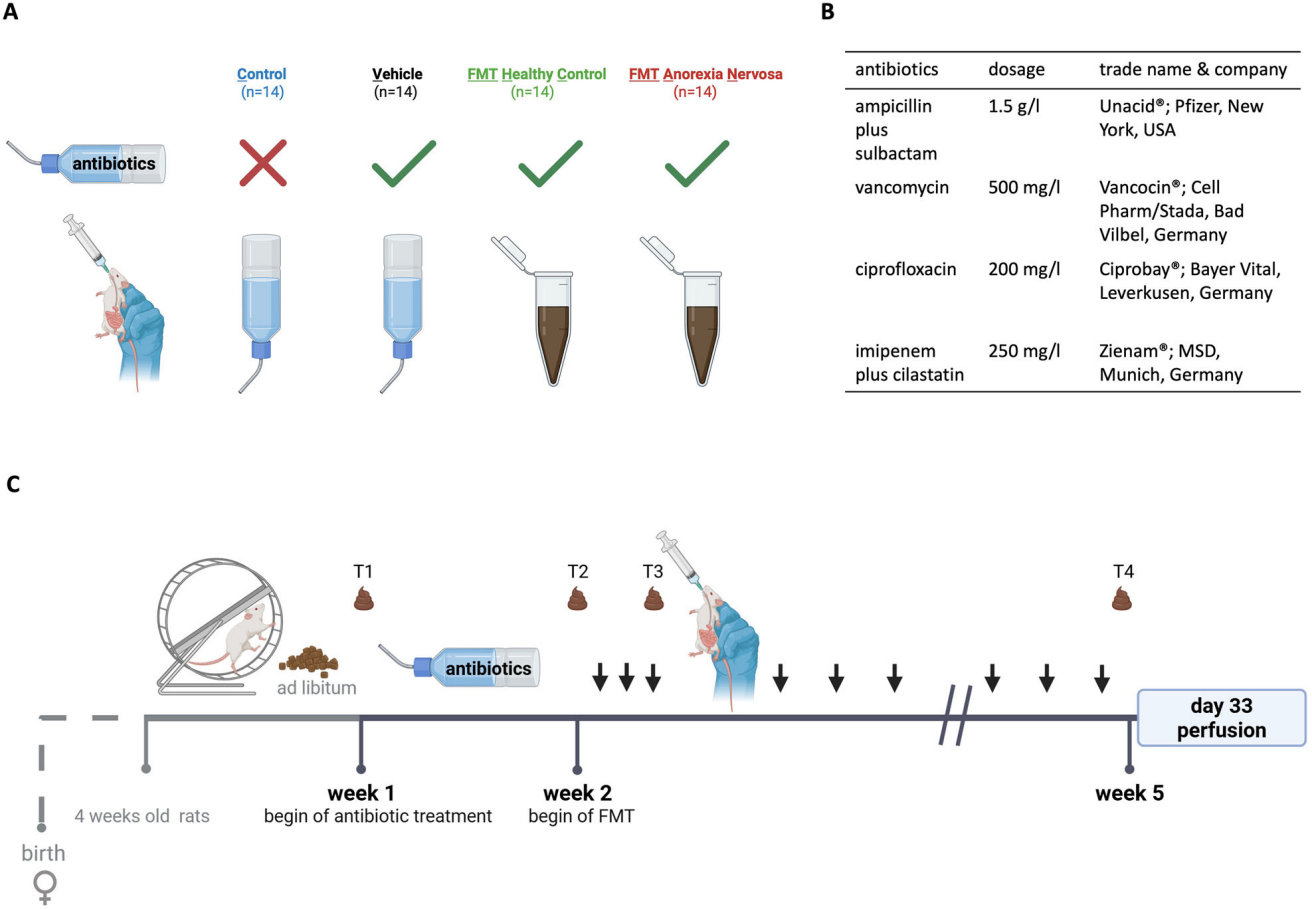
Illustration of the study design. **A** Four groups with different treatments. All groups except controls received antibiotics prior to fecal transplants. The control and vehicle groups were given water via oral gavage, the healthy control-transplanted and anorexia nervosa-transplanted groups received fecal microbiota transplants as illustrated in the study timeline. **B** List of antibiotics-mixture administered to the rats (week 1 = days 1–7). **C** Timeline of the five-week period of the experiment. Stool samples were taken at four distinct time points, namely at baseline (day 1 = T1), after ABX treatment (day 7 = T2), after the first three administrations of FMT (FMT 1–3, day 9 = T3), and at finalization (day 33 = T4). Administrations of fecal microbiota transplants are marked with an arrow.

After a one-week acclimatization period (referred to as days −7 till 0), four groups of 14 rats each were established (Fig. 1A). The animals were randomly placed in cages with predefined group assignments. During the experiment, the researcher knew which group each animal belonged to. The animals’ food and water intake, running wheel activity (RWA), body weight (BW), and general well-being were monitored daily at noon. For seven days, all the groups except the controls received an antibiotic (ABX) mixture via the drinking water (see Fig. 1B). On day eight, FMT (0.3 mL) was initially given for three consecutive days via oral gavage. Afterward, FMT was given every third day until the end of the experiment (nine times in total). The control and vehicle groups received the same amount of water via oral gavage. The transplant groups received feces from either healthy controls (“FMT HC group”) or patients diagnosed with AN (“FMT AN group”) (Fig. 1C).

Each sample from one human donor was transplanted into three to four different animals. Thus, donor-specific effects could be traced due to the high individuality of the gut microbiome. Also, bacteria-specific differences in transfer efficiency were assumed. By transplanting different donor stools we aimed to identify *superdonors* who present with a microbial composition that can be transplanted and reliably induces an AN-like phenotype in the recipients.

All the animals had unrestricted access to food, water, and the running wheel throughout the experiment. Fresh fecal pellets were collected at four distinct time points and stored at −80 °C: baseline (T1, day 1), post ABX (T2, day 7), post 3x FMT (T3, day 9), and finalization (T4, day 33). To evaluate the impact of FMT on brain composition, the hippocampus (Hi) was harvested for fixation and RNA isolation.

### Microbial analysis

For detailed information on the processing of the collected fecal material, DNA extraction and 16S rRNA gene sequencing, see Supplementary File 1 or [64]. Briefly, after DNA isolation the V3/V4 variable regions of the 16S rRNA gene were amplified and sequenced. Exhibited amplicon sequence variants (ASV) were then used for statistic calculations. Based on previous studies several crucial parameters of the microbial composition were analyzed in detail starting with the global microbial composition (relative taxa abundance). To ensure microbial depletion after treatment with antibiotics, changes in the fecal biomass at T2 (post ABX) were measured. Furthermore, alterations in alpha diversity (Chao1 index and Shannon index) and beta diversity (Bray-Curtis dissimilarity index) over time were examinated depicturing the species diversity within a single individual (alpha diversity) as well as differences in microbial composition between individuals (beta diversity) [14, 16]. Statistical tests performed for each microbial parameter are described in the statistic section. Overlapping bacterial taxa between specific donors and the according animals at T1 (baseline) and T4 (finalization), respectively, were identified by selecting combined taxa manually.

### Sample collection and preparation

The rats were euthanized with 100% isoflurane (Piramal, India) and perfused transcardially with 150 mL of PBS (Thermo Fisher Scientific, MA, USA). The brains were removed on ice and divided midsagittally. The right hemisphere was fixed in a 3.7% paraformaldehyde (Roth, Karlsruhe, Germany) solution (pH 7.4) for paraffin embedding. The hippocampus of the left hemisphere was dissected and prepared on a Petri dish on ice before being shock-frozen in liquid nitrogen and stored at −80 °C for RNA isolation. Human samples were processed as described previously [18] as part of the *MiGBAN* study.

### Immunohistochemistry

Five-micron sections were sliced at Bregma −2.30 using a microtome (RM2255, Leica, Germany). Two slices per animal were stained as previously described [65]. A negative control was included in each staining process. Markers for the three above mentioned glial cell types (astrocytes, microglia, and oligodendrocytes) as well as neuronal markers were used for stainings. The applied antibodies are listed in the Supplementary Table 1. Images of the stained tissue were digitalized using a Nikon Eclipse 80i microscope (Nikon, Leuven, Belgium). The staining was examined in the dentate gyrus of the hippocampal formation since it holds special function regarding plasticity of the adolescent brain as mentioned earlier and further forms an anatomical landmark. Therefore, two sections at the same Bregma (−2.52) for each animal sample were included. The digital images were randomized using VirtualBlind software. Two observers counted the number of positive cells using ImageJ software (version 1.52a; NIH, Bethesda, USA) unaware of which group the animal´s sample belonged to. Cell counts of each sample were normed to the image section size to minimize variability. A representative image of one animal per group for each staining can be found in Supplementary File 2.

### RNA isolation, reverse transcription, and semiquantitative real-time PCR (RT‒qPCR)

RNA isolation was performed using phenol‒chloroform (RNAsolve, VWR, USA) as previously described [65]. Afterward, the mRNA was retrotranscribed into complementary DNA (M-MLV RT kit and random primers, Life Technologies Corporation, California, USA). Successful transcription was confirmed via PCR and agarose gel electrophoresis (see reference genes in the Supplementary Table 2: cyclophilin A and beta-2-microglobulin). RT‒qPCR was performed as previously described [65]. Thus, gene expressions of glial cells (*Gfap, Olig1, Aif1*), neuroproliferatory markers (*Mki67, Bdnf, Map2, Rbfox3, Dcx*) and inflammatory markers (*Cd11b, Il6, Tnf*) were assessed. Detailed information on the primers used for RT‒qPCR is listed in the Supplementary Table 2. Normalization was ensured by determining the ratio between the target gene and the reference genes (cyclophilin A and beta-2-microglobulin). Changes in the expression of target genes are displayed as fold changes relative to controls (ΔΔCt).

### Statistical analysis

The adequate sample size for this experiment was pre-calculated with a power analysis in the animal proposal. Body weight was chosen as outcome parameter. With a linear mixed effects model, and a power of 80% the statistical basis for the group size was calculated and subsequently applied for and carried out accordingly. All the collected data were subjected to statistical analysis using SPSS software (version 29; IBM, USA), with a significance level of 5% (p-values that did not meet statistical significance are not included). Visualization was performed using GraphPad Prism (10.1.2, Boston, USA). The handling data were normalized to the individual values of the animals on the last day of habituation and are presented as the means per day and standard error of the mean (SEM). One-way ANOVA with a Bonferroni post hoc correction was conducted. The Grubbs test was used for outlier testing, which led to exclusion of a few samples. Therefore, the final total number of animals included per group in each analysis is given in Supplementary Table 3 as n(analysis)/n(total). Figures were created with *bioRender*.

The sequencing data were analyzed using the R package on the *MicrobiomeAnalyst* platform [66–68]. The microbial composition was studied at the *genus* level, with the rarefaction cutoff set to the lowest value of our samples (9766 reads). For the relative taxa abundance (Wilcoxon test) and exploratory Spearman correlation analyses, only the core microbiota was considered, with a cutoff sample prevalence of 20% and a minimal relative abundance of 1%. Alpha diversity was measured by the Chao1 and Shannon indices and a repeated measurement ANOVA. Beta diversity was calculated based on the Bray‒Curtis dissimilarity index. For pairwise comparisons, PERMANOVA tests were performed.

## Results

### No effect of FMT on phenotypic development of recipient rats

**Fig. 2:**
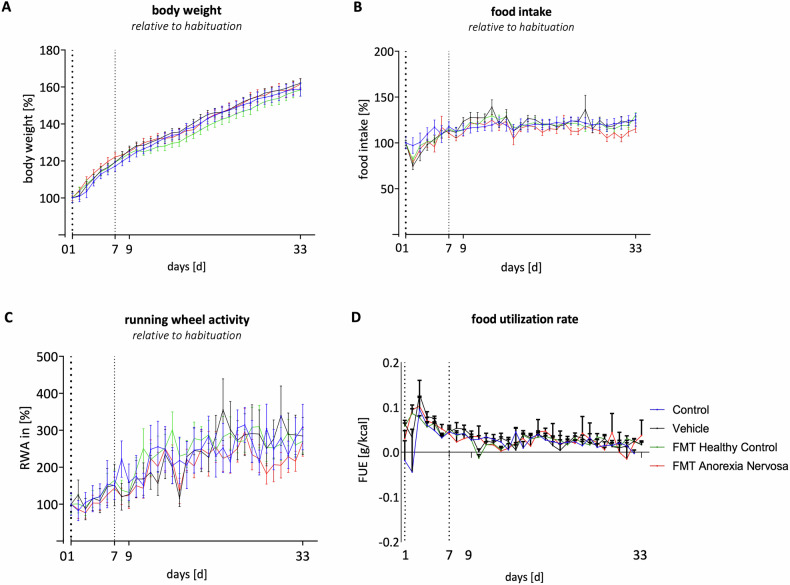
Illustration of physiological data. **A–C** Development of body weight (**A**), food intake (**B**), and running wheel activity (**C**) relative to habituation. First dotted line marks the end of habituation phase, second dotted line marks the beginning of FMT treatment. Repeated measurement ANOVA performed for different time periods: the ABX treatment (days 1–7), the total FMT interval (days 8–33), and the overall experimental duration (days 1–33). **D** Development of food utilization efficiency (FUE) based on change in body weight per eaten calories per day (g/(kcal/d)). Illustrated as mean per group (and SEM).

To investigate the effects of the transferred bacteria on the physiology of the recipient rats, daily assessment data were examined (Fig. 2). Analysis revealed no discernible differences in the development of food intake, BW, or RWA between the groups during the different time periods (Fig. 2A–C). Moreover, the food utilization efficiency did not significantly change within the groups over time (Fig. 2D).

### Microbial composition was influenced by both ABX and FMT

**Fig. 3:**
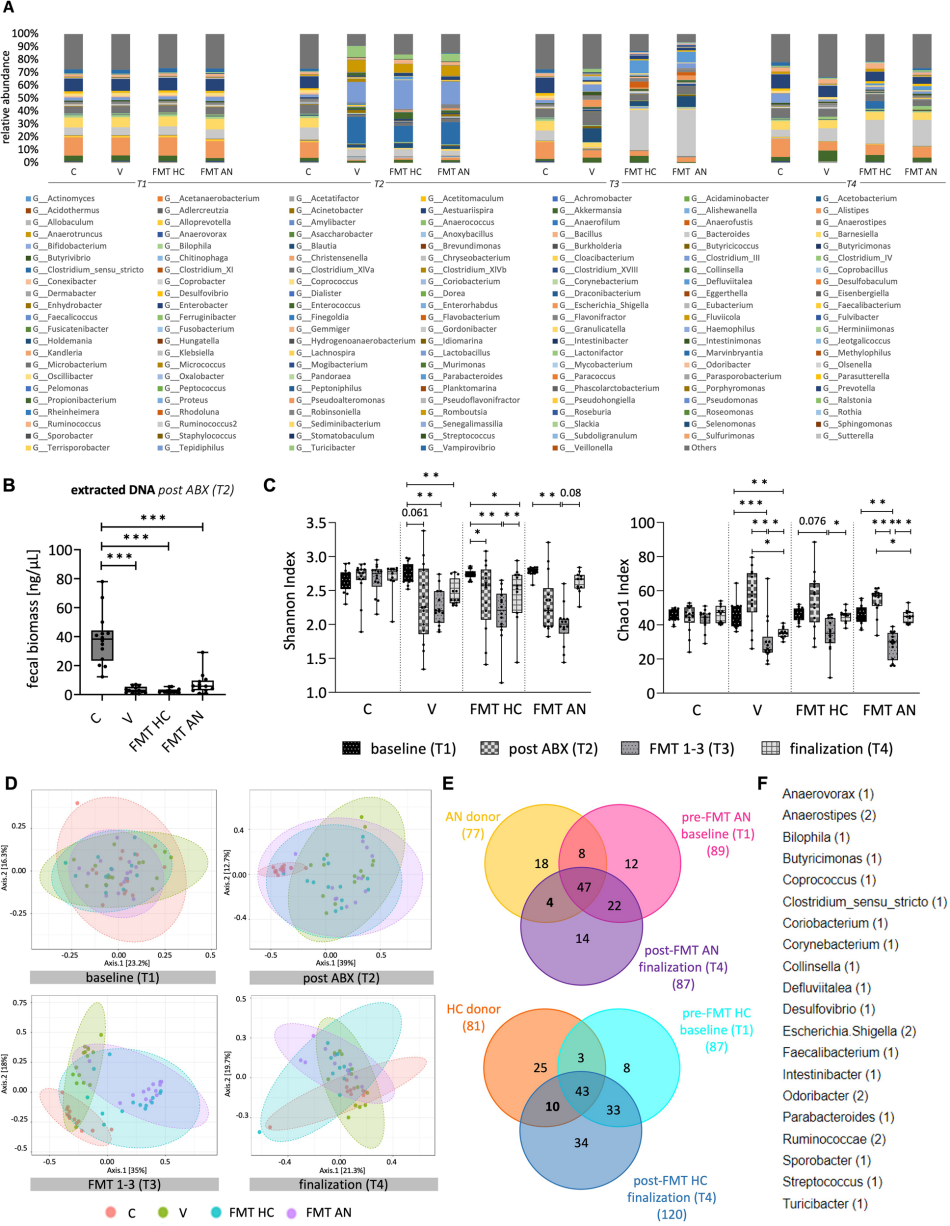
Microbial composition of the four experimental groups at different time points. **A** Relative taxa abundance as mean value per group. Wilcoxon testing of the four experimental groups at four distinct time points (baseline (T1), after ABX (T2), after FMT 1–3 (T3), at finalization (T4)). **B** Total fecal biomass based on extracted DNA measurements after ABX treatment (T2). **C** Alpha diversity based on Shannon (left) and Chao1 (right) indices. **D** Illustration of beta diversity of all groups at all time points using Bray-Curtis dissimilarity. **E** Overlapping amplicon sequence variants (ASVs) comparing human donor microbiome (yellow/orange), recipient rat microbiome at baseline (pink/light blue), and recipient rat microbiome at finalization (purple/dark blue). Illustration of AN group (top) and HC group (bottom). Transferred taxa are highlighted as bold. **F** Transplanted bacterial genera from the AN group. Number of successful transfers into rats in brackets. C=control, V=vehicle, FMT HC=healthy control, FMT AN=anorexia nervosa. p < 0.05 = *, p < 0.01 = **, p < 0.001 = ***.

The development of the bacterial compositions based on the taxa abundance of core microbiota can be seen in Fig. 3A.

Alterations in the composition were visible in the alpha diversity (Fig. 3B). All four groups exhibited comparable alpha diversities at baseline (T1). Following ABX (T2), a reduction in the Shannon index was observed compared with that at baseline (T1 vs. T2 V p = 0.061; HC p = 0.026; AN p = 0.151). Accordingly, reductions in the total fecal biomass were detected in all three ABX-treated groups (p ≤ 0.001, Fig. 3B). In contrast, the Chao1 index tended to increase at T2. Following three FMT administrations (T3), a reduction in alpha diversity was still visible in the ABX-treated groups compared to their baseline (T1 vs. T3 Shannon: V p = 0.002, HC p = 0.002, AN p = 0.007; Chao: V p ≤ 0.001, HC p = 0.076; AN p = 0.002). At finalization (T4), the Shannon index of the vehicle and the FMT HC groups were still reduced, whereas the AN-derived microbiome reached basal levels (T1 vs. T4 V p = 0.008, HC p = 0.022, AN p = 0.129). In contrast, although the Chao1 index of the vehicle group was significantly lower than that at baseline, neither of the two FMT groups had a statistically significant effect at this time point (T1 vs. T4 V p = 0.006, AN p = 1, HC p = 1). Nevertheless, compared with T3, after three FMTs, alterations in the alpha diversity of both FMT groups at finalization (T4) were notable (T3 vs. T4 Shannon HC p = 0.009, AN p = 0.08; Chao1 HC p = 0.011, AN p = 0.003; see Fig. 3C).

Furthermore, beta diversity, which was calculated using the Bray‒Curtis dissimilarity index, was significantly altered within the three ABX groups (Fig. 3D). Compared with the control groups, the ABX-treated groups presented a shift in beta diversity after administration (T2) (PERMANOVA p = 0.005). Similarly, after three FMT administrations (T3), the controls clustered separately (Fig. 3D, PERMANOVA V p = 0.009, HC p = 0.002, AN p = 0.012). When the vehicle group was compared with both FMT-treated groups, clusters were detected (HC p = 0.01, AN p = 0.003). At finalization (T4), no significant deviations in beta diversity were identified.

Additionally, pairwise analysis of the most abundant bacterial taxa revealed alterations in four genera after the administration of three FMT treatments (T3) (Wilcoxon test, T3 HC vs. AN *Anaerovorax* p = 0.003, *Blautia* p = 0.02, *Butyricicoccus* p = 0.03, *Intestinibacter* p = 0.04). In contrast, at finalization (T4), all four groups had similar compositions.

### Overlapping ASVs indicate successful transfer of FMT-transplants

Alterations of genera observed in the fecal samples of the both FMT groups are presented in Fig. 3E + Fig. 3F. For this evaluation, the donor microbiome was compared to the composition of the rats at baseline (T1) and at finalization (T4). ASVs of the human microbiome that could be identified in the rats exclusively following transplantation (T4) were classified as successfully transplanted species (highlighted in Fig. 3E). Among these, four ASVs were observed in the FMT AN group (4.6%), and ten were observed in the HC group (8.3%). To reduce the influence of donor-specific variance, subsequent analyses were conducted on a per-donor basis resulting in a maximal overlap of 15% of the ASVs. This resulted in a total of 20 successfully transplanted ASVs in the AN group, with 14 ASVs being identified in at least three of the four patients with AN. The transplanted ASVs are shown in Fig. 3F.

### Impact of ABX but not FMT treatment on hippocampal glial cell gene expression

**Fig. 4:**
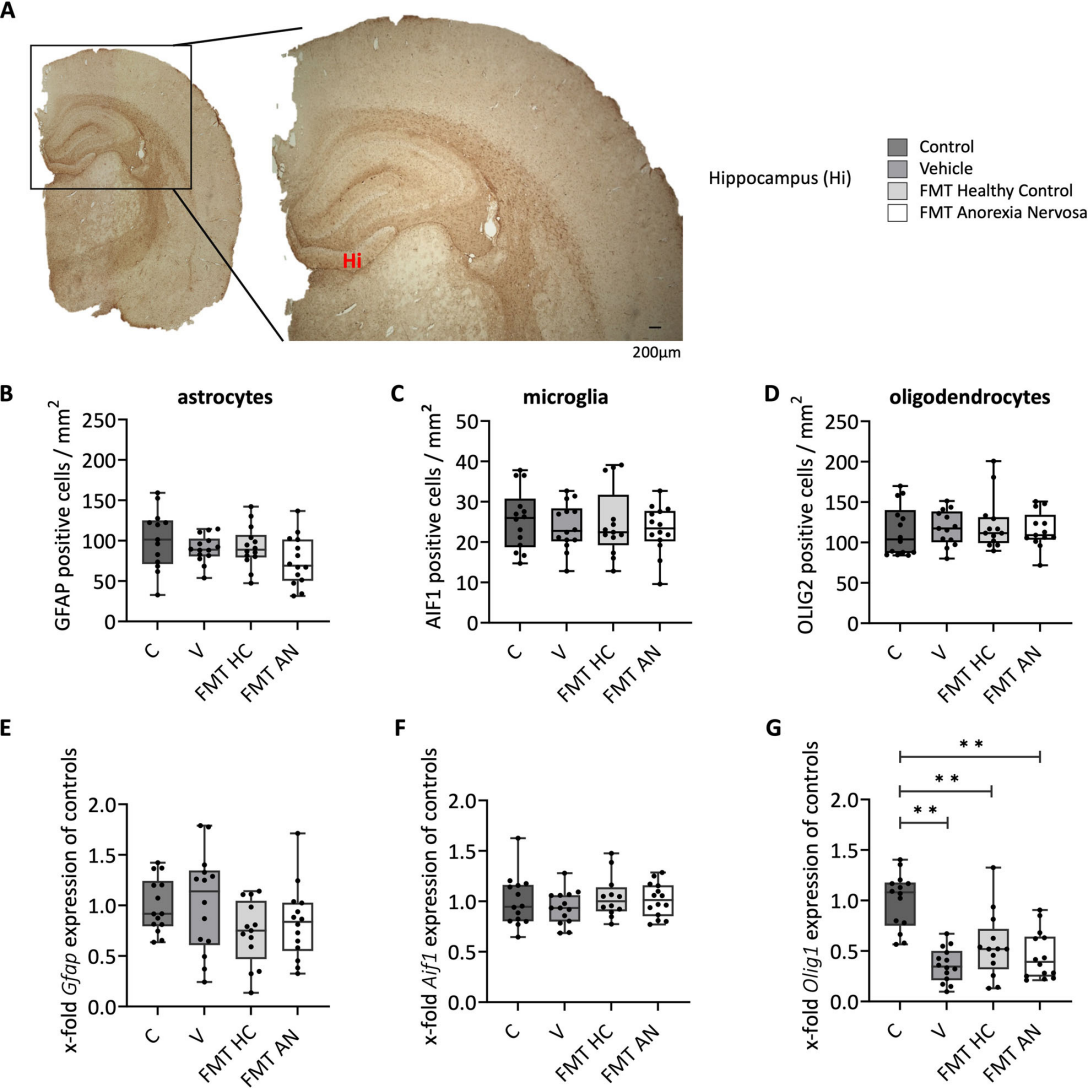
Alterations of brain volume and glial cell composition. **A** Exemplary illustration of merged brain slice with depiction of the hippocampal formation (Hi). **B**–**D** Results of cell count of brain glial cells after immunohistochemical staining in the hippocampus as mean values in cells/mm^2^: astrocytes (targeted via glial fibrillary acidic protein/Gfap, (**B**)), microglial cells (targeted via ionized calcium-binding-adapter molecule/Aif1, (**C**)), and oligodendrocytes (targeted via oligodendrocyte-transcription-factor 2/Olig2, (**D**)). **E**–**G** Gene expression of brain glial cells in hippocampus as x-fold expression of controls mean value: Gfap (**E**), Aif (**F**) and Olig1 (**G**). Statistical significance: ANOVA and Bonferoni post-hoc: p < 0.01 = **.

All four groups presented similar hippocampal glial cell composition (Fig. 4A–D). In terms of gene expression (Fig. 4E–G), a decrease in *Olig1* expression was detected in the Hi of the ABX-treated groups compared with the control (Fig. 4G, p ≤ 0.001; V/HC/AN, p ≤ 0.001), whereas the expression levels of *Gfap* and *Aif1* did not differ.

### FMT-induced alterations in the hippocampal gene expression of plasticity markers and cytokines

**Fig. 5:**
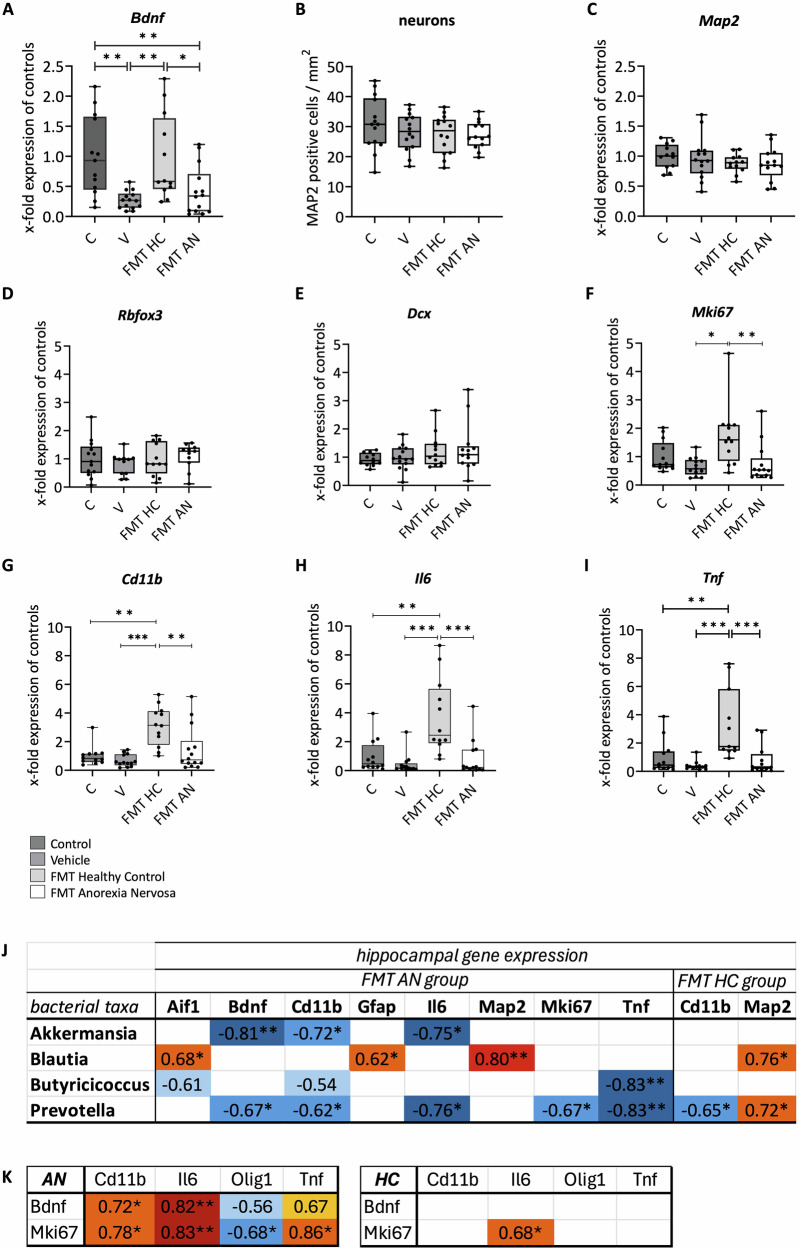
Alterations of hippocampal plasticity and inflammatory parameters. **A** Gene expression of brain-derived neurotrophic factor (Bdnf) in the hippocampus. **B** + **C**) Immunohistochemical staining results of cell count (**B**) and gene expression (**C**) of the postmitotic neuronal marker microtubule-associated protein 2 (Map2) in the hippocampus. **D–I** Gene expression of neuron-specific nuclear protein (Rbfox3, **D**), Doublecortin (Dcx, **E**) and Kiel-antigen 67 (Mki67, **F**) in the hippocampus. Gene expression of proinflammatory cytokines cluster of differentiation 11 (Cd11b, **G**), interleukin 6 (Il6, **H**), and tumor necrosis factor (Tnf, **I**) in the hippocampus. **J** Pairwise correlation analysis of hippocampal gene expression and selected bacterial taxa in the two FMT groups (AN vs. HC) based on Spearman coefficient. **K** Correlation analysis of cytokine-brain cell-associations based on Spearman coefficient in the two FMT groups. C=control, V=vehicle, FMT HC=healthy control, FMT AN=anorexia nervosa. Statistical significance: ANOVA and Bonferoni post-hoc/Spearman correlation test p < 0.05 = *, p < 0.01 = **, p < 0.001 = ***.

To further elucidate the regulatory impact of FMT on neuronal development, the gene expression of neuronal markers in the Hi was evaluated. Compared with that in the control group, significantly lower gene expression of brain-derived neurotrophic factor (*Bdnf*) in the V and FMT AN groups was detected (C vs. V p = 0.004, vs. AN p = 0.007), whereas the gene expression in the FMT HC group did not differ from that of the control group (HC vs. C p = 1, vs. V p = 0.007, vs. AN p = 0.012; Fig. 5A). When the gene expression of neuronal markers was examined, neither the number of MAP2-positive cells nor the gene expression of *Map2*-associated genes exhibited divergent patterns (Fig. 5B + Fig. 5C). Additionally, *Rbfox3* (a marker for postmitotic neurons) and *Dcx* (markers for premitotic neurons) did not show altered expression patterns (Fig. 5D + Fig. 5E).

Compared with the vehicle and FMT AN groups, the FMT HC group presented significantly increased gene expression of *Mki67* (V p = 0.017, AN p = 0.005; Fig. 5F). No significant alterations toward controls were detected. In terms of inflammation, the gene expression levels of *Tnf, Il6*, and *Cd11b* were greater in the FMT HC group than in the control, vehicle, and FMT AN groups (*Tnf* p ≤ 0.001, C p = 0.003, V p ≤ 0.001, AN p ≤ 0.001; *Il6* p ≤ 0.001, C p = 0.006, V p ≤ 0.001, AN p ≤ 0.001; *Cd11b* p ≤ 0.001, C p ≤ 0.001, V p ≤ 0.001, AN p = 0.003; Fig. 5G–I).

### Exploratory correlation analyses revealed associations between microbial alterations and hippocampal plasticity markers and cytokines

Negative correlations between the genus *Akkermansia* and the gene expression of *Bdnf*, *Il6* and *Cd11b* in the FMT AN group in the Hi was detected in our analysis (p = 0.003, p = 0.01, p = 0.01). Furthermore, the genus *Blautia* could be associated with gene expression of astrocytes and microglia (*Gfap* (p = 0.04) and *Aif1* (p = 0.02)) and neurons in the FMT AN-treated group and, to some extent, in the FMT HC-treated group (*Map2* (p = 0.006, p = 0.02)). Finally, the abundance of the genus *Prevotella* was negatively correlated with the gene expression levels of the inflammatory markers *Il6* (p = 0.02), *Cd11b* (p = 0.047) and *Tnf* (p = 0.008) as well as the proliferation genes *Bdnf* (p = 0.03) and *Mki67* (p = 0.04) in FMT AN-treated animals. These negative associations with inflammation were also partially detected in the genus *Butyricicoccus* (*Aif1* p = 0.052; *Cd11b* p = 0.093; *Tnf* p = 0.008).

A negative correlation of the genus *Prevotella* with *Cd11b* was also detected in the FMT HC group (p = 0.049). In contrast, a positive association with the neuronal marker *Map2* (p = 0.04) was observed in FMT HC rats.

Selected bacterial taxa that have been associated with neuronal and inflammatory involvement in mental disorders are illustrated in Fig. 5J and the Supplementary Table 4. Correlation plots can be taken from the Supplementary File 3.

In the FMT HC group, the expression of *Mki67* was positively associated with the expression of *Il6*-associated genes (p = 0.04). In the FMT AN group, the gene expression levels of both the *Mki67* and *Bdnf* genes were positively correlated with *Cd11b*, *Il6* and *Tnf* gene expression (p = 0.01, p = 0.02; p = 0.008, p = 0.006; p = 0.01, p = 0.06, respectively). Negative correlations between *Mki67* (and *Bdnf* at a trend level) and *Olig1* gene expression were detected (p = 0.03, p = 0.08). The correlation results are depicted as a heatmap in Fig. 5K. The results from the control and vehicle groups are summarized in the Supplementary Table 4.

## Discussion

This study investigated the influence of FMT from patients with AN vs. HCs on the induction of an AN-like phenotype in rats. As one of the first studies, repeated fecal transplants into non-GF rodents were performed and their impact on hippocampal cell neogenesis and inflammation were studied. Primarily, the ABX administration itself as well as the following FMT-treatment resulted in a notable shift in the microbial composition of the rats. ABX-induced dysbiosis were partially restored after FMT, and limited bacterial transfer from donors successfully achieved. Nevertheless, interventions did not induce an AN-like phenotype in the recipients. In the hippocampus, ABX treatment reduced the gene expression in oligodendrocytes. Furthermore, the expression of *Bdnf*, a marker of inflammation and proliferation, was reduced by ABX and selectively increased by HC but not AN FMT.

### Transferring gut microbes: the impact of fecal transplants

With respect to FMT for AN, seven animal studies yielding disparate outcomes are available. The studies by Hata et al., Glenny et al., Fan et al., and Gabriel-Segard et al. [51, 52, 55, 58] all conducted FMTs from human donors into germ-free (GF) mice. Each study revealed alterations in the microbiota composition of the mice that received FMTs from patients with AN given a (partial) transfer efficiency. Only Hata et al. and Fan et al. described reductions in body weight gain, diminished food intake, and increased anxiety-like behavior, accompanied by modifications in neurotransmitter levels in the brains of transplanted mice [51, 52]. Notably, in the study of Fan et al.[51], this occurred only upon food restriction for the animals. However Gabriel-Segard et al. [58] did not find body weight alterations in rodents of the AN group, further AN-like symptoms e.g. food restriction, anxiety-like behavior, physical hyperactivity and inflammatory responses were seen. Interestingly, also a dysfunction of ovarian follicles and the liver were detected.

In a similar approach to our own, both, Maschek et al. and Kooij and colleagues [53, 54, 59] used rats that had received ABX before FMT. Kooij et al. reported a transfer efficiency of up to 22% of overlapping ASVs with the donor, with high donor-specific variance. Nevertheless, microbial shifts failed to induce changes in the cognitive flexibility, anxiety, or reward signaling of rats [53]. Even in a second study where rats were subjected to the ABA protocol after FMT, no AN-like phenotype was detected [54]. Similarly, Maschek and colleagues failed to induce body weight alterations between treatment groups, eventhough changes in food intake had been detected. Interestingly, this group performed a cross-over design including two FMT periods where rodents were either administred to feces from the same donor group in both FMT phases or from the contrary donor group, respectively (ANAN vs ANHC, HCHC vs HCAN) [59].

Like Kooij et al. [53, 54], in our study, a mixture of ABX was administered to the animals before FMT was started [49], which resulted in a reduction in the Shannon index, indicating successful depletion [49, 53, 69]. Unexpectedly, the Chao1 index increased after ABX treatment in this study. Because Chao1 considers rare bacterial groups with a low abundance, this finding may indicate the proliferation and establishment of rare bacteria that adapt to environmental changes in the niche created by the depletion of major bacterial strains [70]. Specifically, the increased relative abundances of *Clostridium sensu sito*, *Lactobacillus, Turibacter*, and *Romboutisa* after ABX treatment (T2) align with previous findings that *Firmicutes* and spore formers exhibit greater resistance to environmental changes [52–54].

With respect to transplantation efficiency, we identified up to 15% transplanted ASVs; however, similar to the findings of Kooij et al., there was great individual variance within the animals. Other FMT studies using GF-mice in the context of AN were able to transfer up to 50% of bacteria, and FMT studies in the context of *CIostridioides difficile* infection even reached 80% [71–73]. This rather low transfer rate in our study might explain the lack of phenotypic alterations in the rats, as high correlations between the relative mass of transplanted bacteria and the clinical picture have been shown in both clinical and experimental studies.

Nevertheless, several of our successfully transplanted taxa have been previously associated with an “AN microbiome”, as demonstrated by the reviews of Garcia et al. and Anton-Paduraru et al. [14, 16]. Specifically, increases in the genera *Coprococcus, Odoribacter* and *Bilophila* have been detected in several studies [74–77]. The genera *Anaerostipes, Faecalibacterium, Rominococcus, Parabacteroides, Clostridium* and *Collinsella* were dysregulated or reduced in AN [74, 75, 78–80]. Furthermore, Xia et al. reported that the genera *Anaerostipes, Ruminoccoccus* and *Bilophila* are risk factors for AN [77]. Elevated levels of *Ruminoccocus* and *Escherichia/Shigella* were detected in patients with anxiety [81].

Thus, ABX administration might have partially superimposed FMT effects in this study. Moreover, persistent bacterial taxa after ABX treatment augment bacterial competition, hindering the engraftment of new bacteria properly.

### Alterations of oligodendrocyte gene expression

In our model, reduced expression levels of oligodendrocyte-associated genes were observed in the hippocampus. These findings fit well with earlier studies, where microbial dysregulation (e.g., via ABX) led to divergent expression of myelin-associated genes in oligodendrocytes [82]. Further changes in the cell composition of the hippocampus might not be depicted within the time frame of the study, as only a few weeks were considered. However, cell reduction and white matter alterations became apparent with increasing study duration, strengthening the importance of more extensive investigations over longer periods of time. Briefly, our findings suggest ABX-induced alterations since microbial dysbiosis seem to be closely associated with changes in brain gene expression [83, 84].

### ABX-induced reduction in hippocampal plasticity restored by FMT HC

A reduction in *Bdnf* gene expression was observed in the vehicle group, which was likely a consequence of ABX treatment itself or the ensuing change in the microbiota. This finding fits well with a previous study by Möhle et al., who reported that ABX treatment resulted in a reduction of neurogenesis in mice. This effect was restored by HC FMT but not by AN FMT. This finding fits partly with Möhle et al., who reported reversal by probiotics (but not stool transplantation) [69].

BDNF is a relevant factor to the connectivity of axons and synapses [85]. Fröhlich et al. reported a positive correlation between BDNF levels in the Hi and memory learning [86]. BDNF also regulates feeding behavior and energy balance and, thus, might be involved in AN pathophysiology at multiple levels. Consistent with our results, this marker was also reduced in patients with acute AN, and following weight restoration, BDNF levels increased again [87]. However, preclinical studies have failed to show a consistent picture regarding the involvement of AN pathology [88–90]. For this reason, the reduction of *Bdnf* gene expression and further alterations in the hippocampus of the FMT AN group should not be attributed linearly to the AN pathology since crucial mechanisms of AN are yet to be understood in detail and our findings remain model-specific.

Additionally, the FMT HC group presented higher expression of the proliferation marker *Mki67* than the other two ABX-treated groups did. This could indicate an ABX-induced reduction in the gene expression of *Mki67*, akin to the phenomenon observed in *Bdnf* and documented in the literature [91]. This effect appears to be restored after fecal transplantation from HCs but not from patients with AN. Indeed, even some overcompensation appears to have taken place, potentially attempting to rebuild lost cells after ABX treatment, in the HC-transplanted group.

It could also be speculated that BDNF might trigger the proliferation of neuronal stem cells/progenitor cells without full differentiation into glial cells or neurons. This quiescent state, especially in the hippocampus, strongly depends on regulatory factors such as the microenvironment, cytokines, and stressors, which are strengthened by progenitor cell alterations due to changes in the microbial environment, as reported by Möhle et al. [69, 92]. This would also provide a rationale for the lack of alterations in glial cells and neurons despite the dysregulation of proliferation markers. In this context, again, aspects of timing might be crucial, and earlier as well as longer timeframes would be necessary to answer these questions definitively.

### Cytokine gene expression aligns with neurogenesis patterns

The expression levels of the proinflammatory markers *Il-6*, *Tnf* and *Cd11b* were surprisingly similar to those of *Bdnf* and *Mki67*, with significant increases in the FMT HC group and the lowest levels in the two other ABX groups. Starvation is a highly stressful situation for an entire organism, potentially resulting in inflammatory processes. ABA studies recently reported that neuroinflammation and redox balance are influenced by starvation, leading to an increased anti-inflammatory state [88]. Additionally, stress-induced microglial reduction in the hippocampus was previously observed in a rodent model of a chronic unpredictable stress-induced depressive-like condition [93].

In contrast, in adolescent patients with AN, some studies reported reduced levels of the proinflammatory cytokines IL-6, IL-1β, IL7 and IL-12/IL-23p40 [94, 95], whereas Ostrowska et al. reported elevated levels of IL-6 and IL-1β in the blood serum of adolescent patients with AN [96]. In adults, recent large and well-controlled studies have shown that IL-6 is a state marker of AN pathology [97, 98].

Interestingly, depending on the environment, certain proinflammatory cytokines appear to have not only a purely inflammatory effect but also a regenerative function [99–103]. Studies have previously documented an anti-inflammatory response and dysregulation of neuronal networks following the administration of specific ABX, a phenomenon that was also observed in the present study despite the absence of statistical significance [91]. Conversely, the cytokines measured in the FMT HC group but not in the FMT AN group appeared to be overexpressed, which could be interpreted as a sign of recovery from ABX-induced dysregulation. In particular, the effects of IL-6 that extend beyond the inflammatory response have been the subject of extensive discussion in the literature. In adult organisms, cytokines have been demonstrated to play a significant role in regeneration, the migration of various cell types, and neurological functions. In the context of brain aging, the acute upregulation of IL-6 is central to the maintenance of synaptic plasticity, neuronal survival, and axonal regeneration [102]. For example, mice treated with recombinant IL-6 exhibited a substantially elevated proliferation rate and differentiation of neuronal progenitor cells [104].

TNF-α has also been shown to have a protective effect on neuronal regeneration, with oligodendrocytes in particular appearing to be a target. This process prevents the apoptosis of both endothelial and neuronal cells, thus contributing to cell survival and potentially explaining the preserved oligodendrocyte cells despite reduced *Olig1* expression in our ABX animals [103].

Interestingly, the FMT AN group, and to a lesser extent the FMT HC group, presented positive associations of cytokine gene expression and *Bdnf/Mki67* expression, potentially due to greater variations in effects in the AN group, whereas the FMT HC group already partly recapitulated those alterations. To some extent, these correlations were also detected in the V group, supporting this interpretation and an ABX/microbiome interaction with cell regeneration [69]. Because the complex inflammatory regulatory mechanism in AN is most likely multifactorial, understanding the interplay with BDNF might help elucidate pathomechanisms leading to long-lasting inflammatory processes in patients with AN.

### Transplanted bacterial taxa associated with neuronal remodeling and overall health

Although the role of microbial composition in hippocampal neurogenesis remains unidentified, reduced hippocampal volumes were previously observed in patients with acute AN [28]. Thus, the hippocampal alterations observed in both FMT groups represent new findings regarding potentially underlying mechanisms of microbiome‒gut‒brain axis interactions in AN [65, 87, 105]. Numerous bacteria have been associated with markers of the hippocampus, and some bacteria have already been linked to mental illness or neuronal remodeling processes. For example, treatment with *Akkermansia* could be associated with an improvement in mood regulation disorders, a reduction in inflammatory processes and further body weight loss [106, 107]. Interestingly, a negative correlation between cytokine expression in the hippocampus and the abundance of *Akkermansia* was also shown in this study.

The abundance of the genus *Prevotella* was negatively correlated with the gene expression levels of cytokines and proliferative genes in the hippocampi of both FMT AN and HC animals. These findings align with a reduction in cognitive function and neuronal abnormalities in the hippocampi of mice administered *Prevotella* [108], as cytokine expression is closely linked to neuronal remodeling in the hippocampus [109, 110].

Furthermore, the genus *Blautia* has previously been linked to depressive and anxiety-like disorders, as reflected by its overexpression in the FMT AN group compared with the FMT HC group in our study [111]. Additionally, He et al. [112] reported that *Blautia* plays a role in the hippocampal networks of neurons and microglia, which was partially found in our animals. In a mouse model, the depletion of *Blautia* resulted in the development of insulin resistance and increased the risk for obesity, highlighting its involvement in metabolic and nutritional pathways [113].

Similarly, the *Butyricoccus* genus is known to inhibit the expression of proinflammatory cytokines due to butyrate production, especially in patients with inflammatory bowel disease, and is therefore a candidate probiotic drug [114]. In our study, a significantly greater abundance and negative correlations with inflammatory markers and this genus were detected in the FMT AN group, in alignment with these findings.

Additionally, after fecal transplantation, the FMT HC group presented a greater abundance of the *Intestinibacter* genus, a bacterial taxa that was previously associated with a healthier diet and better biomarker profiles, than did the FMT AN group [115, 116].

The alterations observed in the hippocampi of treated animals are indicative, at least in part, of the influence of antibiotic treatment. Furthermore, fecal transplants of HCs but not patients with AN appear to have (over)compensated for some of these effects. Three of the four significantly divergent bacterial taxa between the two transplant groups at T3 (post FMT 1–3) provide initial evidence of the role of the microbiome-gut-brain axis, as they have previously occurred in the context of hippocampal plasticity or metabolism. To achieve a more precise understanding of the involvement of these and other bacteria in the pathophysiology of AN, identifying specific pathways in future studies is essential.

## Limitations and outlook

Our study included the previous depletion of bacteria with ABX before FMT and not germ-free (GF) animals. This administration and the remaining host bacteria in the animals might have interfered with the successful engraftment of additional bacteria. This might explain the reduced transfer efficacy. Nevertheless, we detected successfully transferred species, which may be associated with hippocampal alterations. With respect to AN, previous studies have reported higher success rates after FMT in GF rodents than in animals after ABX treatment [51–55]. However, as GF animals show deficits in behavioral and neuronal development as well as an impairment of immune function, owing to the lack of microbiota during their early development [117, 118], this trade-off must be weighed when choosing the appropriate model for study. Second, a standardized protocol for FMT is lacking. Potential confounders complicating comparability include donor variation, such as age and sex differences, experimental duration, and application patterns of FMT treatment. Further experiments are needed to consolidate a reliable and repeatable protocol. In this context also repeated gavage must be mentioned, as it forms a stressful situation for the animals. Although group effects are minimized by the co-gavage of the controls, it cannot be ruled out that, for example, inflammatory differences are at least partly attributable to the stress response of multiple gavage [119]. As previously outlined, the present study does not encompass long-term effects of FMT. Importantly, these factors should be considered in subsequent studies to investigate the occurrence of structural brain changes over time. Furthermore, the nutritional state of the subjects is a significant variable that was not controlled for in this study and might have influenced the diversity of the microbiota in our experiments [120]. To further elucidate the crucial gut‒brain pathways involved in AN pathophysiology, a further study coupling FMT with reduced feeding to identify the consequences of starvation and altered microbiota combined should be performed. Lastly, it must be highlighted that the presented results are limited to the animal model. These model-specific findings cannot be applied directly to the AN pathology and human patients with AN. Conversely, the transferability of these results and the role of the microbiome-gut-brain axis in the development and maintenance of AN must be further investigated in future human studies based on key alterations discussed in this work.

## Conclusion

In summary, the present study revealed a notable impact of FMT from patients with AN and HCs on the microbial composition of recipient rats, with successful transfer of bacterial taxa. Hippocampal plasticity as well as inflammatory and regenerative patterns were reduced after ABX and could be selectively reversed with fecal material from HCs but not from patients with AN. These findings highlight the distinct role of the hippocampus in the microbiome‒gut‒brain axis and potentially in neuronal restitution after ABX/microbiota depletion-induced dysregulation. Furthermore, specific bacteria that had already been attributed to inflammatory or neuronal processes and dietary patterns in previous studies could be associated with these hippocampal changes. These results help us understand the pathobiology of microbiome‒gut‒brain interactions in AN by identifying bacterial taxa that are crucial for AN pathology, being producers of metabolites that impact brain cell composition and signaling processes. Identification of these metabolites working as biomarkers, and the according signaling pathways will enable future research to focus on specific targets when trying to influence AN development. In this context bacterial taxa relevant in the AN pathophysiology might be combined as synthetic consortia mimicking the microbial composition in AN. With regard to FMT, novel protocols including repeated gavages and examination of different study outcomes add to a greater comprehension of variables crucial for an effective fecal transfer. In further preview, these findings will be relevant when aiming to influence patients´ microbial composition as innovative treatment strategy of AN. Combining these insights with future studies that include food restriction and microbiome-targeted interventions shows great potential for further comprehension of the underlying processes and ultimately for the development of additional treatment components for patients with AN.

## Supplementary information

16s rRNA sequencing

Representative images of histolgical stainings (GFAP, AIF1, OLIG2, MAP)

Correlation plots of association of bacterial taxa abundance with hippocampal gene expression of FMT AN group and FMT HC group

List of applied antibodies

List of applied primers

numbers of animal samples included per group in each analysis as n(analysis)/n(total)

Association of bacterial taxa abundance with hippocampal gene expression of FMT AN group (left) and FMT HC group (right)

Correlation of proliferatory markers and gene expression in the hippocampus of controls (C) and antibiotics treated group (V)

Figure legends of Supplementary information

## Data Availability

All of our data is available from the corresponding author upon reasonable request.
