# Supplementary material for: From gut to brain: effects of fecal microbiota transplants from humans to rats on hippocampal gene regulation - a study on anorexia nervosa
Source: Transl Psychiatry. 2026 Apr 30;16:238. doi: 10.1038/s41398-026-04056-9 (PMC13133121; doi:10.1038/s41398-026-04056-9)
Supplement: Supplementary file 1 — 16s rRNA sequencing [file 41398_2026_4056_MOESM1_ESM.pdf]

DNA extraction and 16s rRNA gene sequencing and processing

DNA was extracted using the QIAamp Fast DNA Stool Mini Kit, automated on the QIAcube (QIAGEN, Hilden, Germany). Therefore, each pellet was transferred to a Power Bead tube containing 1 ml of InhibitEx buffer. Homogenization was then performed using a SpeedMill PLUS (Analytik® Jena, Jena, Germany) for 45 s at 60 Hz. Samples were heated to 95°C for 5 min and centrifuged at 10,000 rpm for 1 min. A volume of 200 µl of the resulting supernatant was placed in the QIAcube for automated DNA isolation. Finally, the DNA was eluted with 200 µl TE buffer.

The V3/V4 variable regions of the 16S rRNA gene were amplified in a one-step PCR using the primer pair 341F-806R (dual barcoding approach; primer sequences: 5'-CCTACGGAGGCAGCAG-3' and 5'-GGACTACHVGGGTWTCTAAT-3'). After checking the presence of PCR products by capillary electrophoresis, the products were stored at -20 °C. To purify the PCR products and ensure an equal amount of each product for the sequencing pool, a normalization assay was performed using the SequalPrep™ Normalisation Plate Kit (Thermo Fisher Scientific, Darmstadt, Germany). The Qubit™ 2.0 Fluorometer (Thermo Fisher Scientific, Darmstadt, Germany) was used to quantify DNA using the dsDNA Broad Range program. Depending on the amount of DNA, the eluates were pooled equimolarly and stored at -20°C until sequencing.

16S rRNA sequencing was performed at the Next Generation Sequencing (NGS) laboratory of the Competence Centre for Genomic Analysis (CCGA) in Kiel, Germany. Sequencing was performed using paired-end MiSeq® sequencing (Illumina) with a reading length of 2 x 300 bp. Sequencing was performed on the MiSeq platform (MiSeqFGx; Illumina) using the MiSeq Reagent Kit v3. The demultiplexing settings were configured to allow for zero mismatches in the barcode sequences. Quality control, trimming and filtering of sequencing files was performed using an established pipeline at IKMB (https://github.com/mruehlemann/ikmb_amplicon_processing). The merged reads were then assigned to the RDP database.
