# Supplementary figures and images for: From gut to brain: effects of fecal microbiota transplants from humans to rats on hippocampal gene regulation - a study on anorexia nervosa
Source: Transl Psychiatry. 2026 Apr 30;16:238. doi: 10.1038/s41398-026-04056-9 (PMC13133121; doi:10.1038/s41398-026-04056-9)

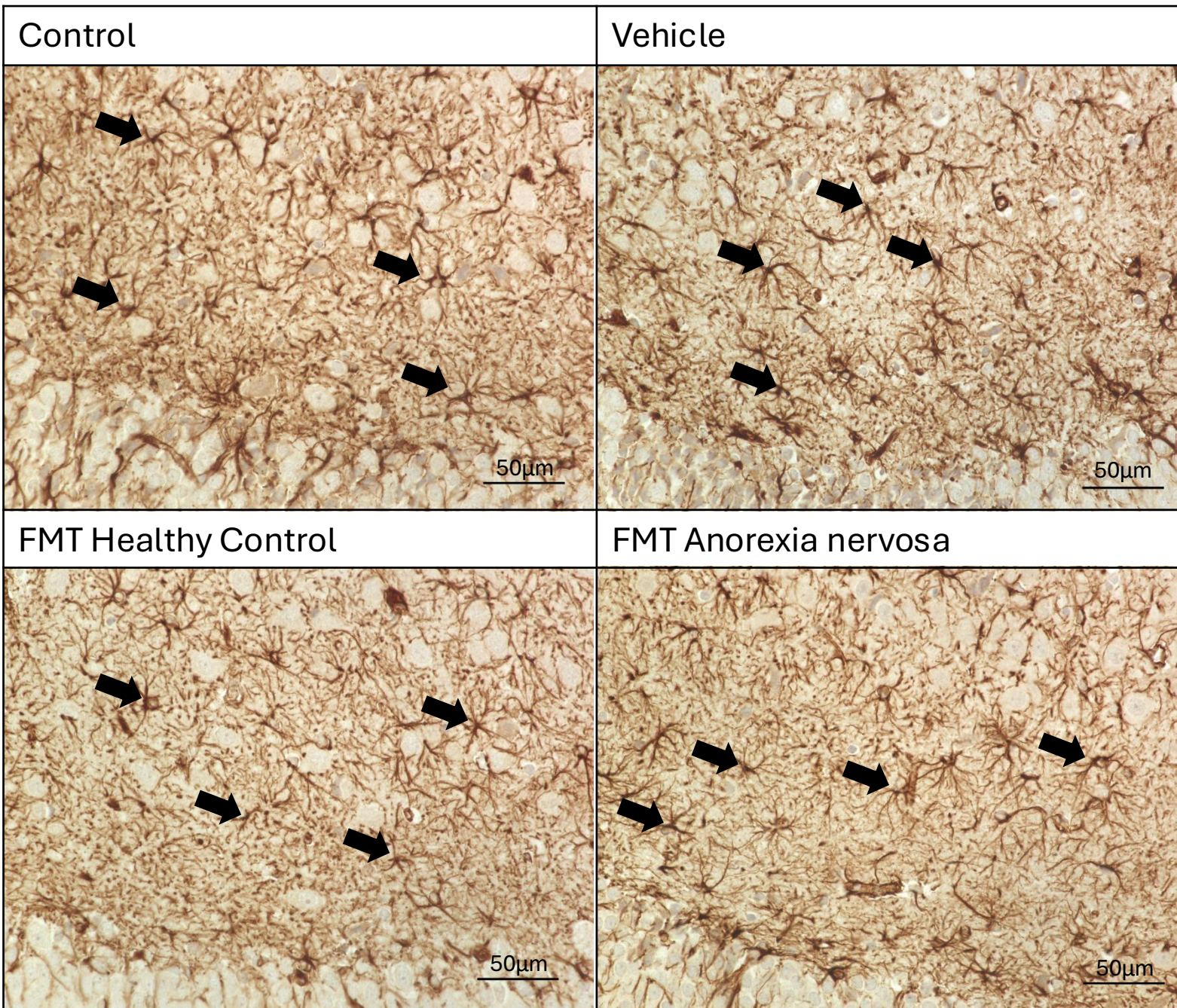

**GFAP 40x zoom**

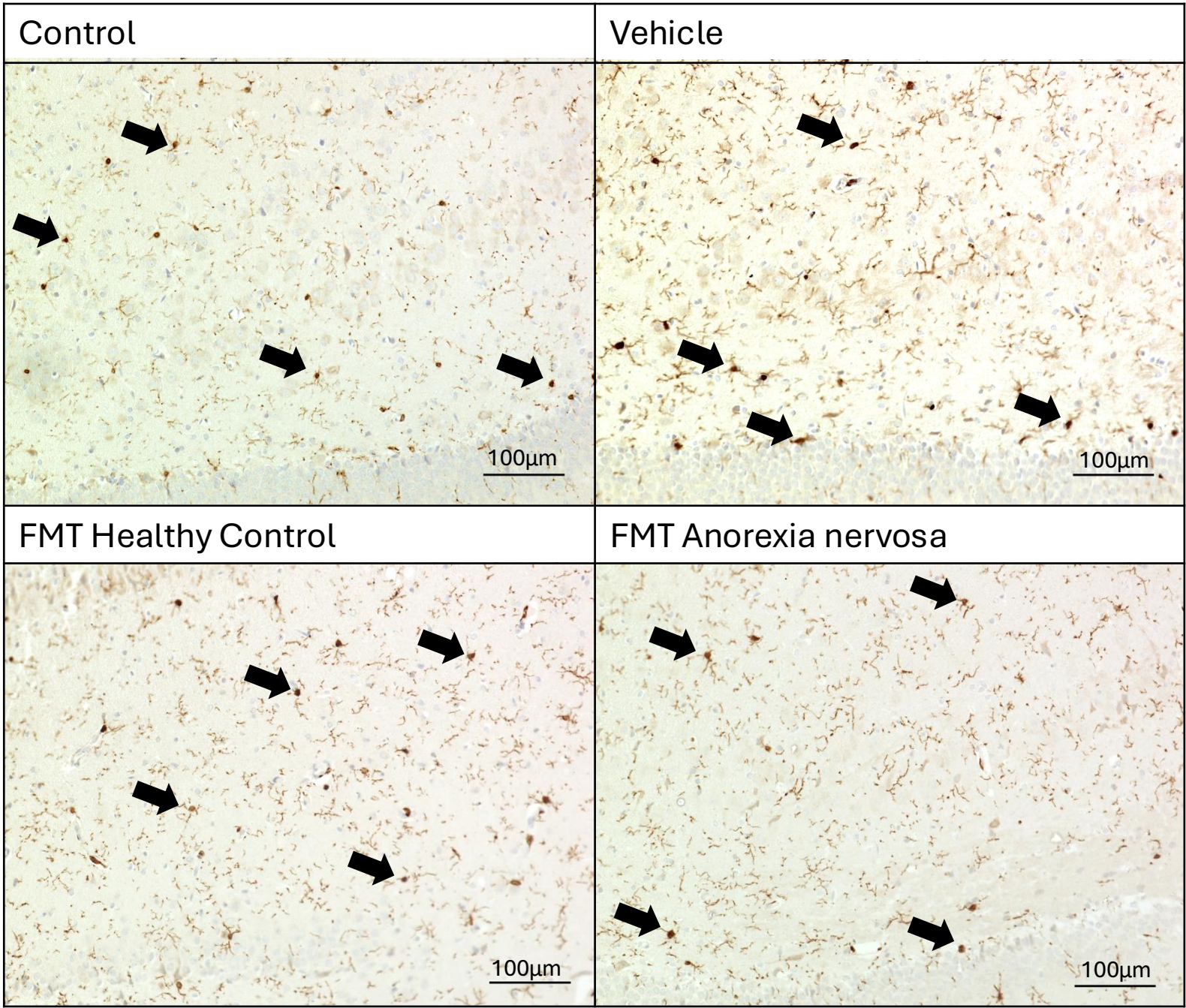

**AIF12 20x zoom**

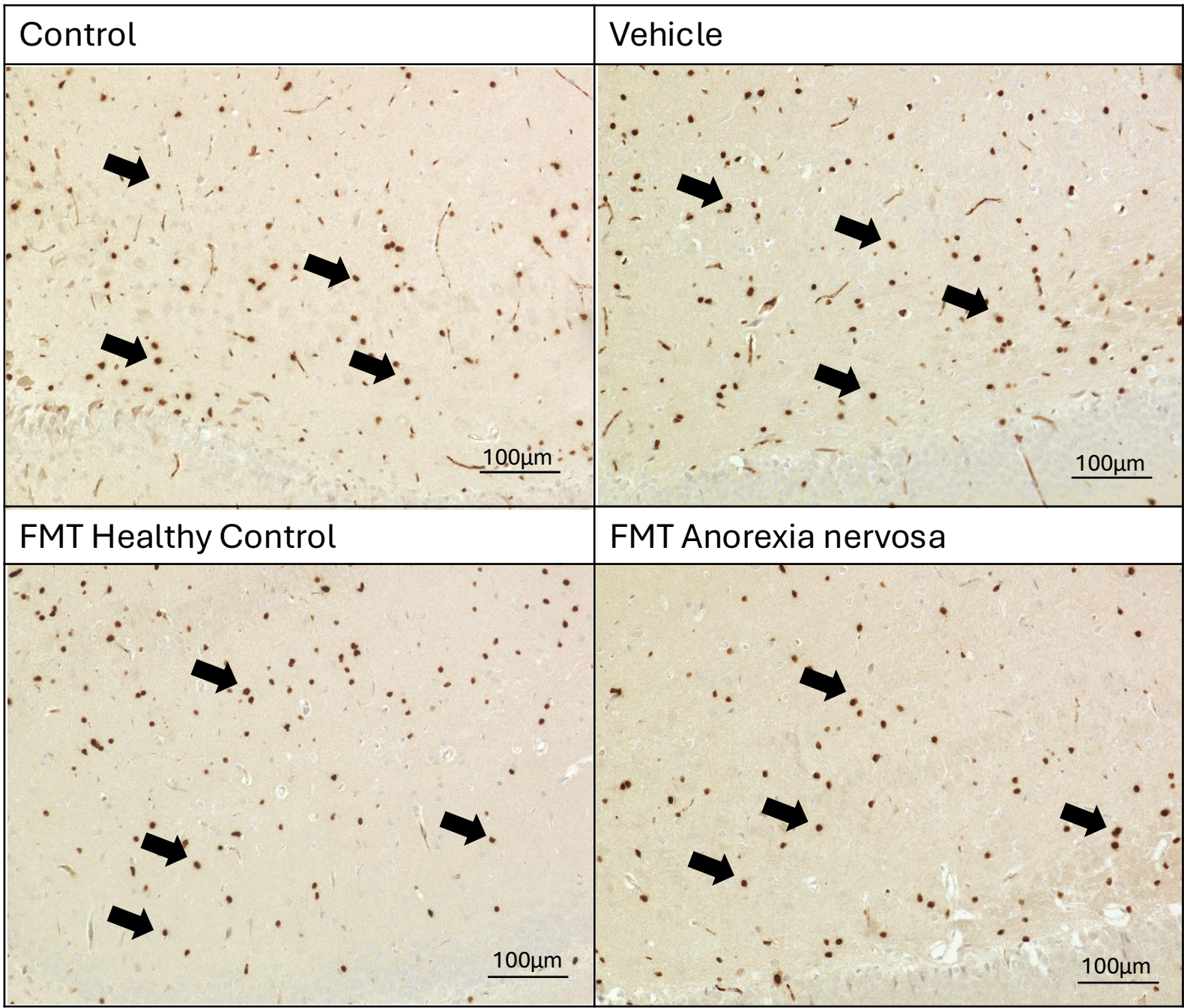

**OLIG2 20x zoom**

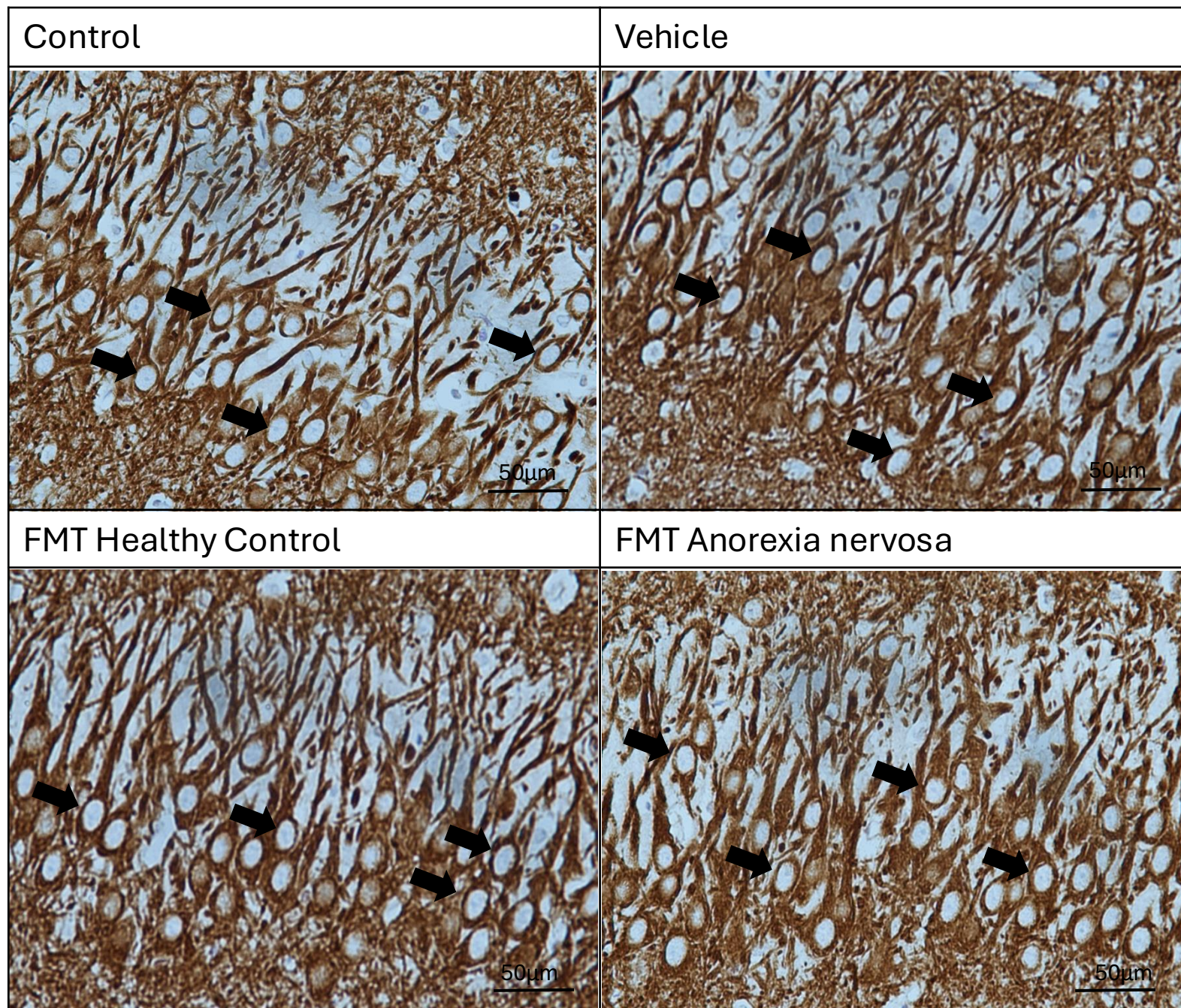

**MAP 40x zoom**

Supplement: Supplementary file 2 — Representative images of histolgical stainings (GFAP, AIF1, OLIG2, MAP) [file 41398_2026_4056_MOESM2_ESM.pdf]
