# Supplementary material for: From gut to brain: effects of fecal microbiota transplants from humans to rats on hippocampal gene regulation - a study on anorexia nervosa
Source: Transl Psychiatry. 2026 Apr 30;16:238. doi: 10.1038/s41398-026-04056-9 (PMC13133121; doi:10.1038/s41398-026-04056-9)
Supplement: Supplementary file 3 — Correlation plots of association of bacterial taxa abundance with hippocampal gene expression of FMT AN group and FMT HC group [file 41398_2026_4056_MOESM3_ESM.pdf]

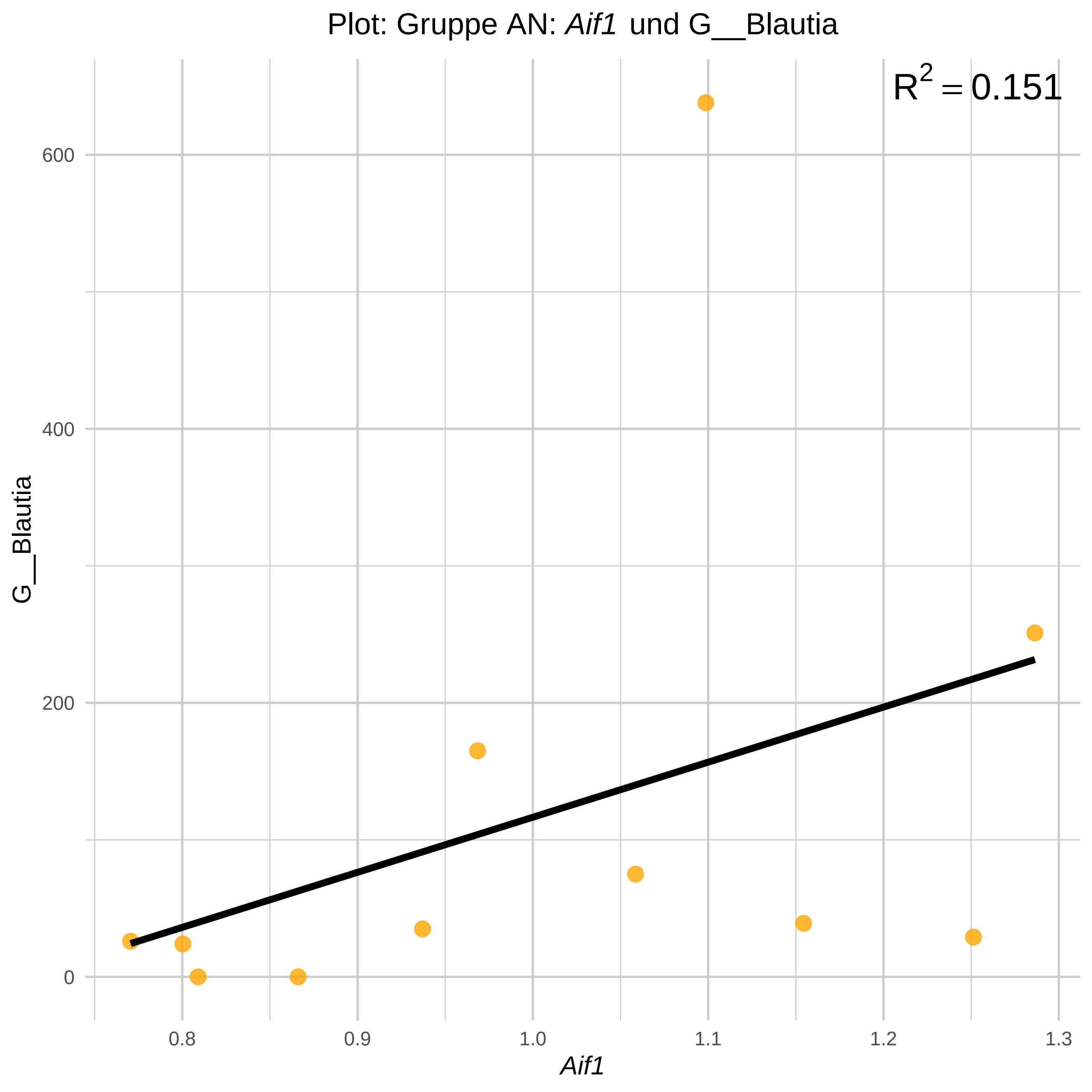

Plot: Gruppe AN: *Aif1* und G__Blautia
$R^2 = 0.151$
600
400
200
0
G__Blautia
0.8
0.9
1.0
1.1
1.2
1.3
Aif1

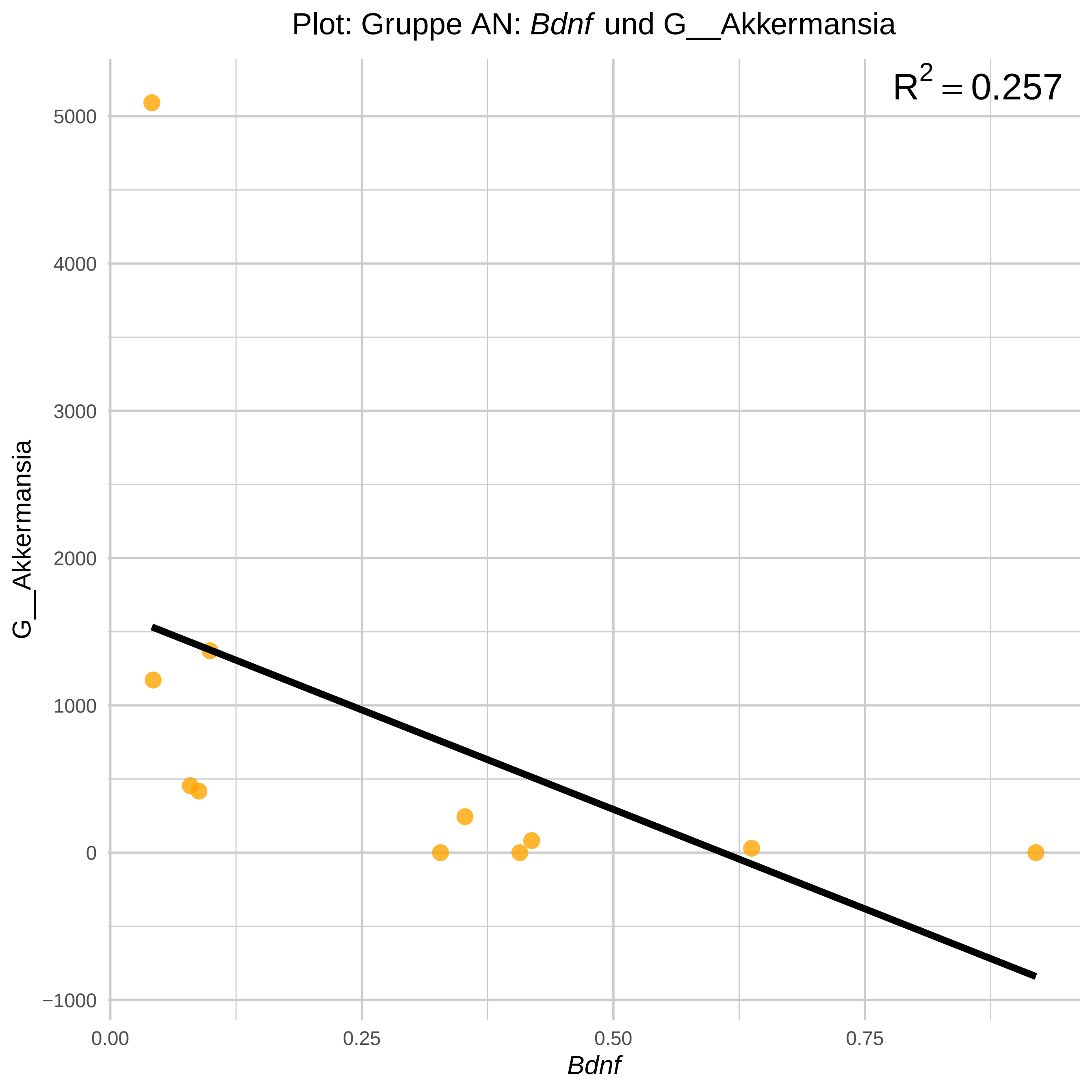

Plot: Gruppe AN: *Bdnf* und G__Akkermansia
$R^2 = 0.257$
5000
4000
3000
2000
1000
0
−1000
G__Akkermansia
0.00
0.25
0.50
0.75
Bdnf

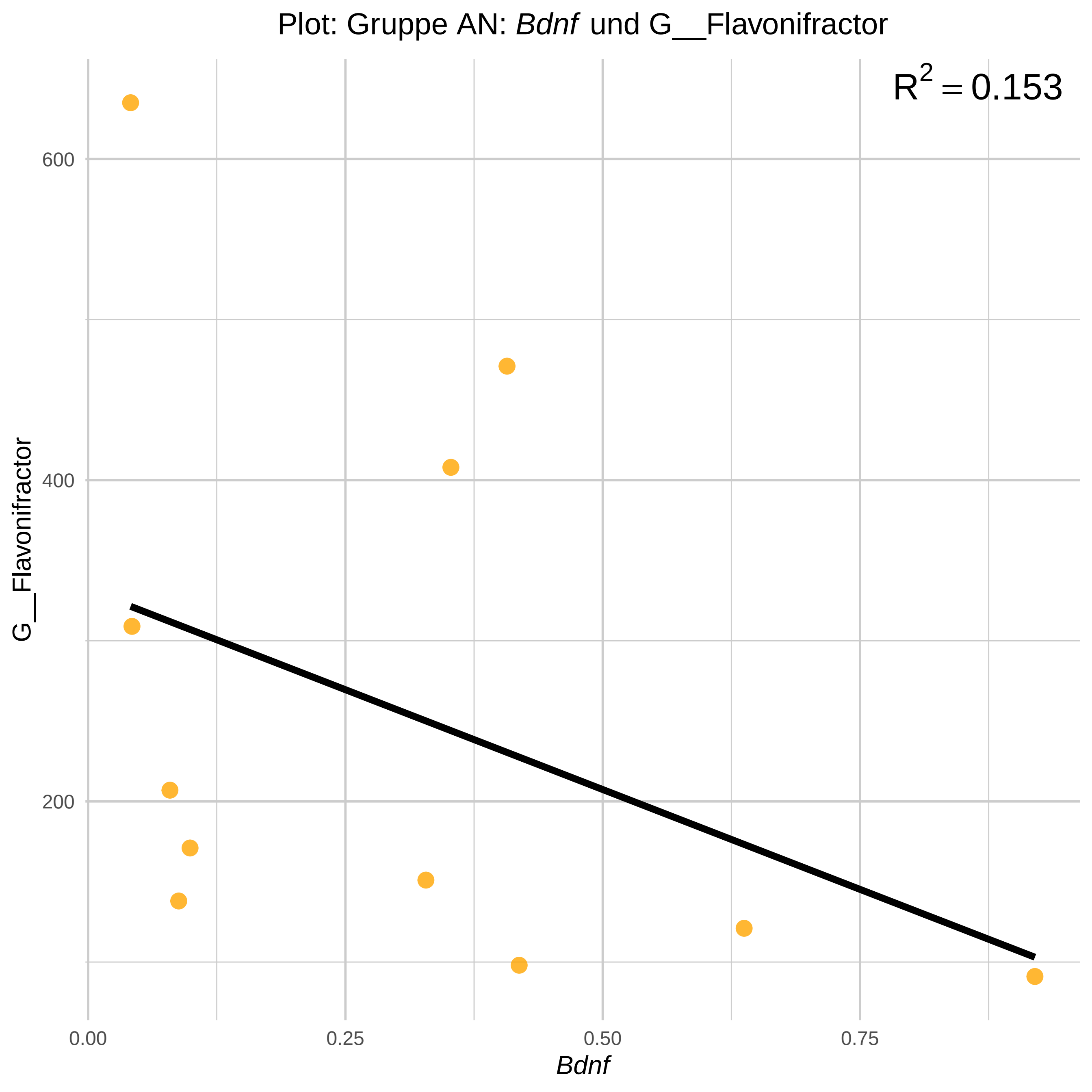

Plot: Gruppe AN: Bdnf und G__Flavonifractor
R2 = 0.153
600
400
200
G__Flavonifractor
0.00
0.25
0.50
0.75
Bdnf

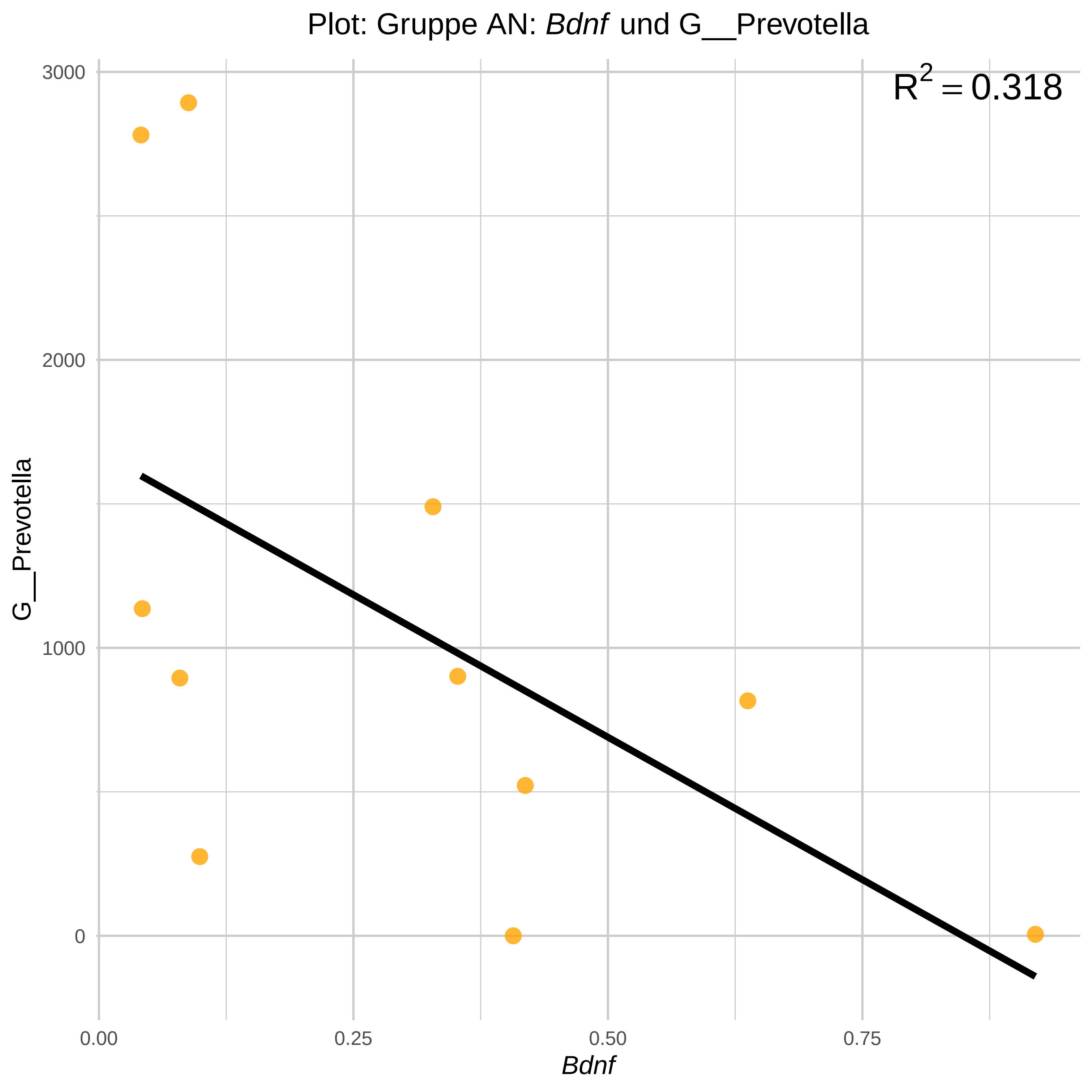
Plot: Gruppe AN: Bdnf und G__Prevotella
$R^2 = 0.318$
3000
2000
1000
0
G__Prevotella
0.00
0.25
0.50
0.75
Bdnf

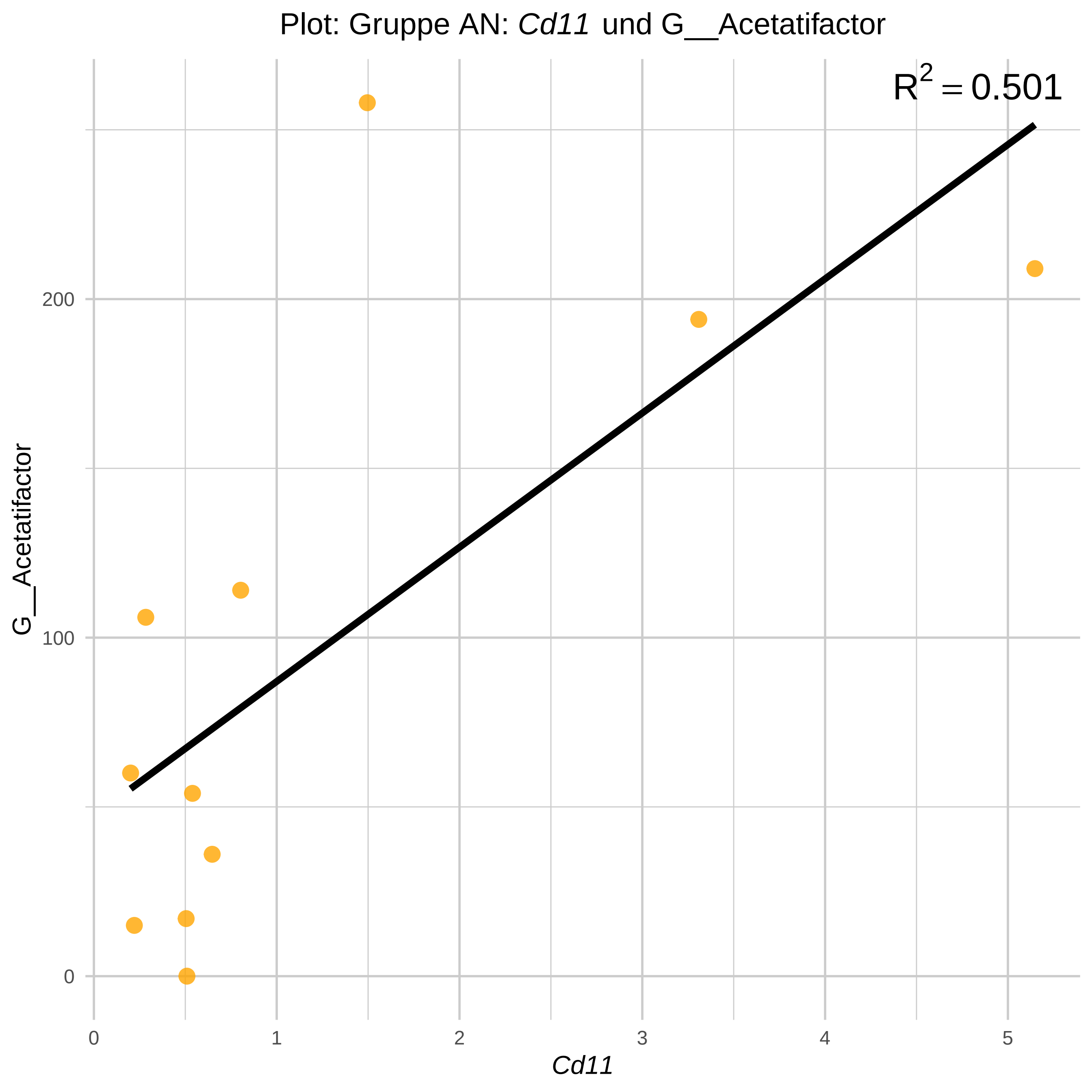

Plot: Gruppe AN: *Cd11* und G__Acetatifactor
$R^2 = 0.501$
G__Acetatifactor
200
100
0
0
1
2
3
4
5
Cd11

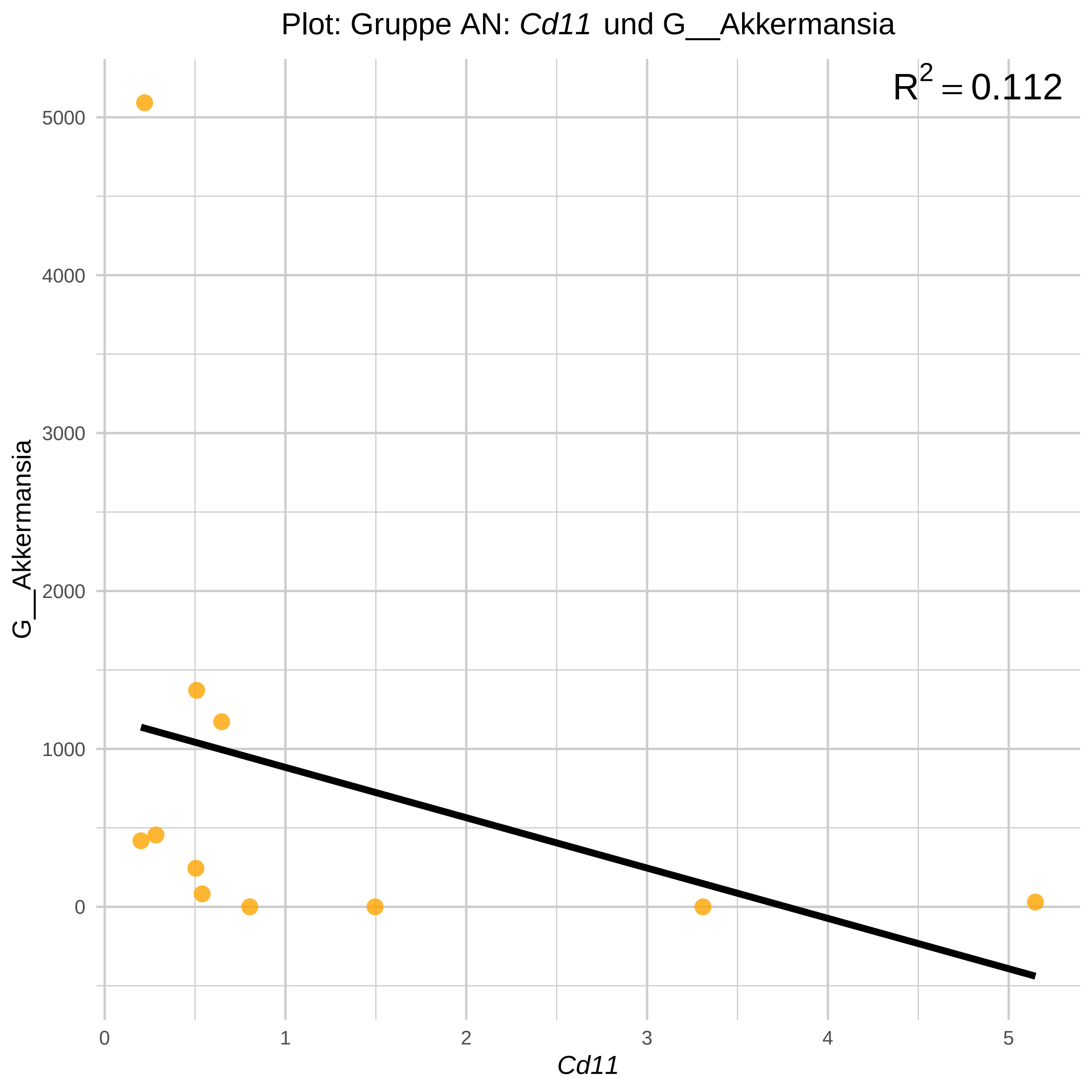

Plot: Gruppe AN: *Cd11* und G__Akkermansia
$R^2 = 0.112$
5000
4000
3000
2000
1000
0
G__Akkermansia
0
1
2
3
4
5
Cd11

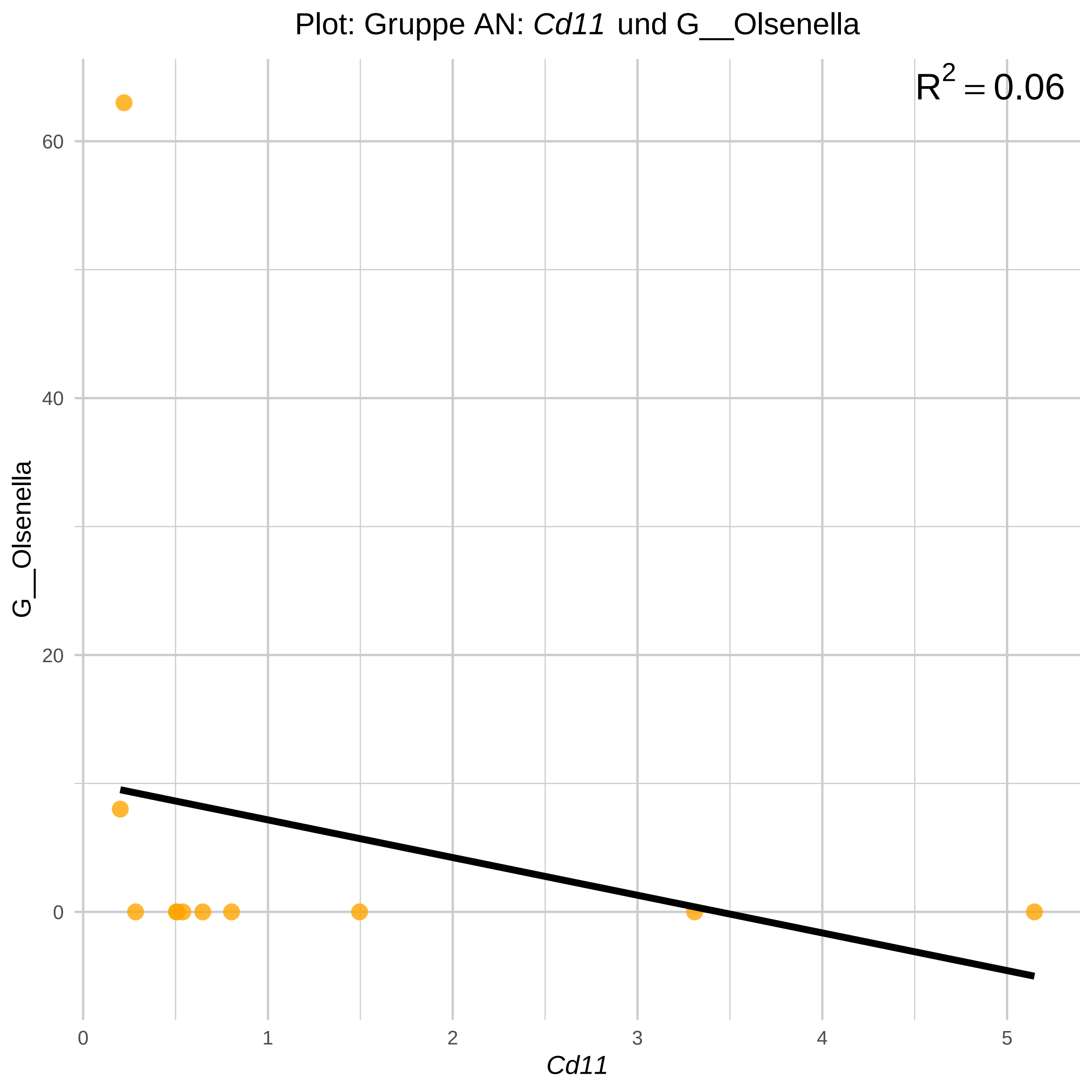

Plot: Gruppe AN: *Cd11* und G__Olsenella
$R^2 = 0.06$
60
40
20
0
G__Olsenella
0
1
2
3
4
5
Cd11

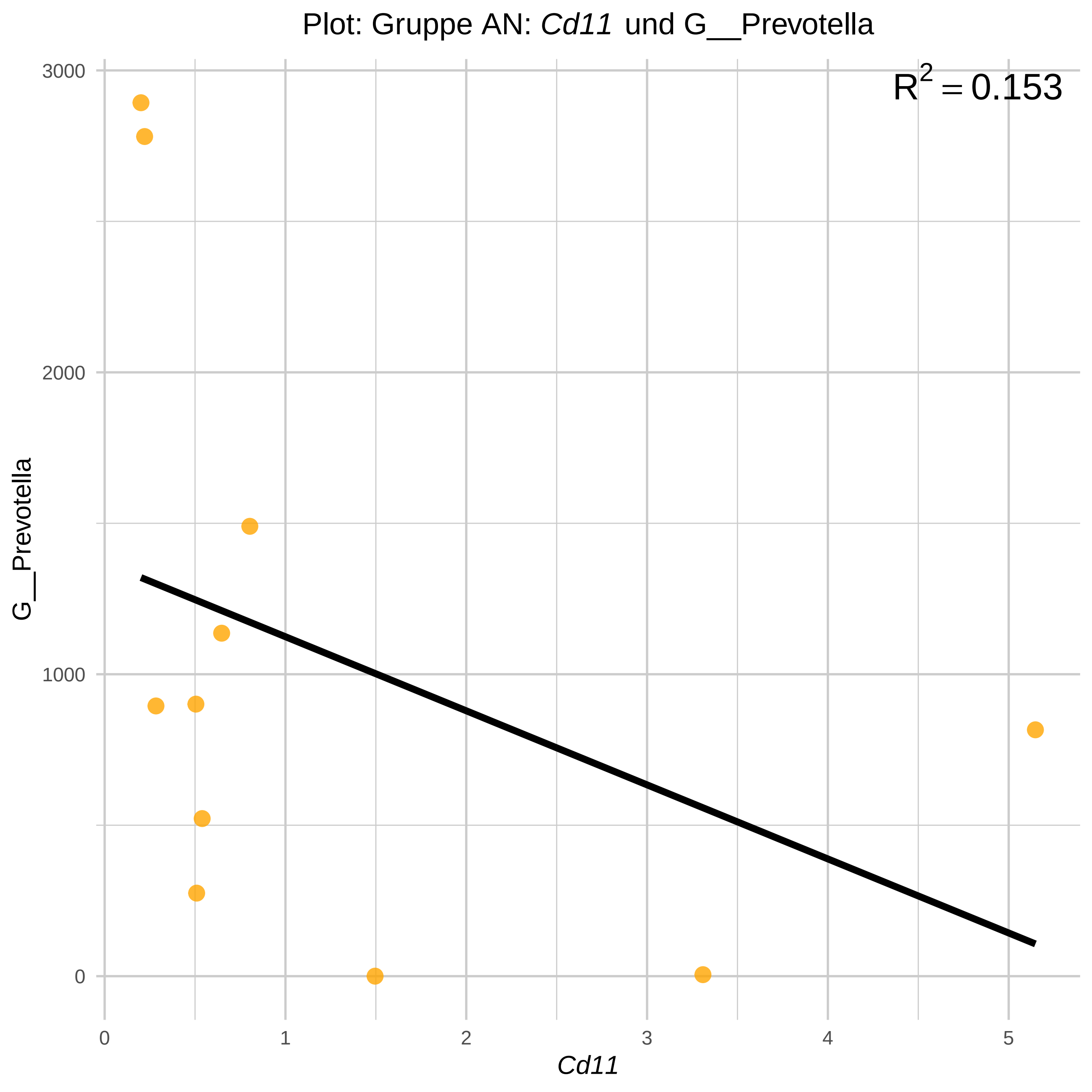

Plot: Gruppe AN: Cd11 und G__Prevotella
R2 = 0.153
3000
2000
1000
0
0
1
2
3
4
5
Cd11
G__Prevotella

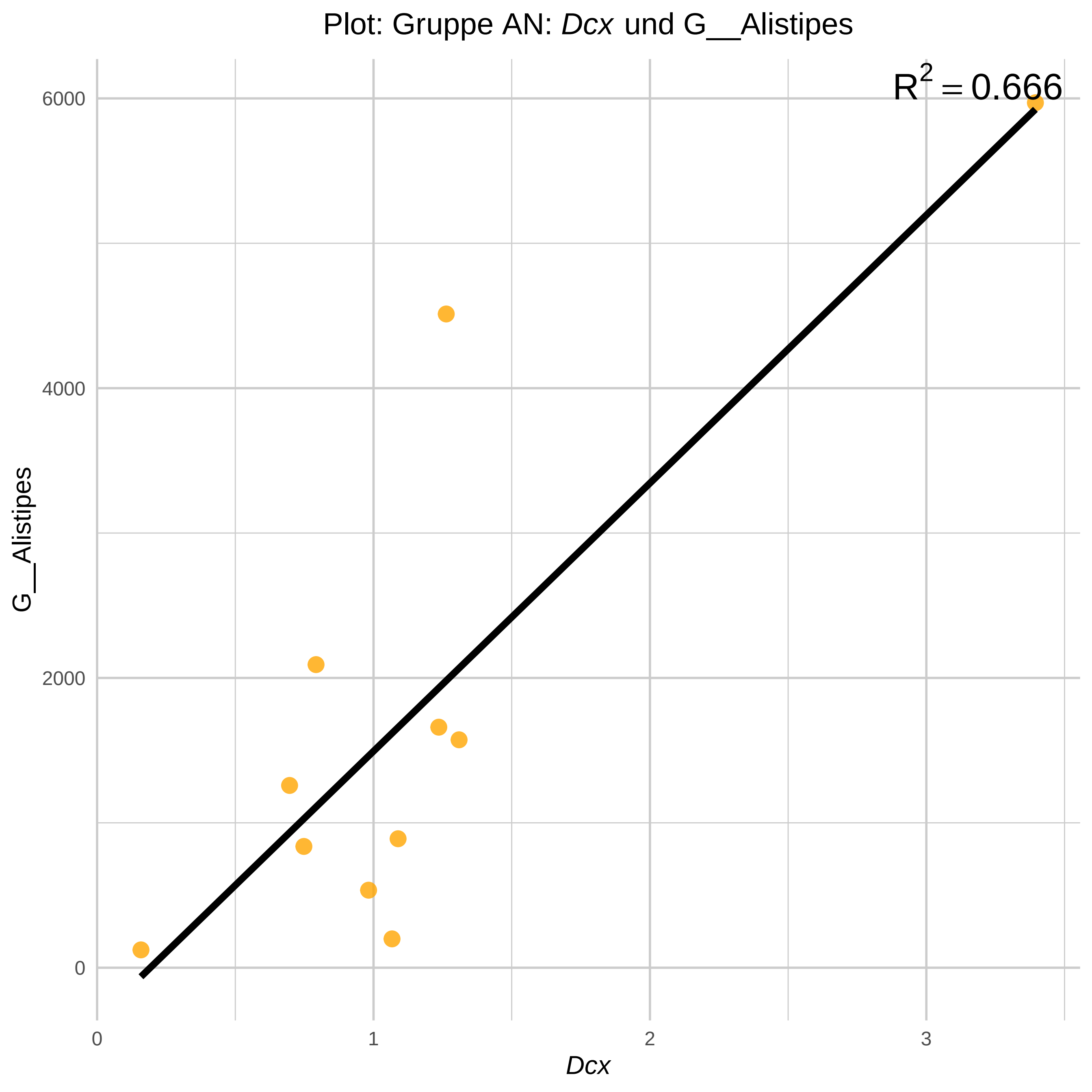

Plot: Gruppe AN: Dcx und G__Alistipes
R2 = 0.666
6000
4000
2000
0
G__Alistipes
0
1
2
3
Dcx

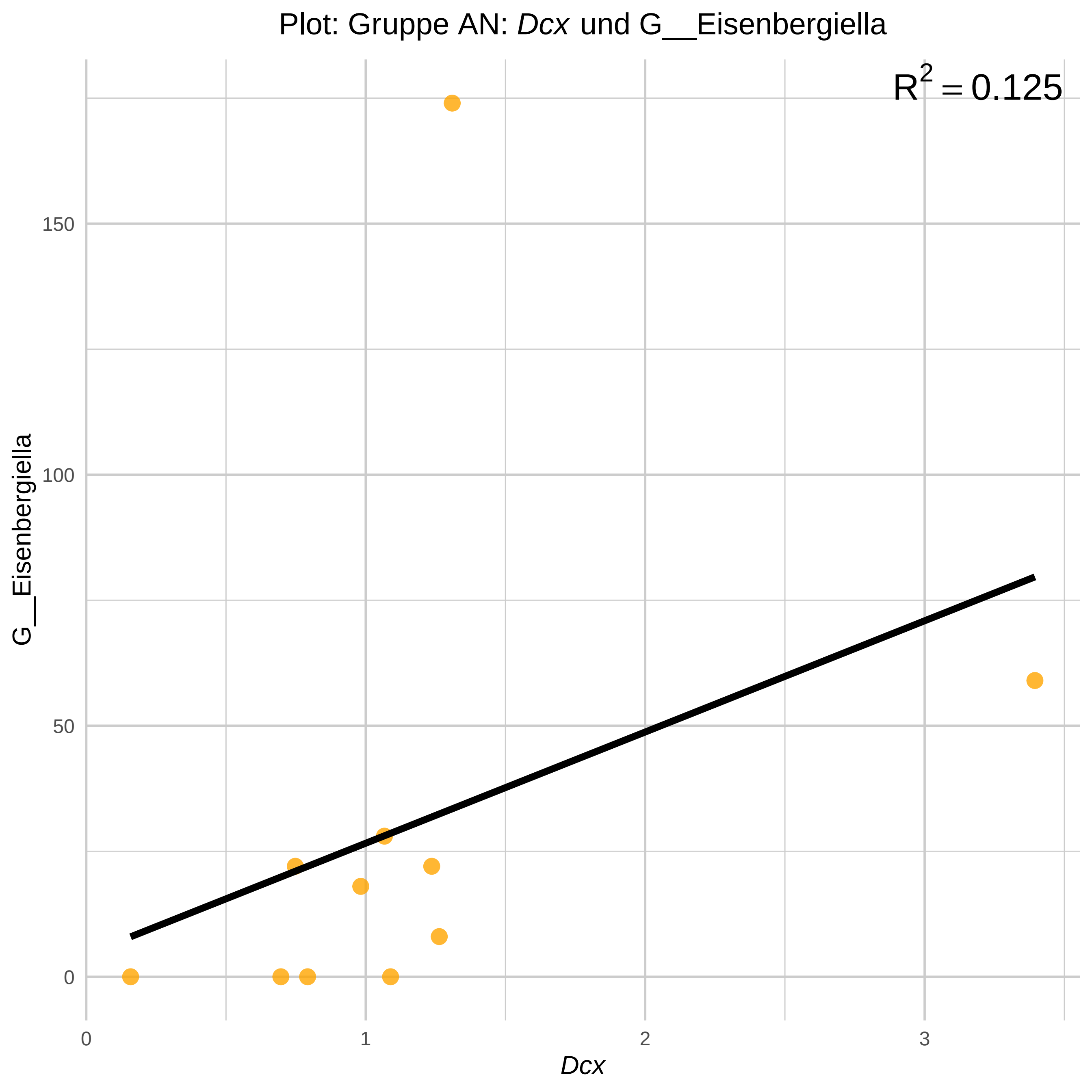

Plot: Gruppe AN: *Dcx* und G__Eisenbergiella
$R^2 = 0.125$
G__Eisenbergiella
150
100
50
0
0
1
2
3
Dcx

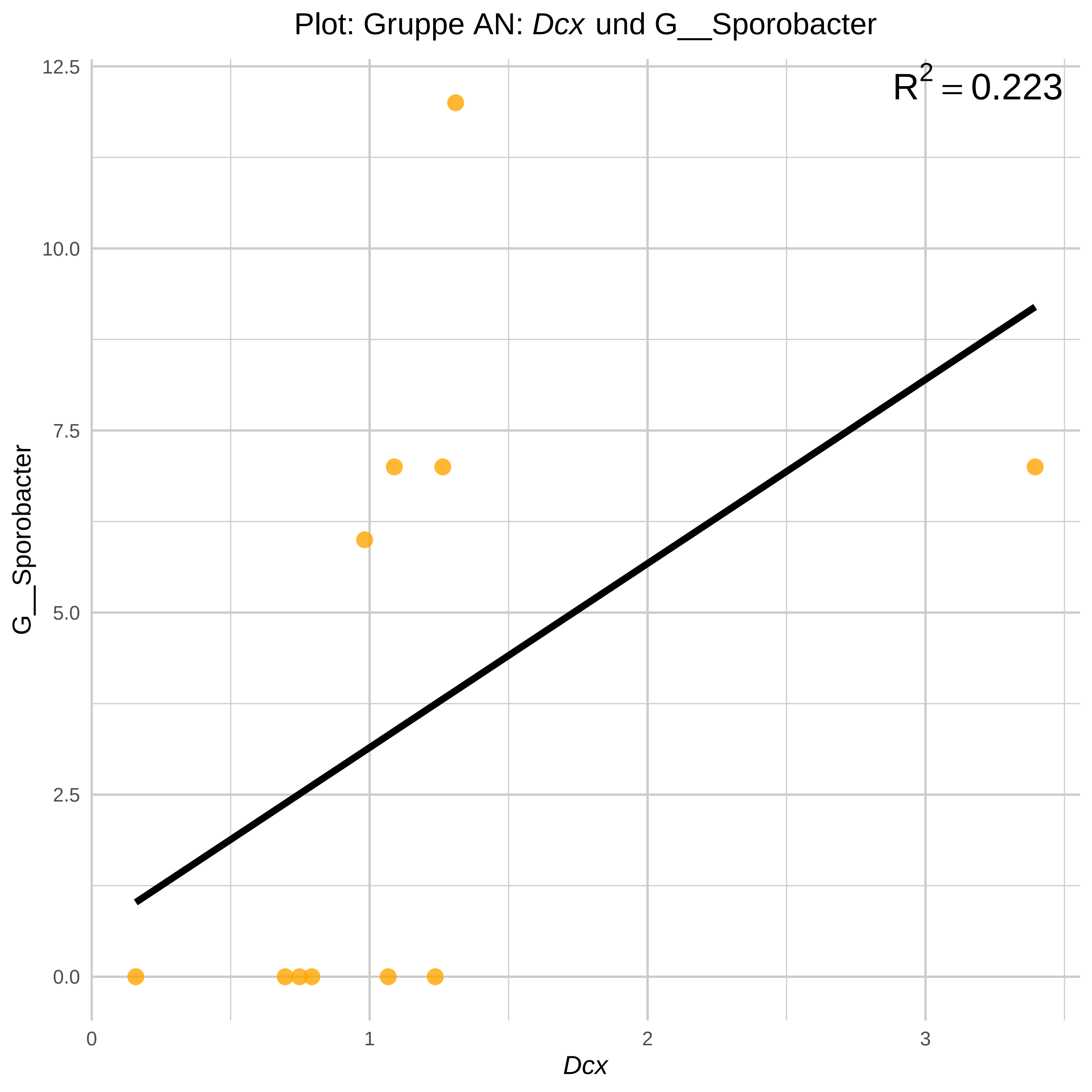

Plot: Gruppe AN: *Dcx* und G__Sporobacter
$R^2 = 0.223$
G__Sporobacter
Dcx
0.0
2.5
5.0
7.5
10.0
12.5
0
1
2
3

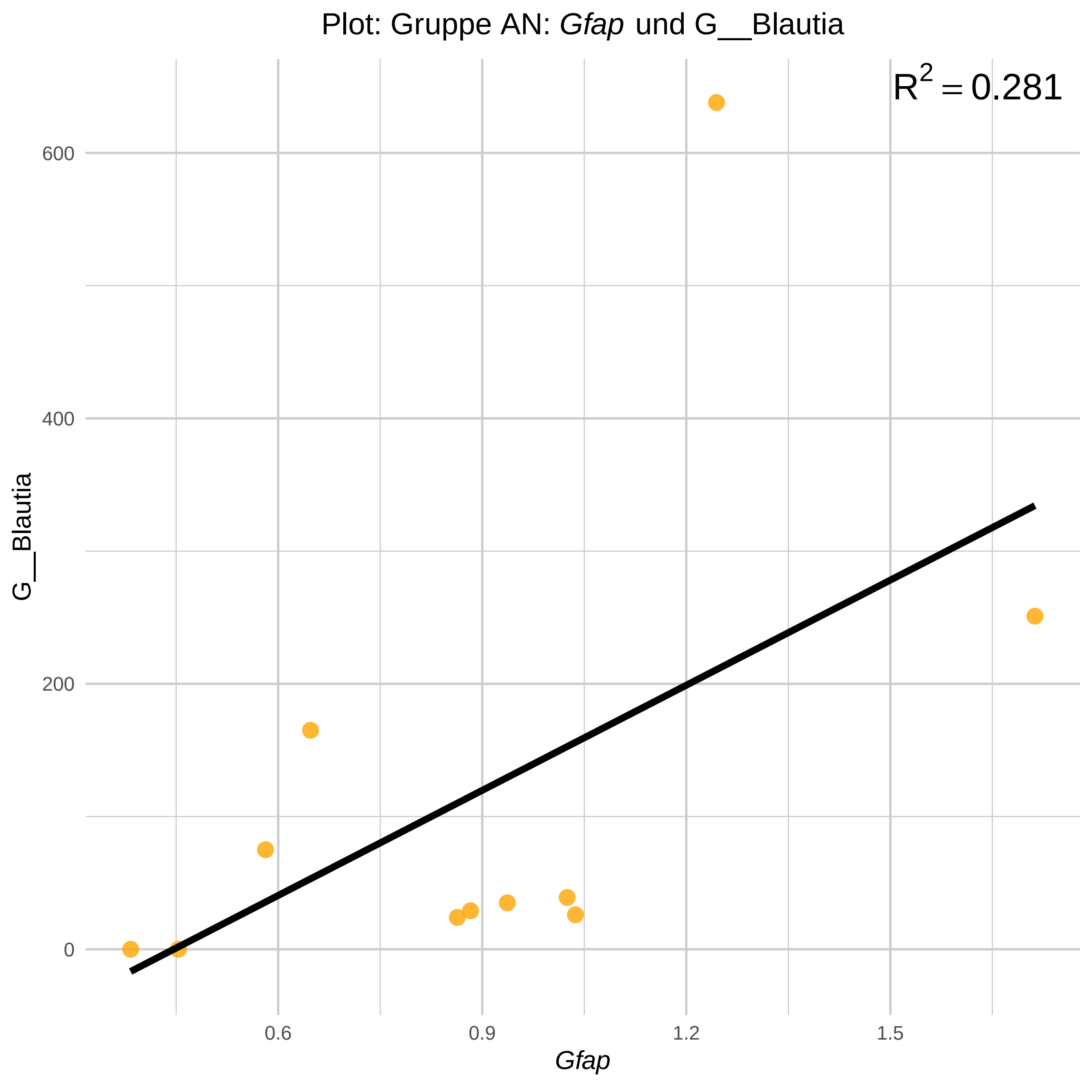
Plot: Gruppe AN: Gfap und G__Blautia
R2 = 0.281
600
400
200
0
G__Blautia
0.6
0.9
1.2
1.5
Gfap

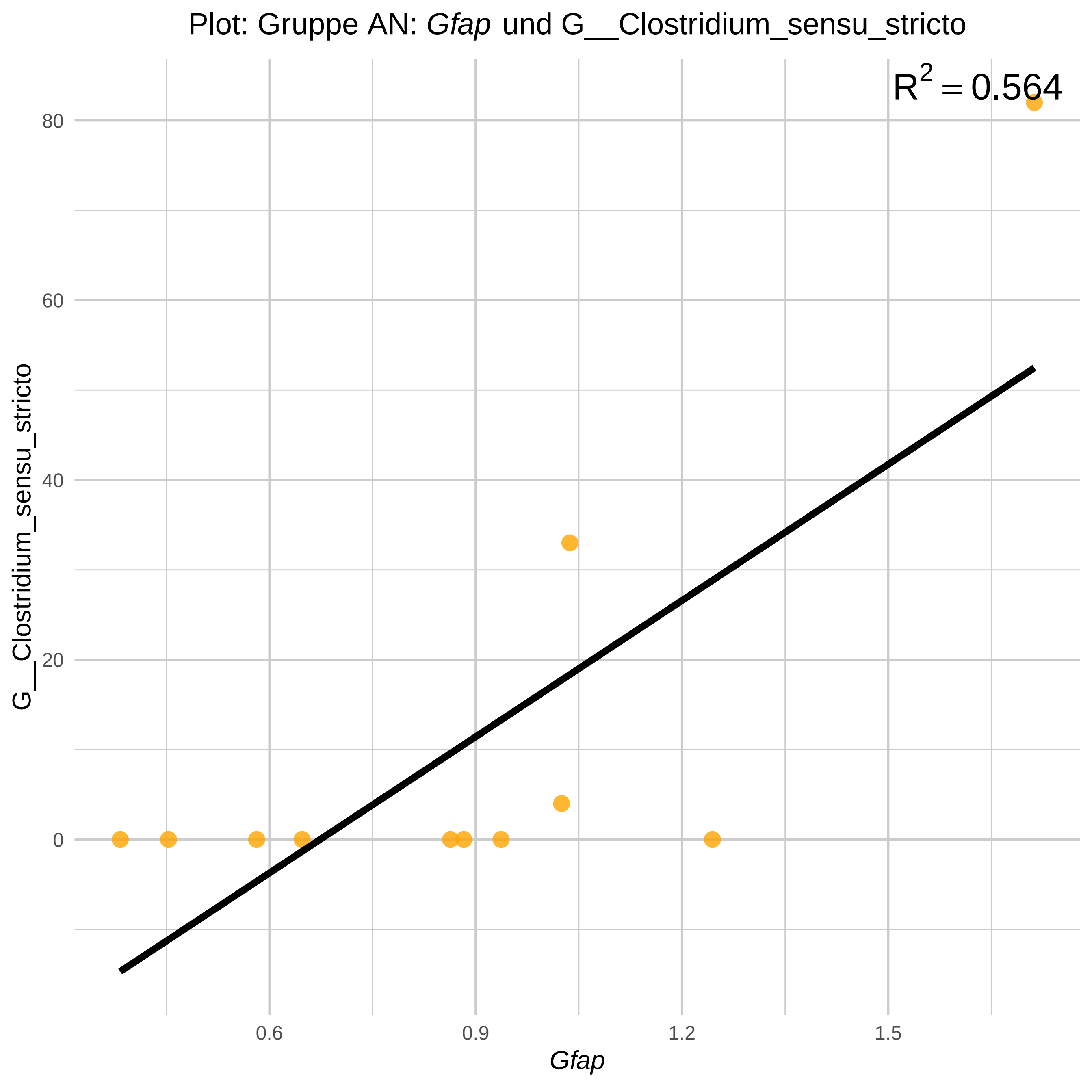

Plot: Gruppe AN: Gfap und G__Clostridium_sensu_stricto
$R^2 = 0.564$
80
60
40
20
0
0.6
0.9
1.2
1.5
Gfap
G__Clostridium_sensu_stricto

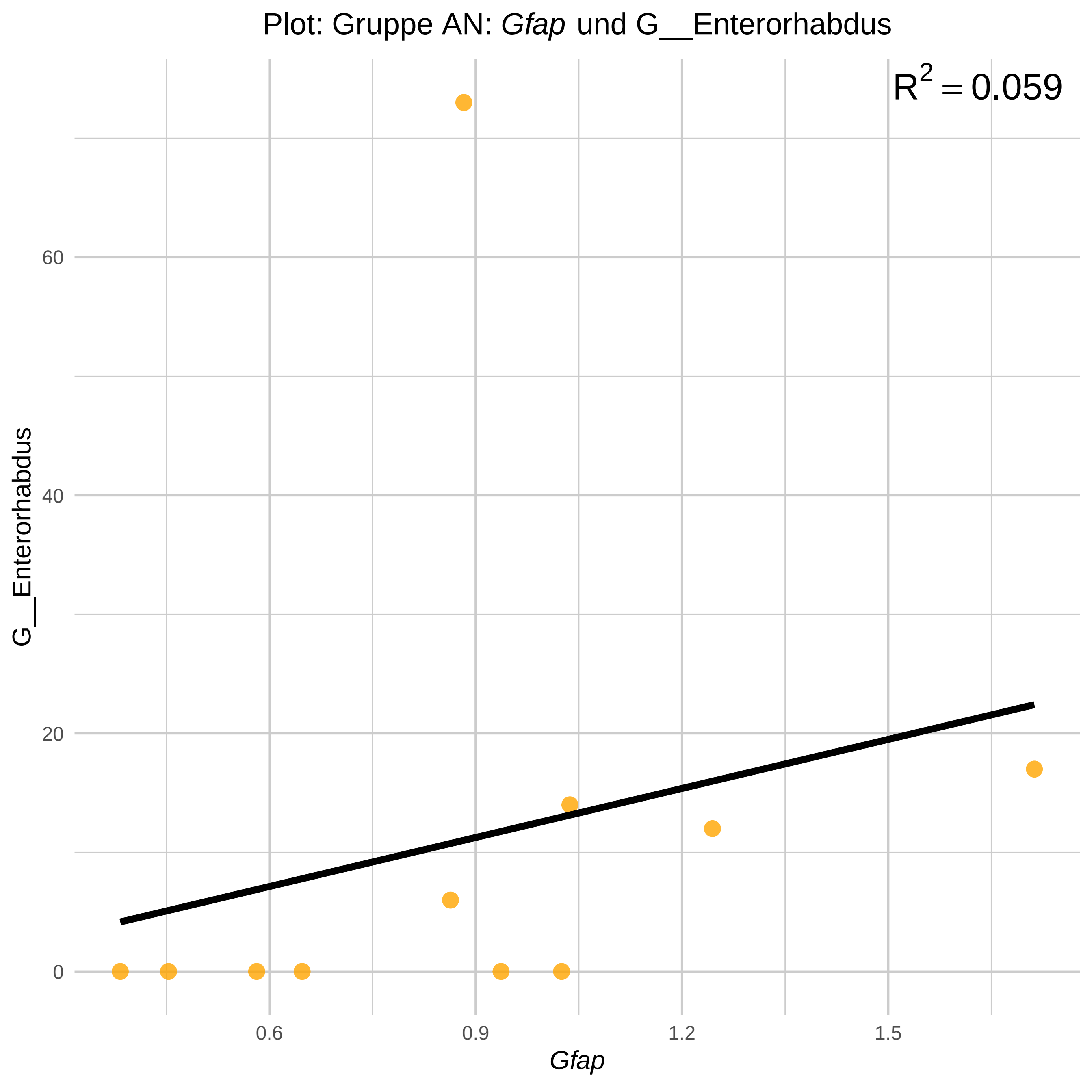
Plot: Gruppe AN: *Gfap* und G__Enterorhabdus
$R^2 = 0.059$
G__Enterorhabdus
60
40
20
0
0.6
0.9
1.2
1.5
Gfap

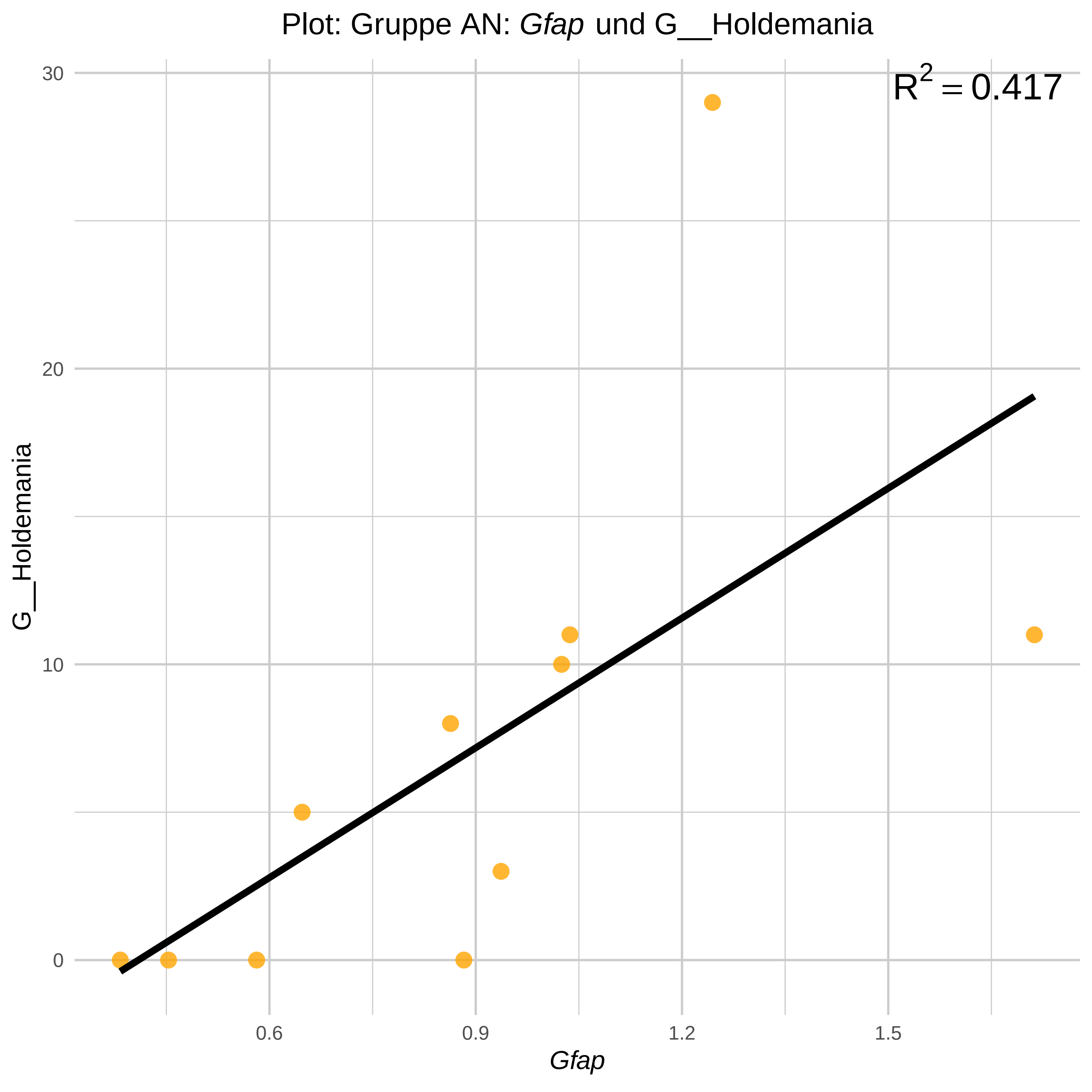
Plot: Gruppe AN: Gfap und G__Holdemania
R2 = 0.417
G__Holdemania
Gfap
0
10
20
30
0.6
0.9
1.2
1.5

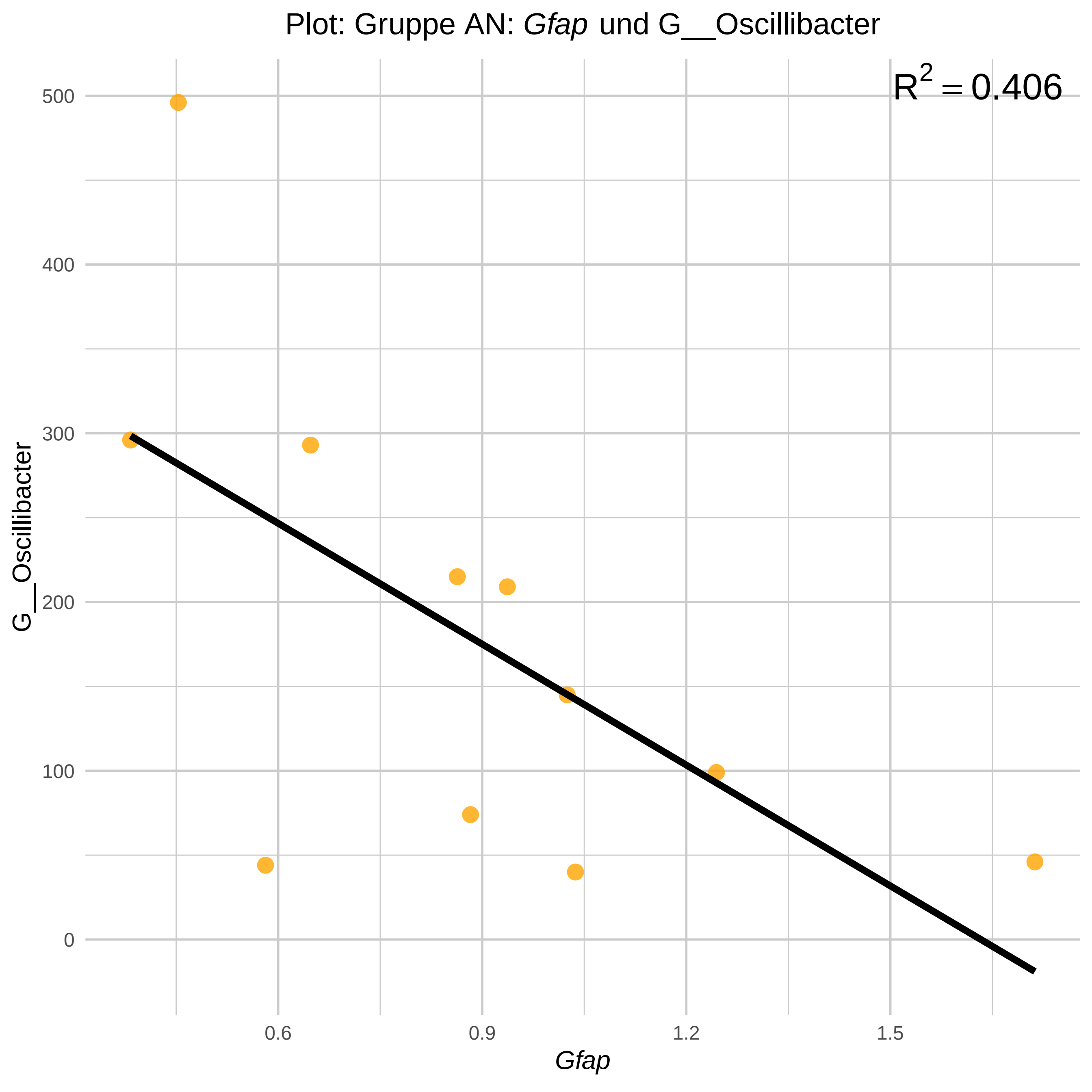

Plot: Gruppe AN: *Gfap* und G__Oscillibacter
$R^2 = 0.406$
500
400
300
200
100
0
0.6
0.9
1.2
1.5
Gfap
G__Oscillibacter

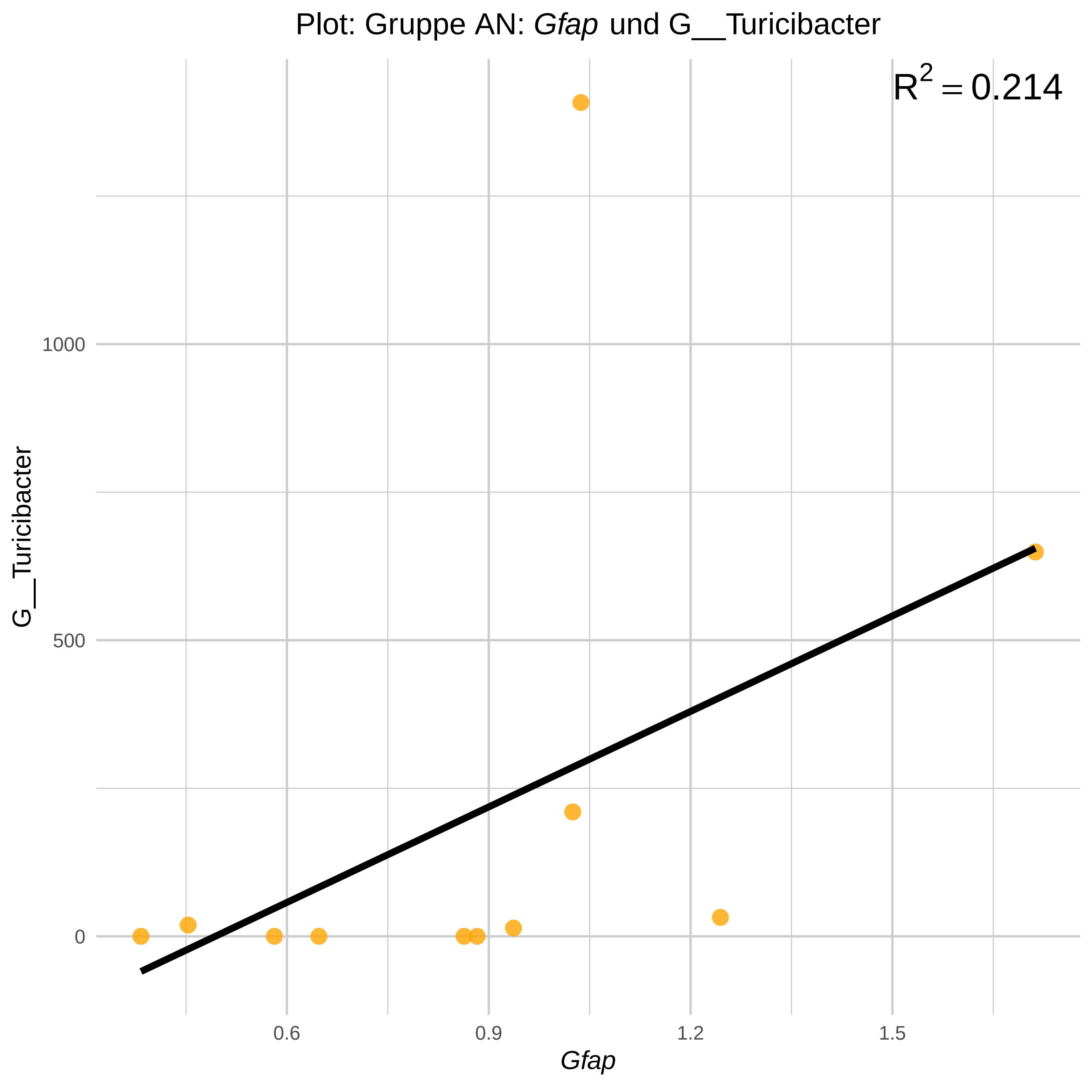

Plot: Gruppe AN: Gfap und G__Turicibacter
$R^2 = 0.214$
G__Turicibacter
1000
500
0
0.6
0.9
1.2
1.5
Gfap

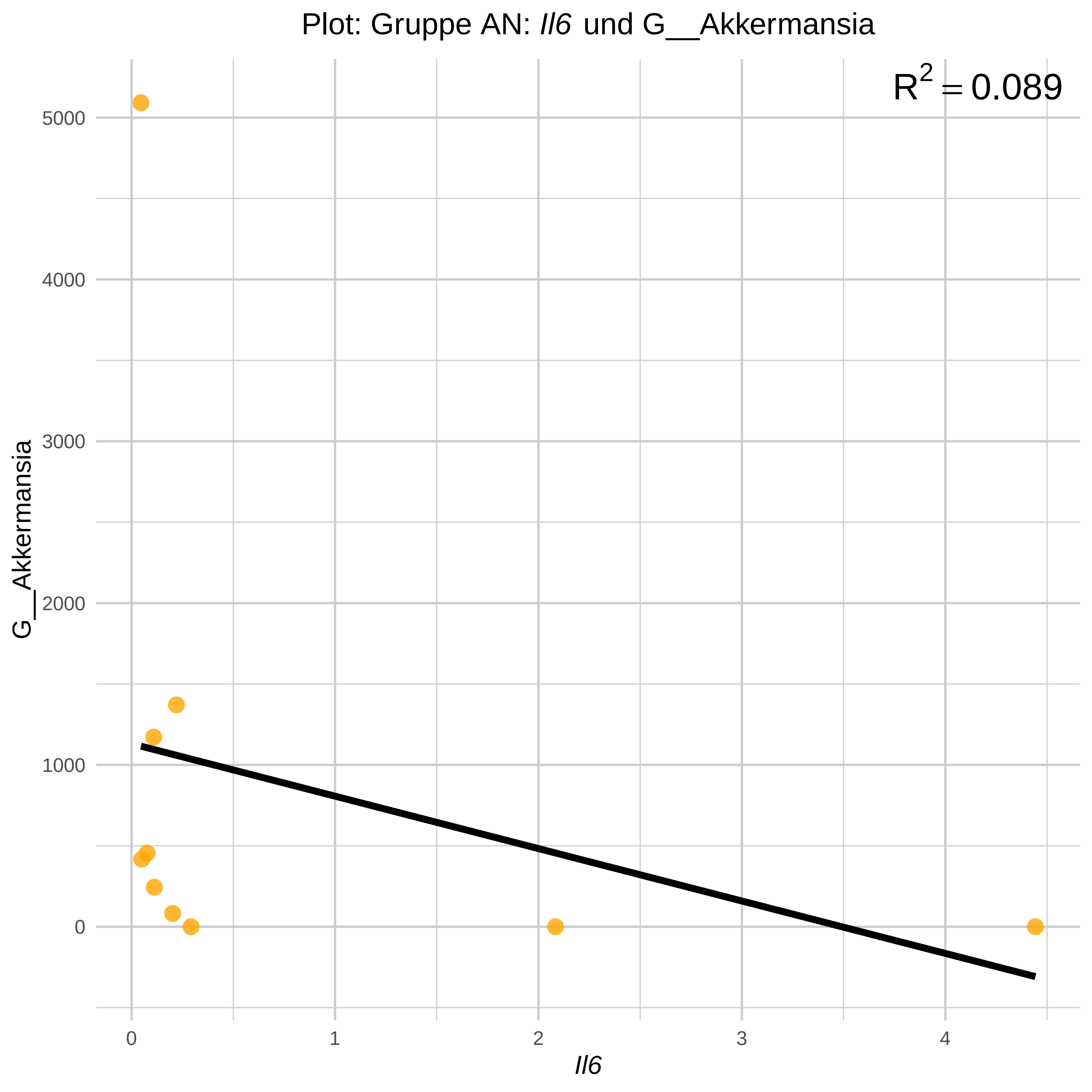

Plot: Gruppe AN: Il6 und G__Akkermansia
R2 = 0.089
G__Akkermansia
5000
4000
3000
2000
1000
0
0
1
2
3
4
Il6

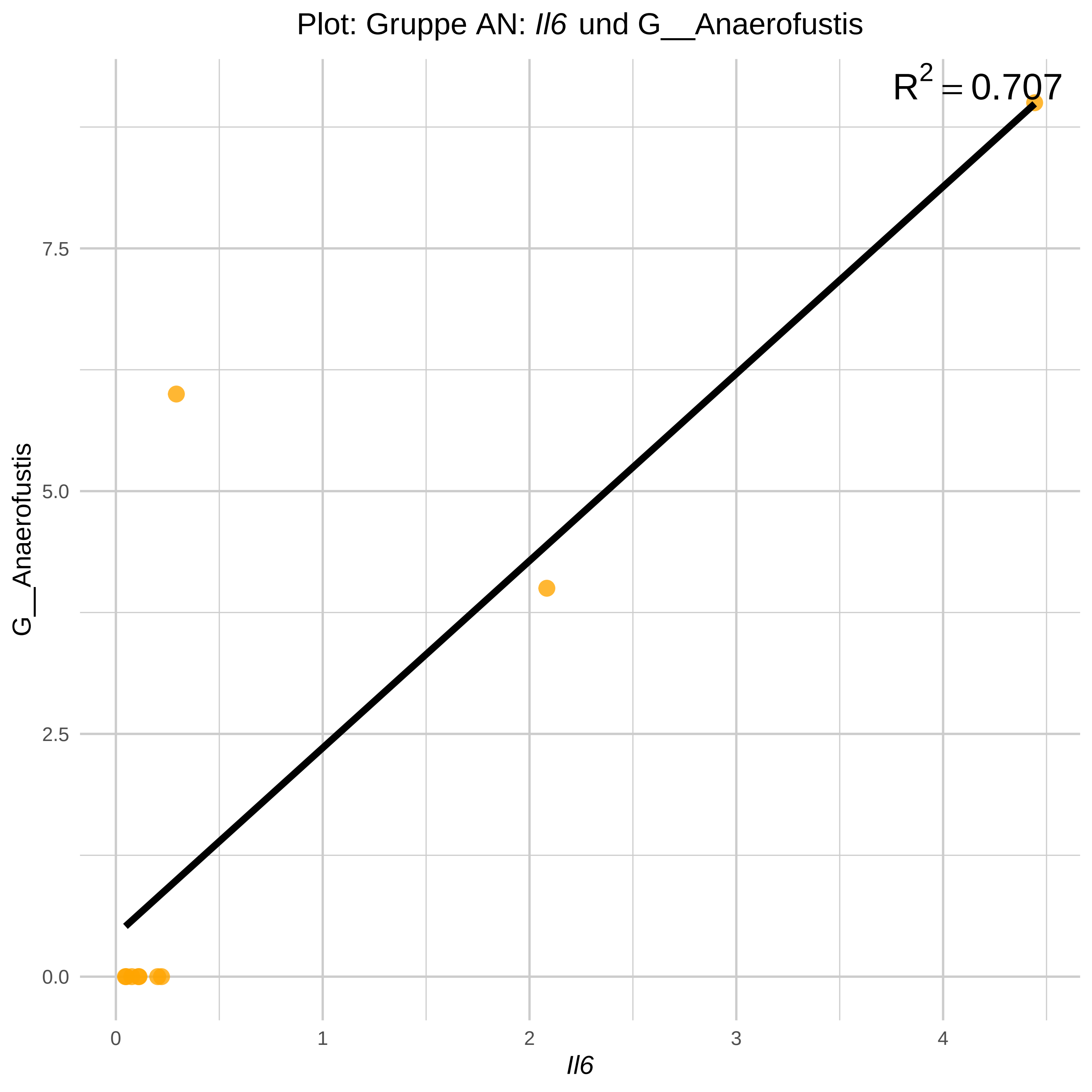
Plot: Gruppe AN: *Il6* und G__Anaerofustis
$R^2 = 0.707$
G__Anaerofustis
7.5
5.0
2.5
0.0
0
1
2
3
4
Il6

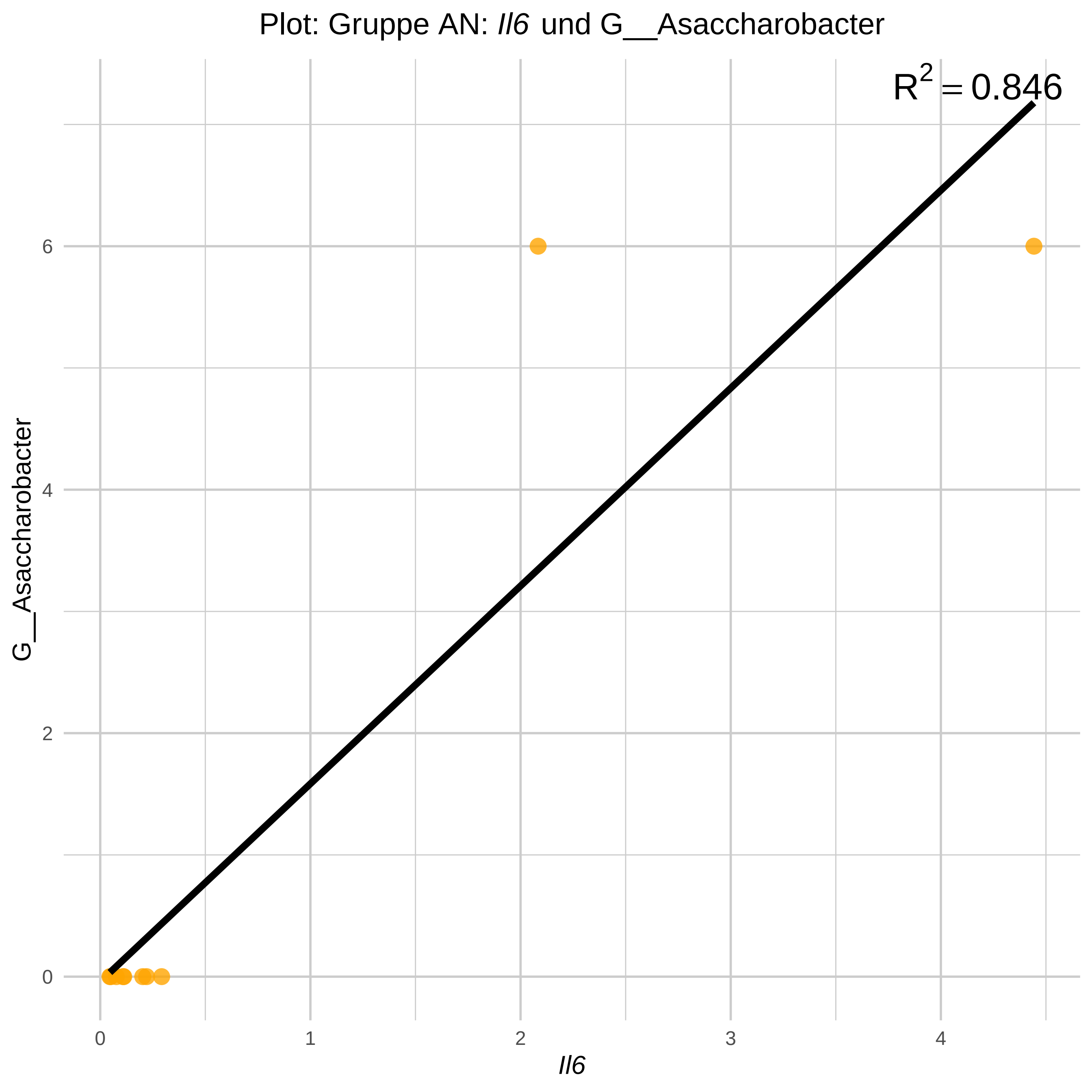
Plot: Gruppe AN: Il6 und G__Asaccharobacter
R2 = 0.846
G__Asaccharobacter
Il6
0
2
4
6
0
1
2
3
4

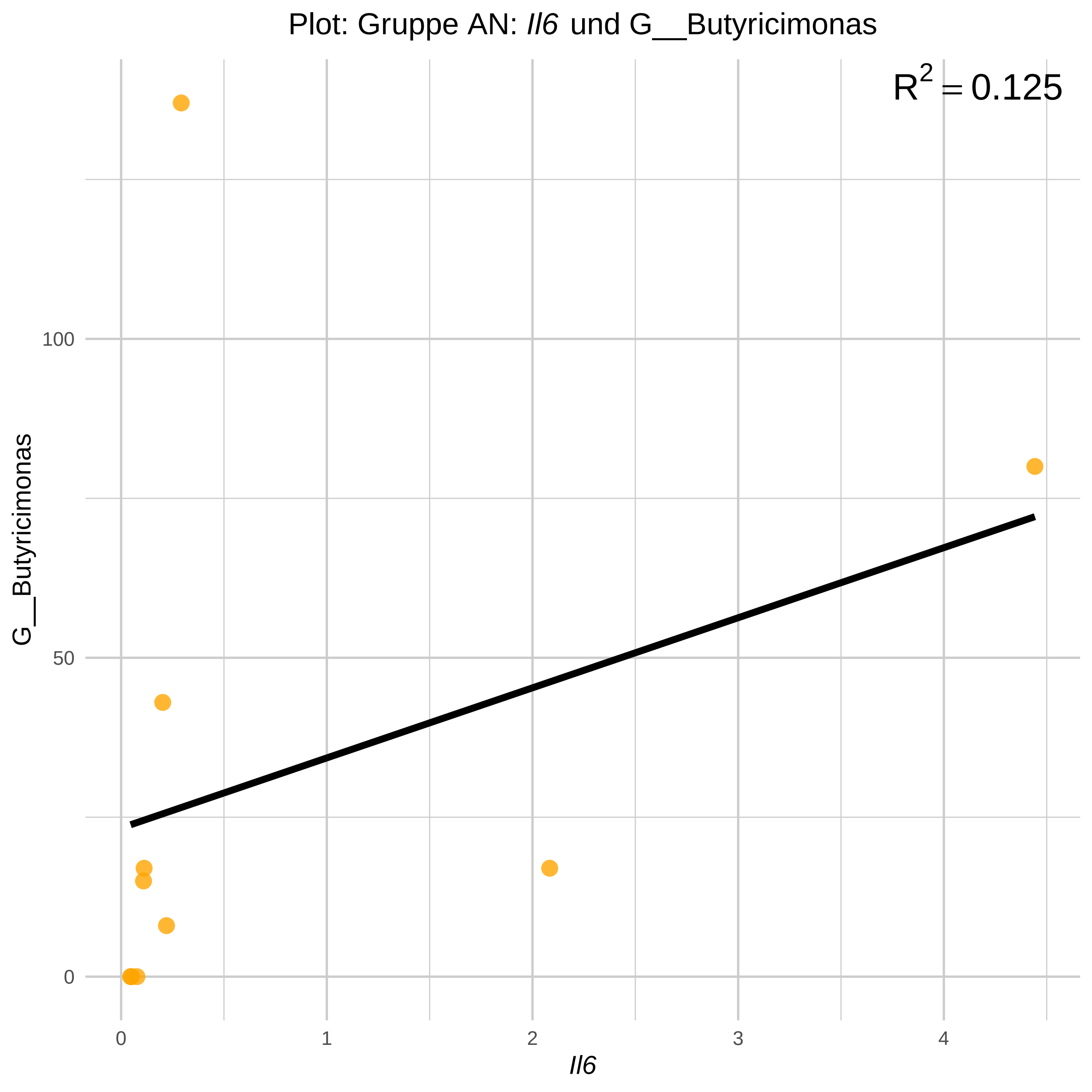

Plot: Gruppe AN: *Il6* und G__Butyricimonas
$R^2 = 0.125$
G__Butyricimonas
100
50
0
0
1
2
3
4
Il6

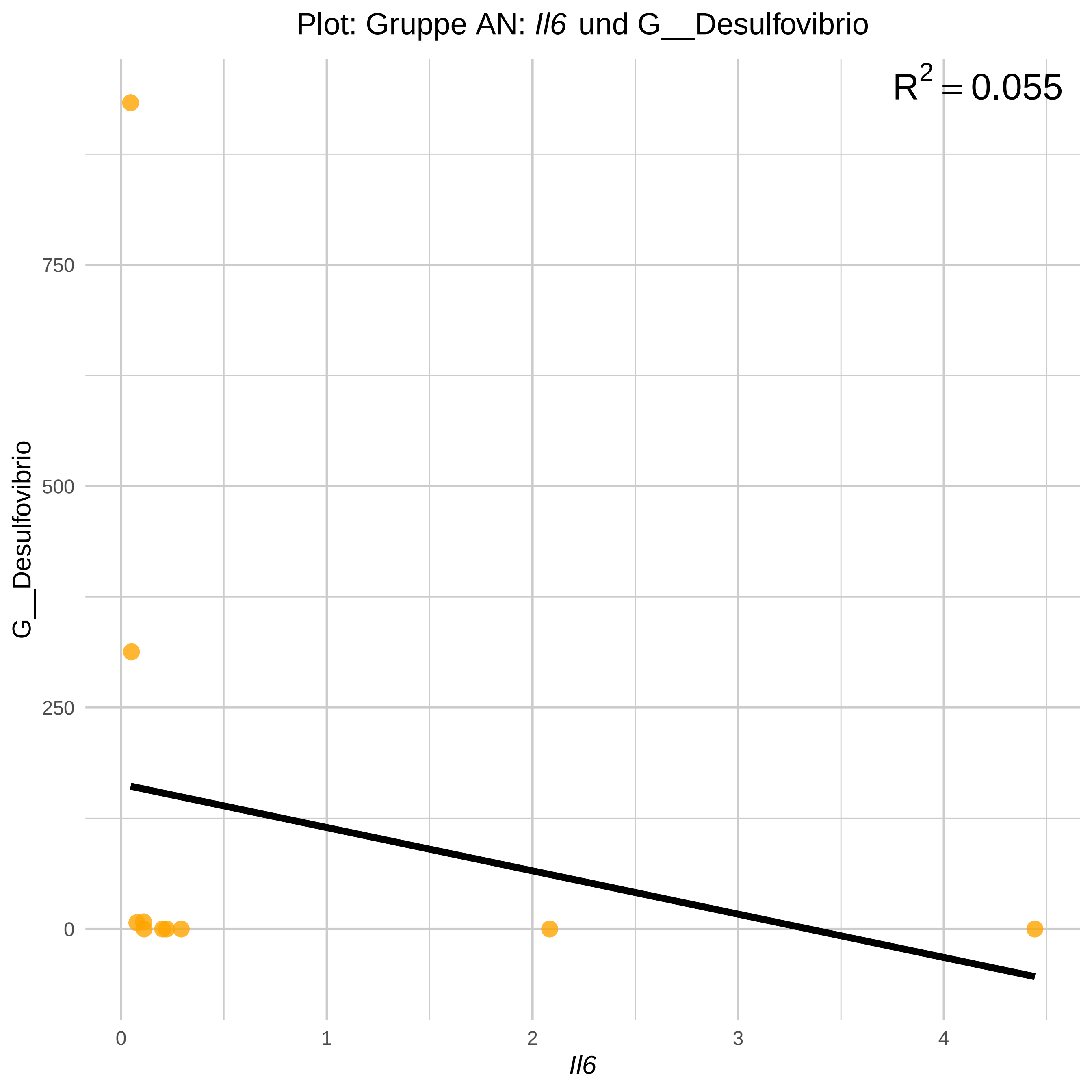

Plot: Gruppe AN: Il6 und G__Desulfovibrio
R2 = 0.055
750
500
250
0
G__Desulfovibrio
0
1
2
3
4
Il6

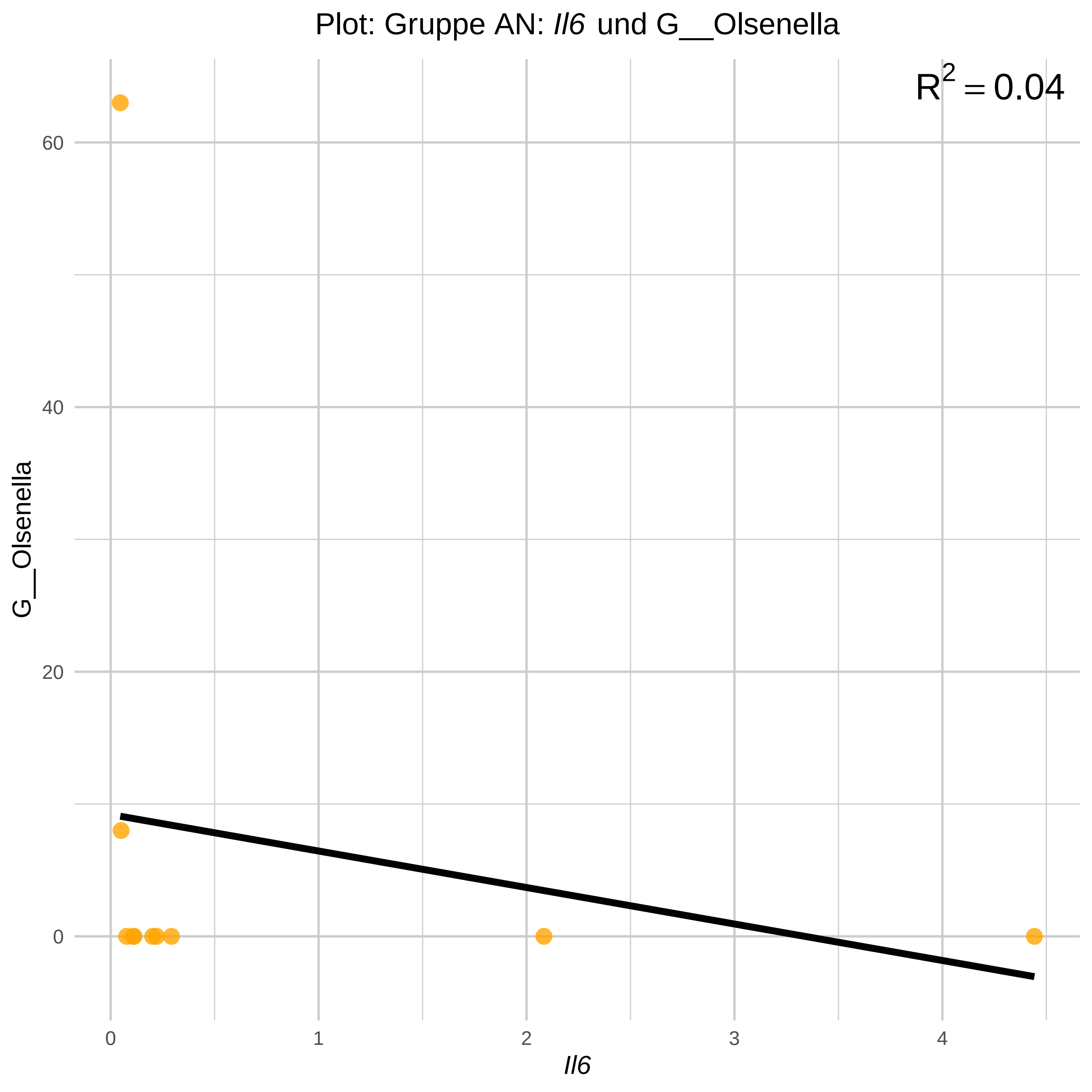

Plot: Gruppe AN: *Il6* und G__Olsenella
$R^2 = 0.04$
60
40
20
0
G__Olsenella
0
1
2
3
4
Il6

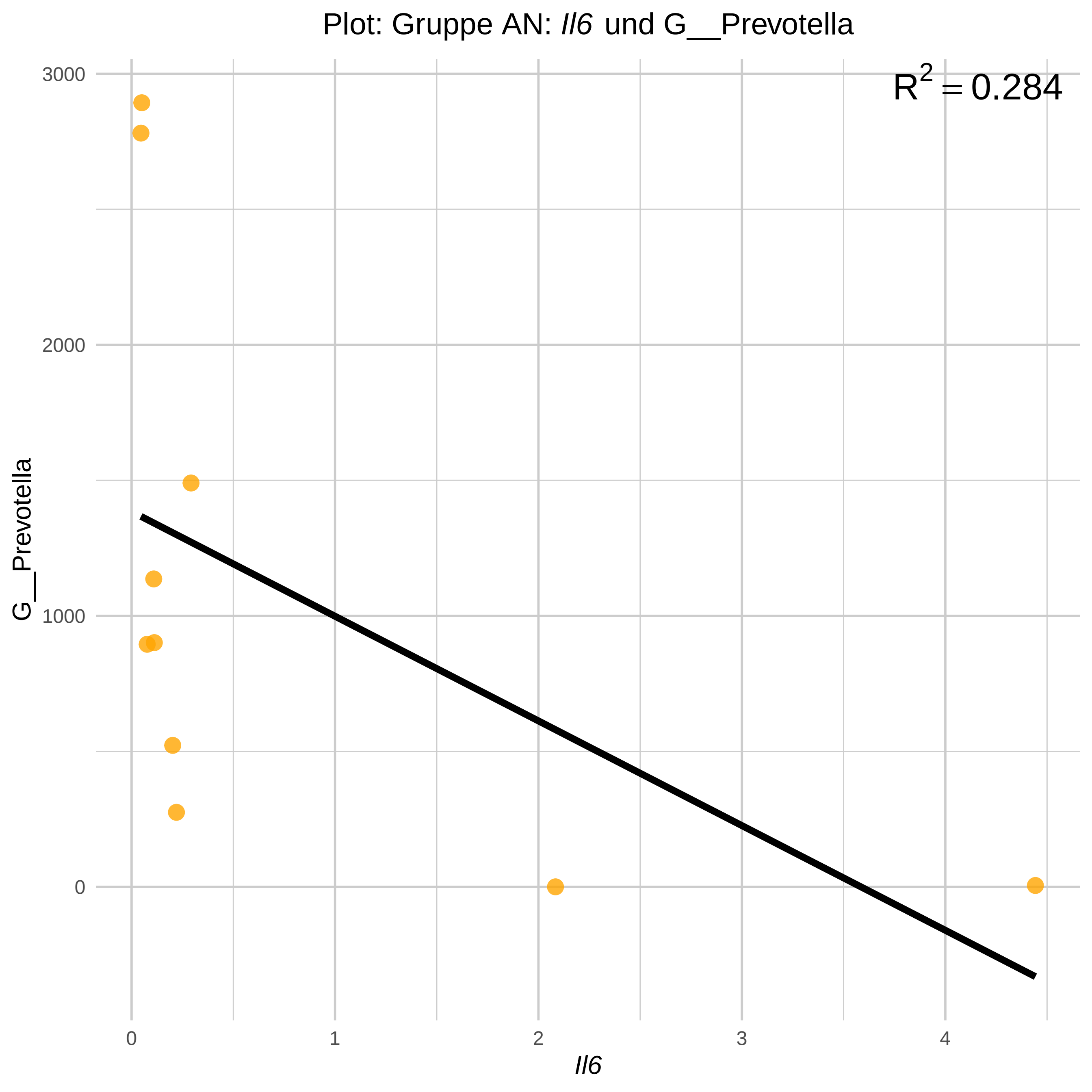

Plot: Gruppe AN: Il6 und G__Prevotella
R2 = 0.284
3000
2000
1000
0
G__Prevotella
0
1
2
3
4
Il6

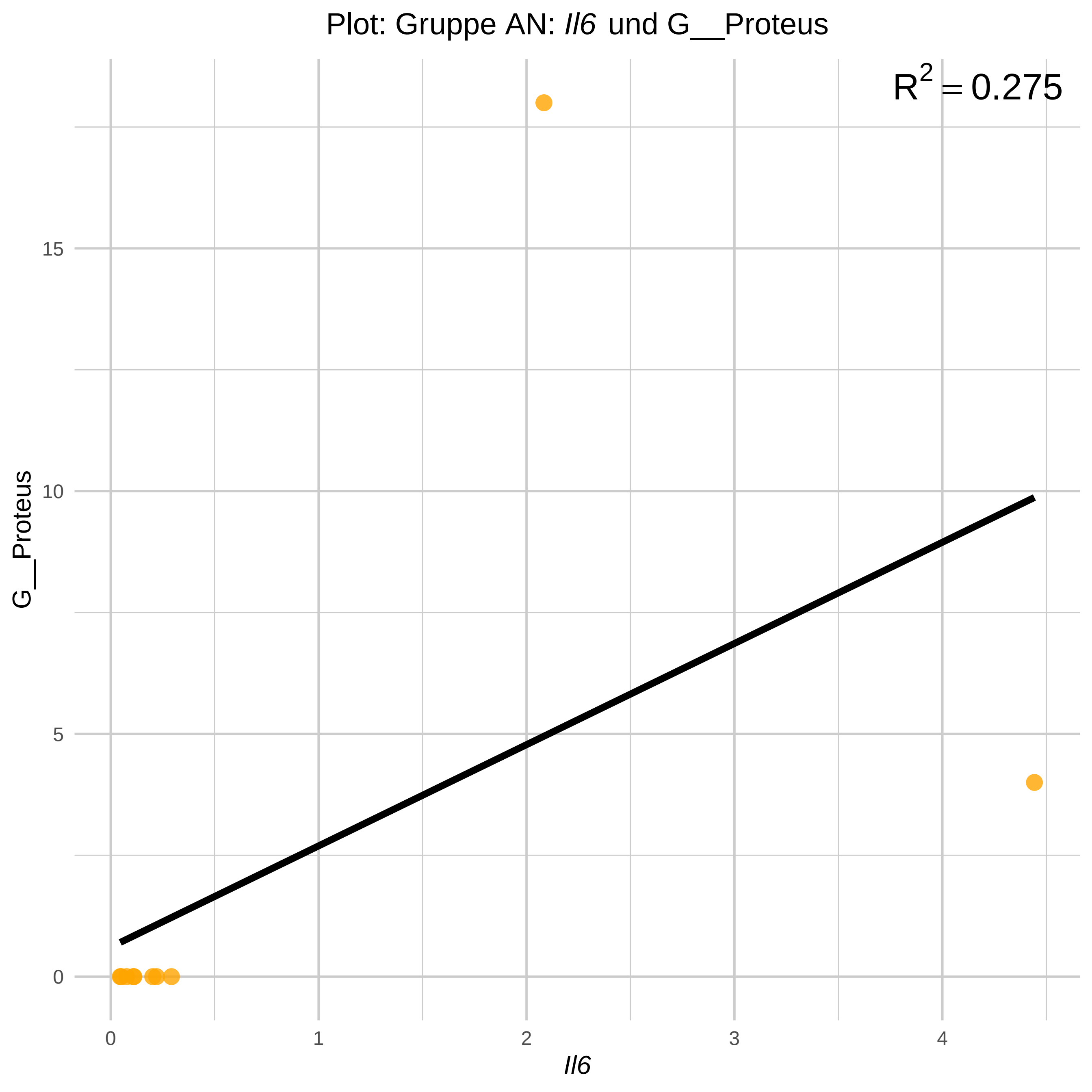

Plot: Gruppe AN: *Il6* und G__Proteus
$R^2 = 0.275$
G__Proteus
Il6
0
5
10
15
0
1
2
3
4

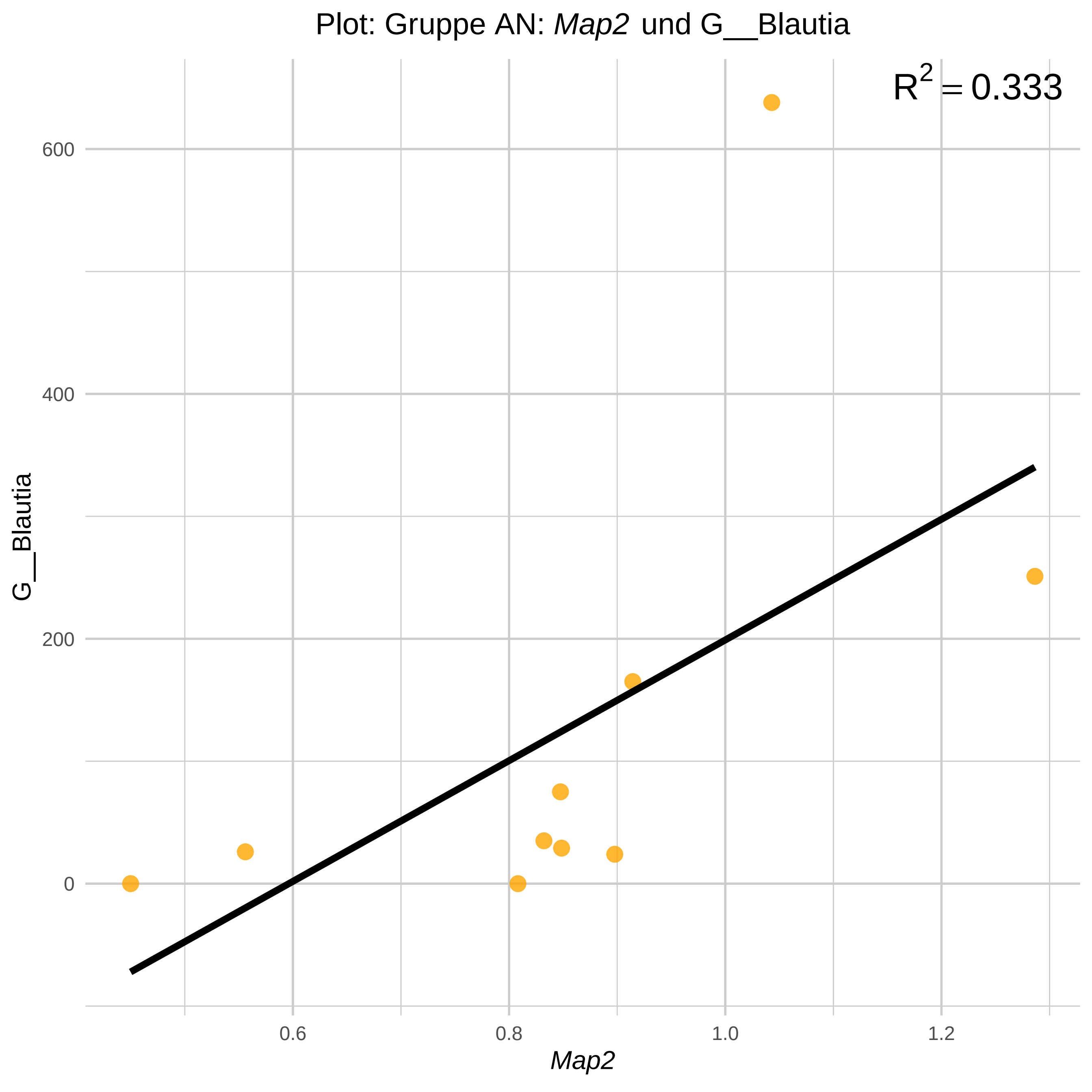

Plot: Gruppe AN: Map2 und G__Blautia
R2 = 0.333
600
400
200
0
G__Blautia
0.6
0.8
1.0
1.2
Map2

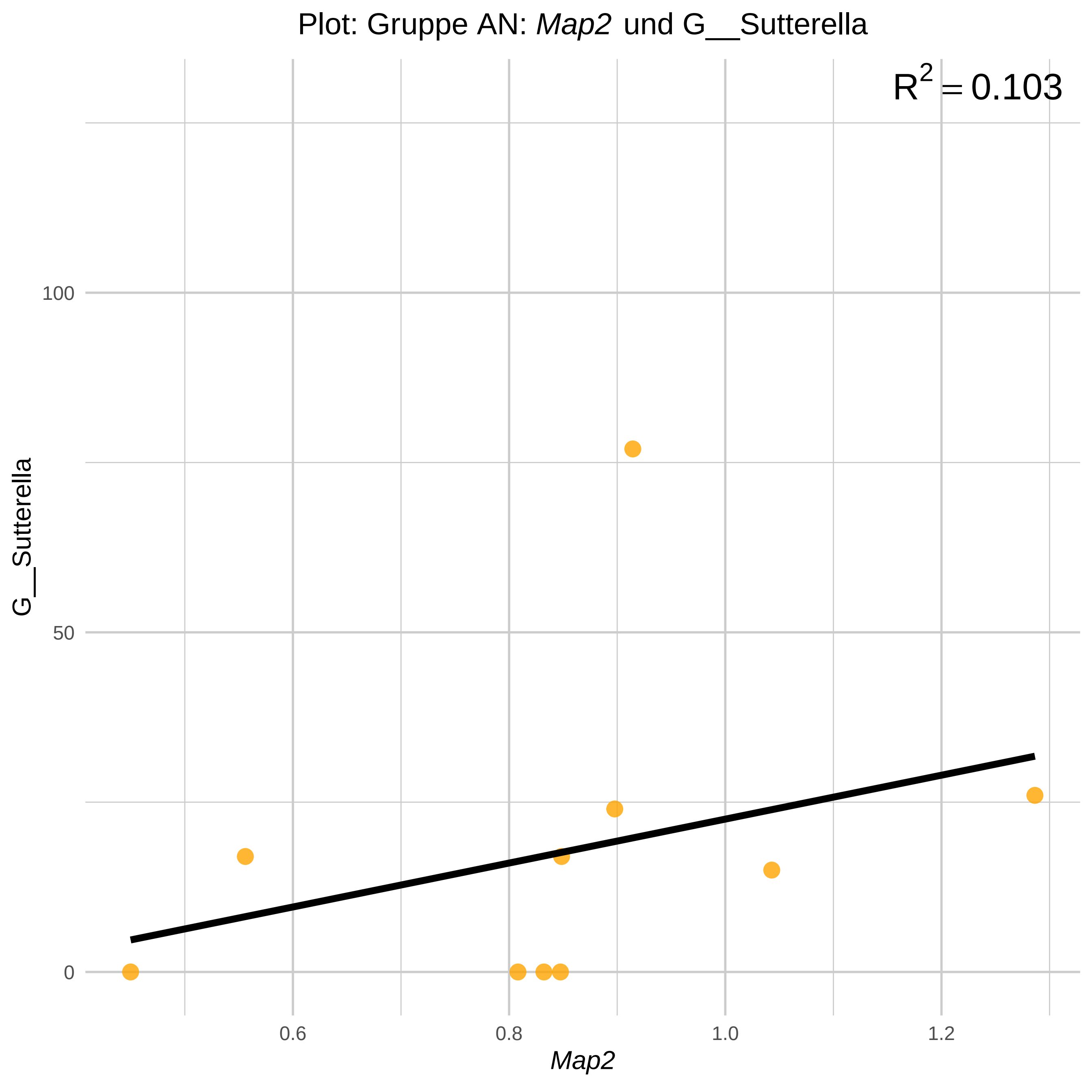
Plot: Gruppe AN: Map2 und G__Sutterella
$R^2 = 0.103$
G__Sutterella
100
50
0
0.6
0.8
1.0
1.2
Map2

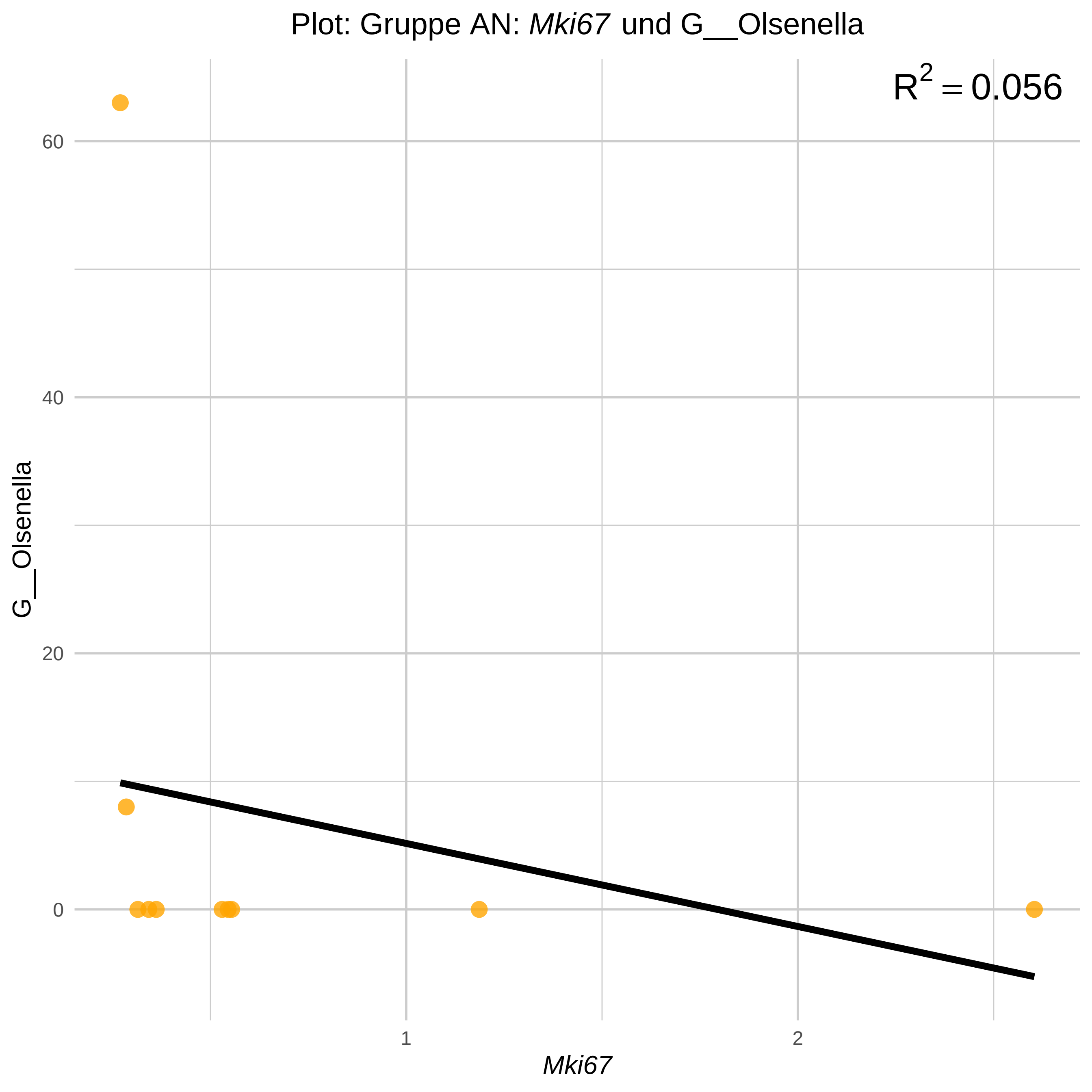

Plot: Gruppe AN: Mki67 und G__Olsenella
R2 = 0.056
60
40
20
0
G__Olsenella
1
2
Mki67

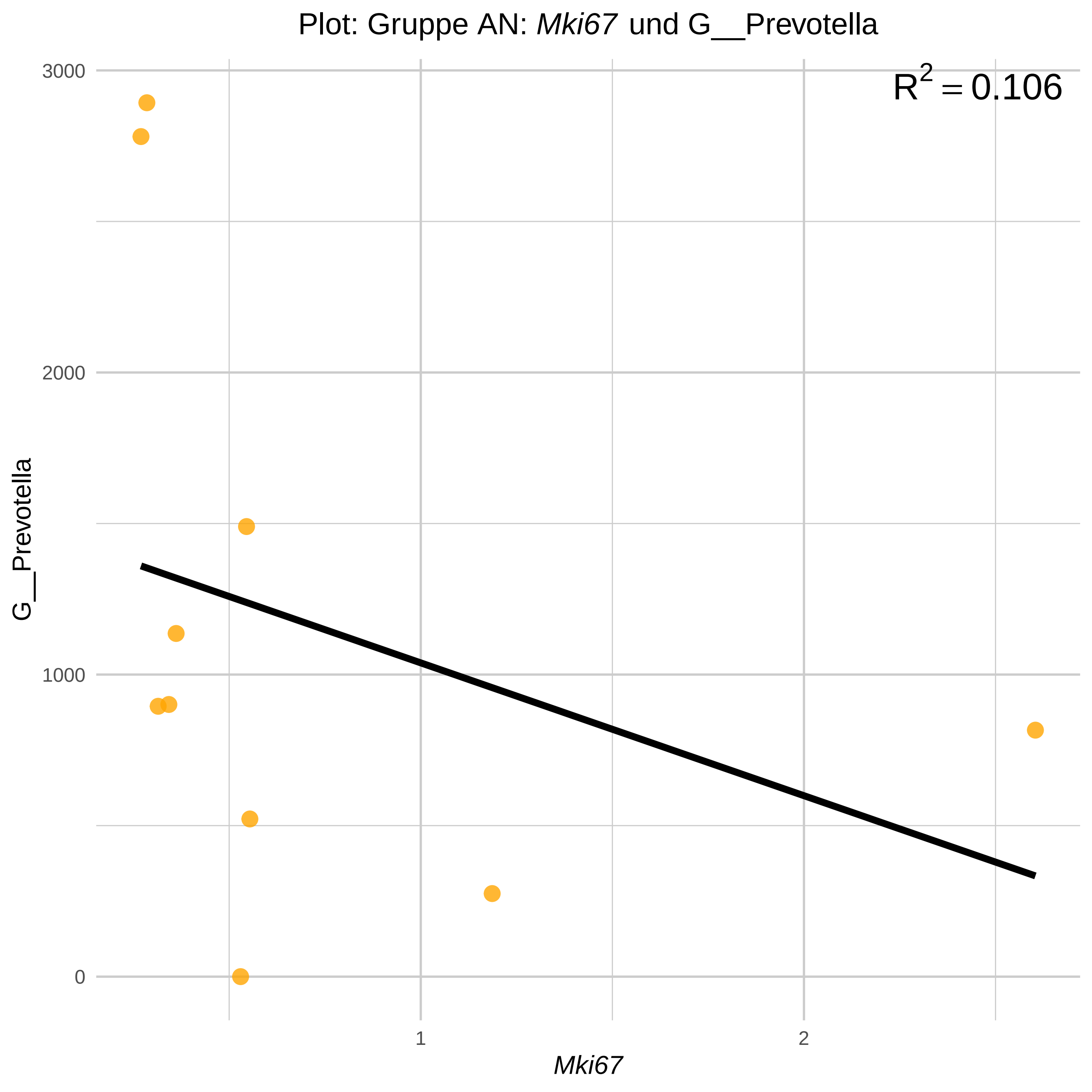

Plot: Gruppe AN: Mki67 und G__Prevotella
R2 = 0.106
3000
2000
1000
0
G__Prevotella
1
2
Mki67

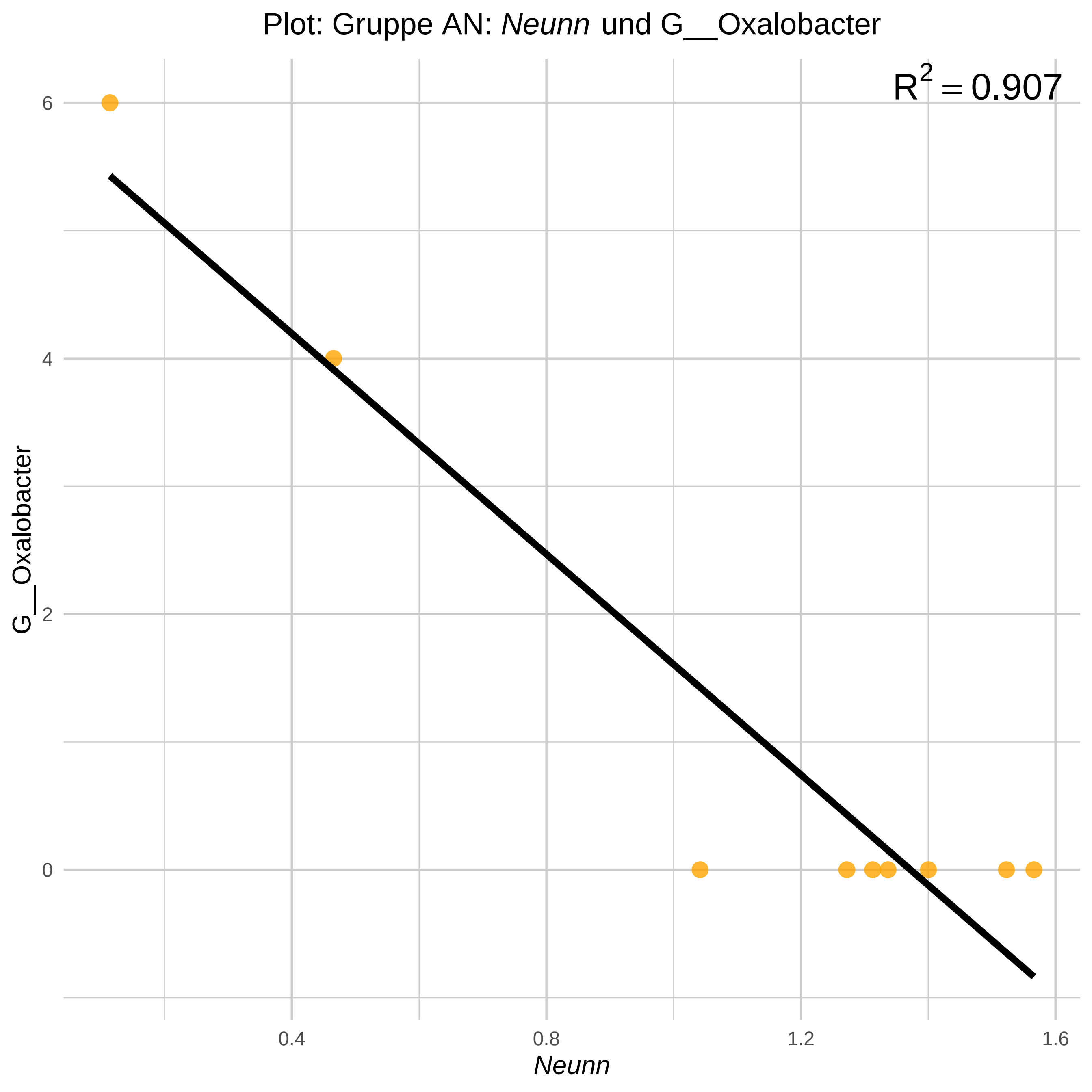

Plot: Gruppe AN: *Neunn* und G__Oxalobacter
$R^2 = 0.907$
G__Oxalobacter
6
4
2
0
0.4
0.8
1.2
1.6
Neunn

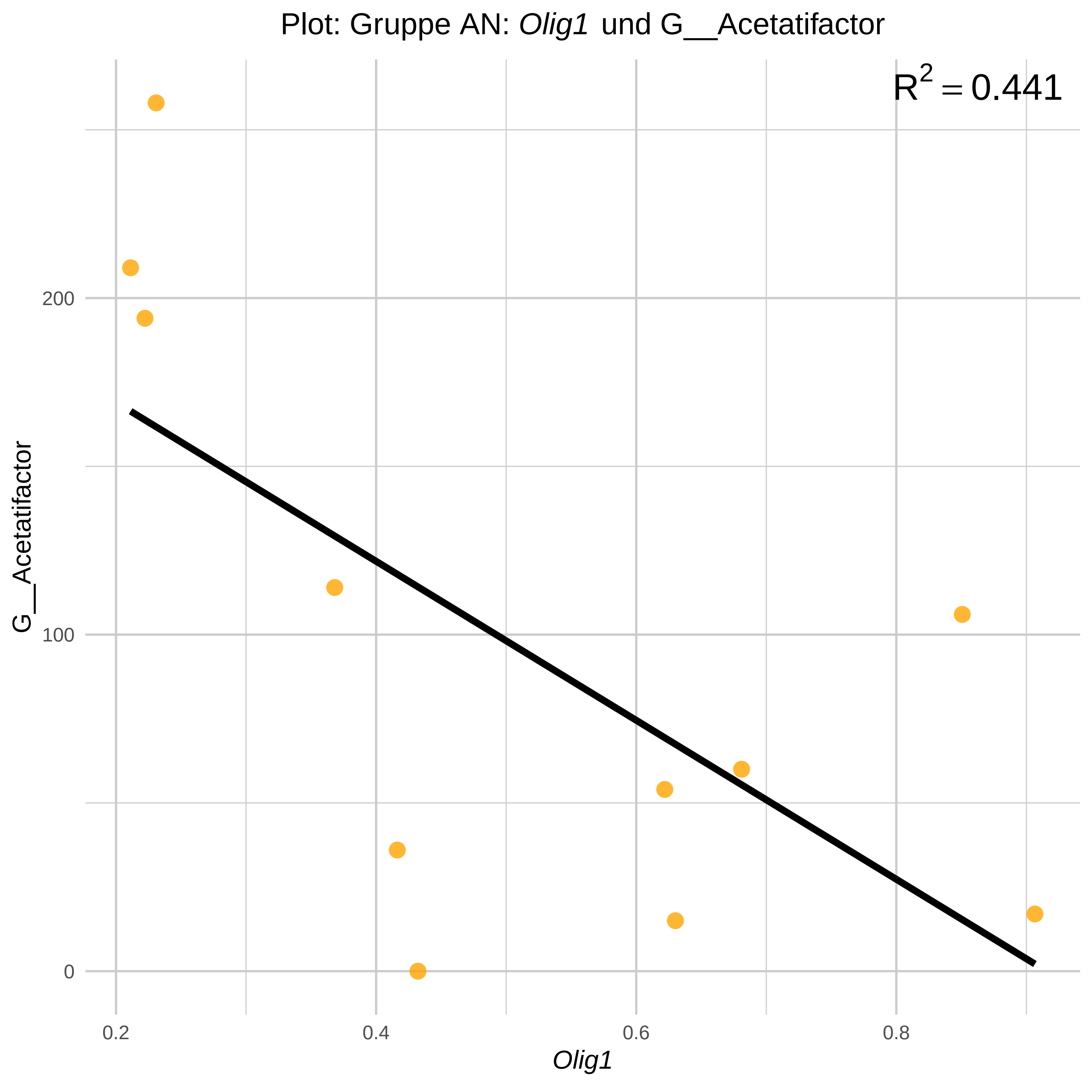

Plot: Gruppe AN: Olig1 und G__Acetatifactor
$R^2 = 0.441$
G__Acetatifactor
200
100
0
0.2
0.4
0.6
0.8
Olig1

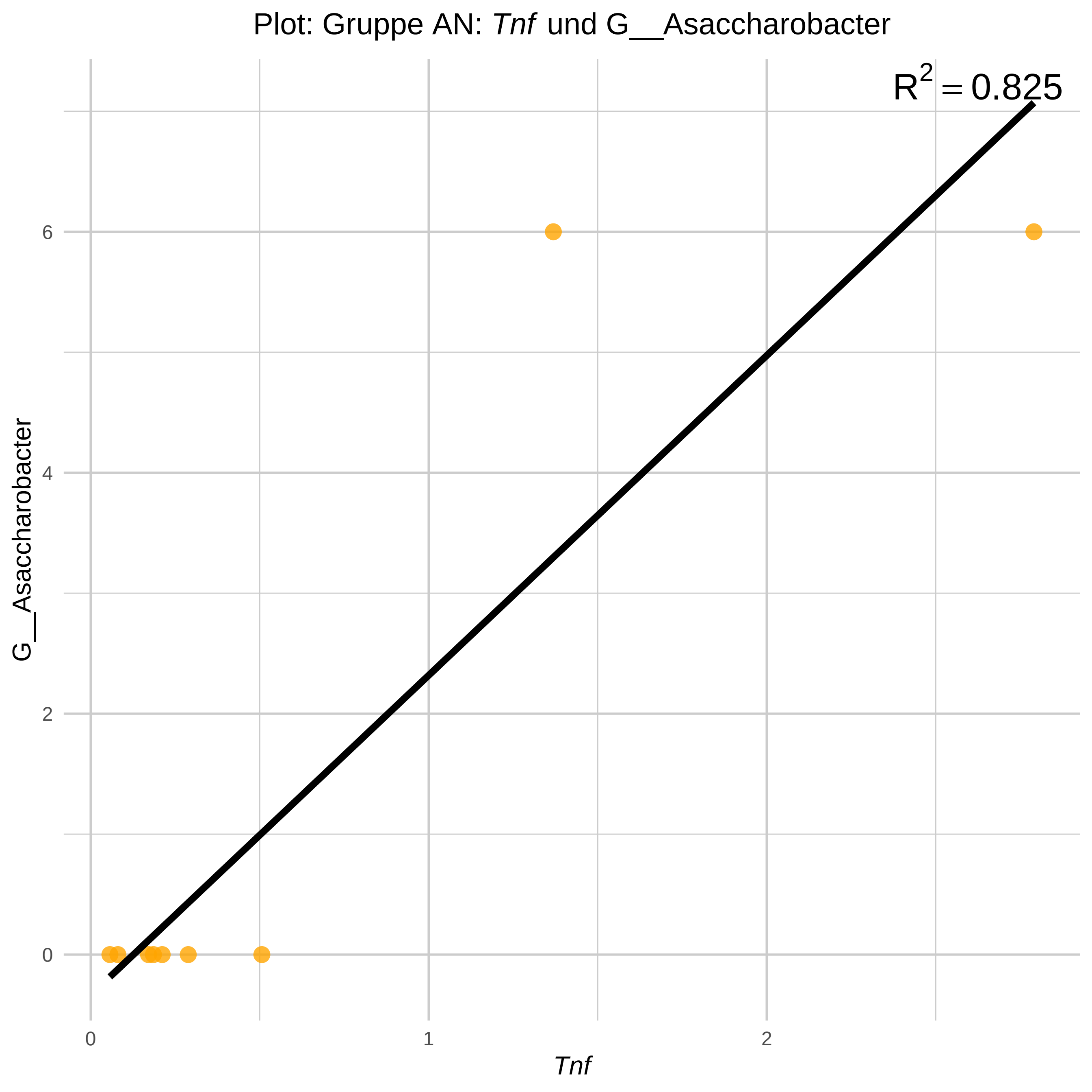

Plot: Gruppe AN: *Tnf* und G__Asaccharobacter
$R^2 = 0.825$
6
4
2
0
G__Asaccharobacter
0
1
2
Tnf

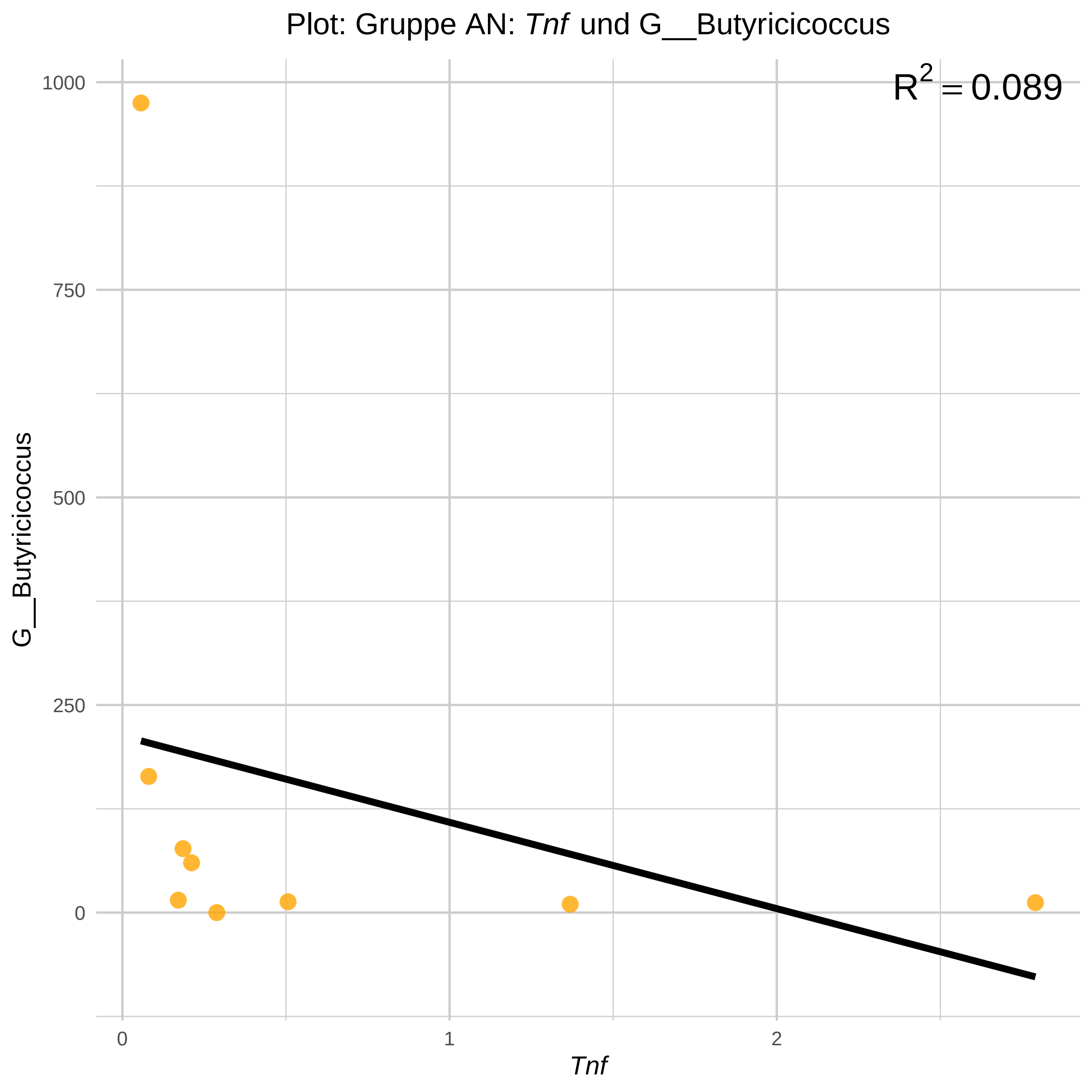

Plot: Gruppe AN: Tnf und G__Butyricicoccus
R² = 0.089
1000
750
500
250
0
G__Butyricicoccus
0
1
2
Tnf

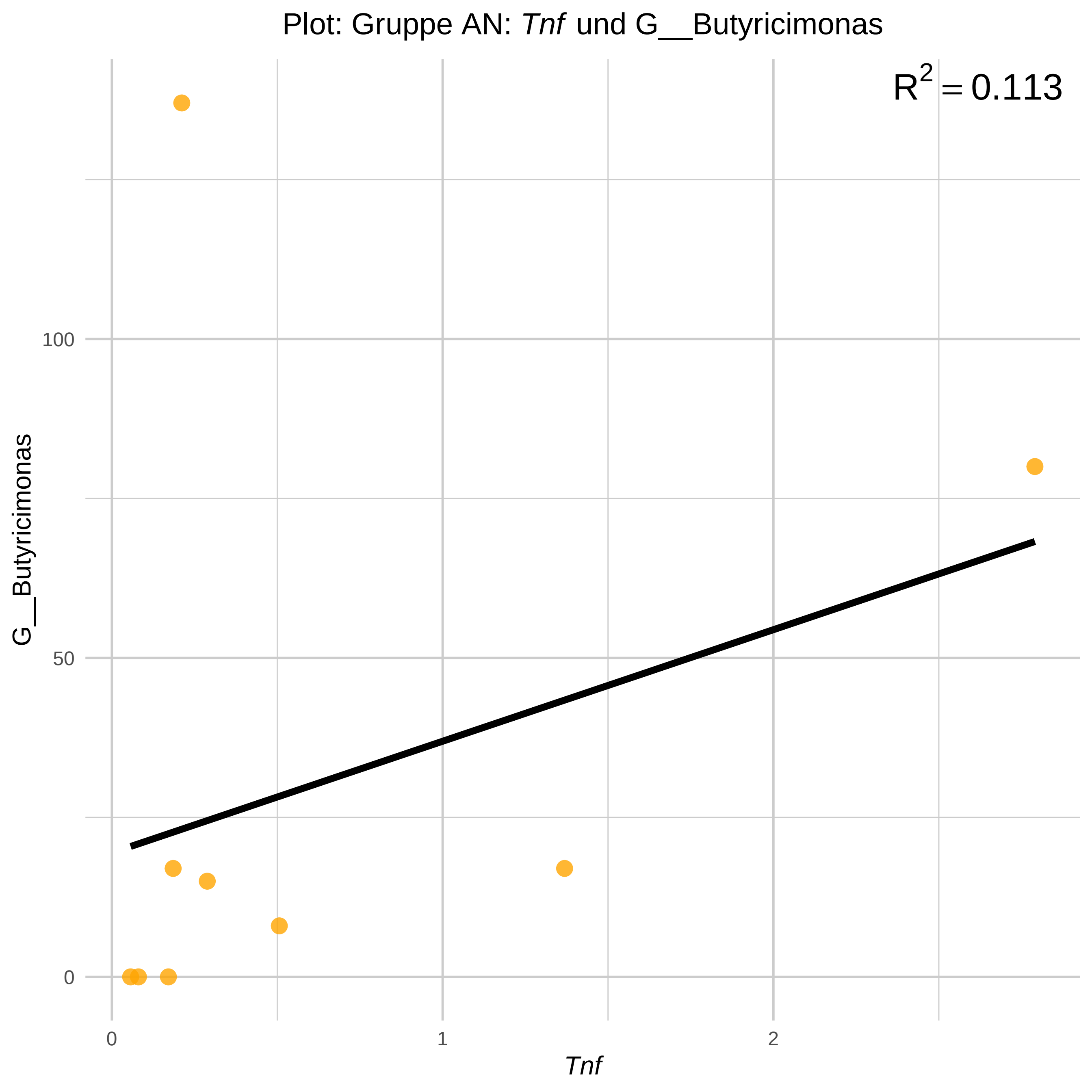

Plot: Gruppe AN: Tnf und G__Butyricimonas
$R^2 = 0.113$
G__Butyricimonas
100
50
0
0
1
2
Tnf

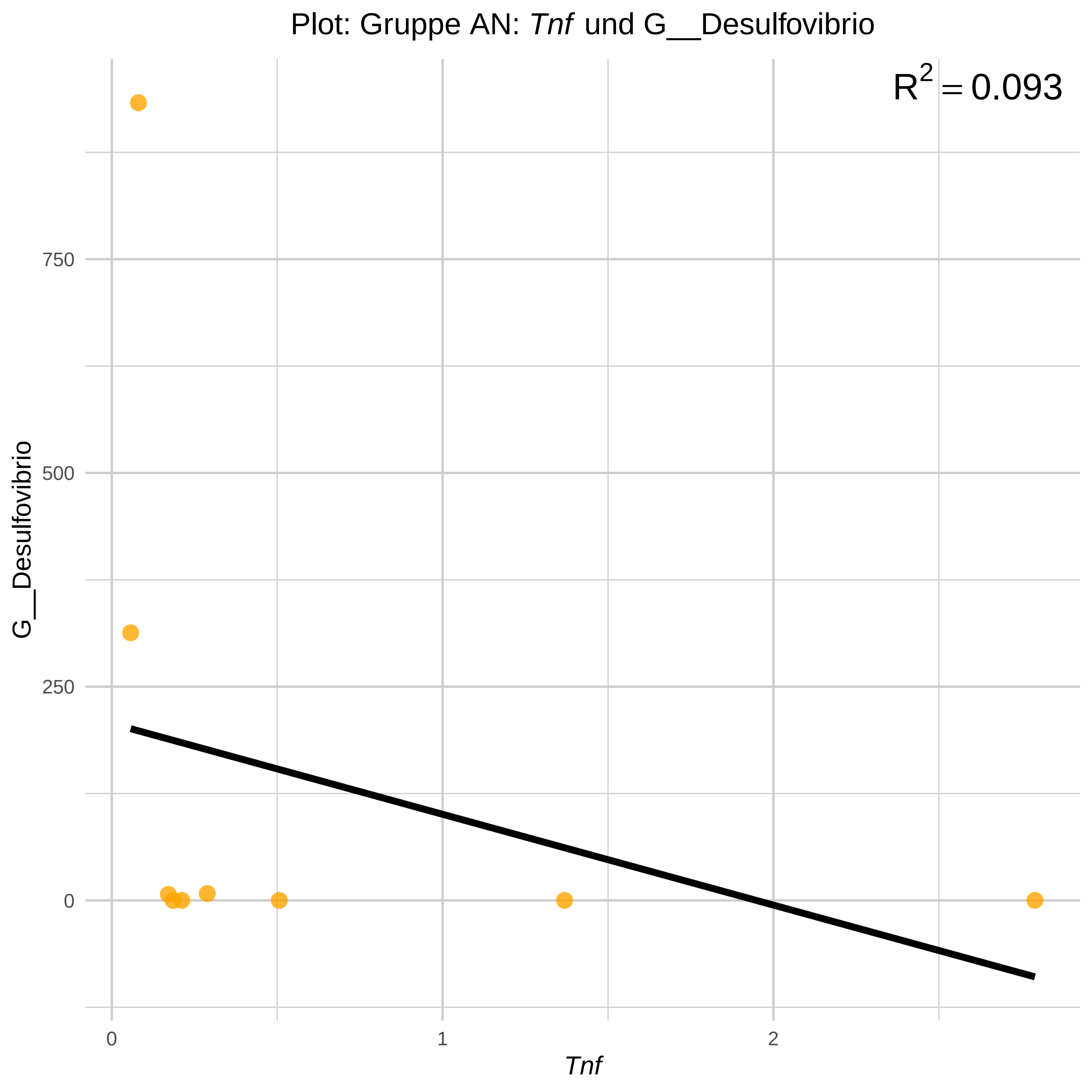

Plot: Gruppe AN: *Tnf* und G__Desulfovibrio
$R^2 = 0.093$
750
500
250
0
G__Desulfovibrio
0
1
2
Tnf

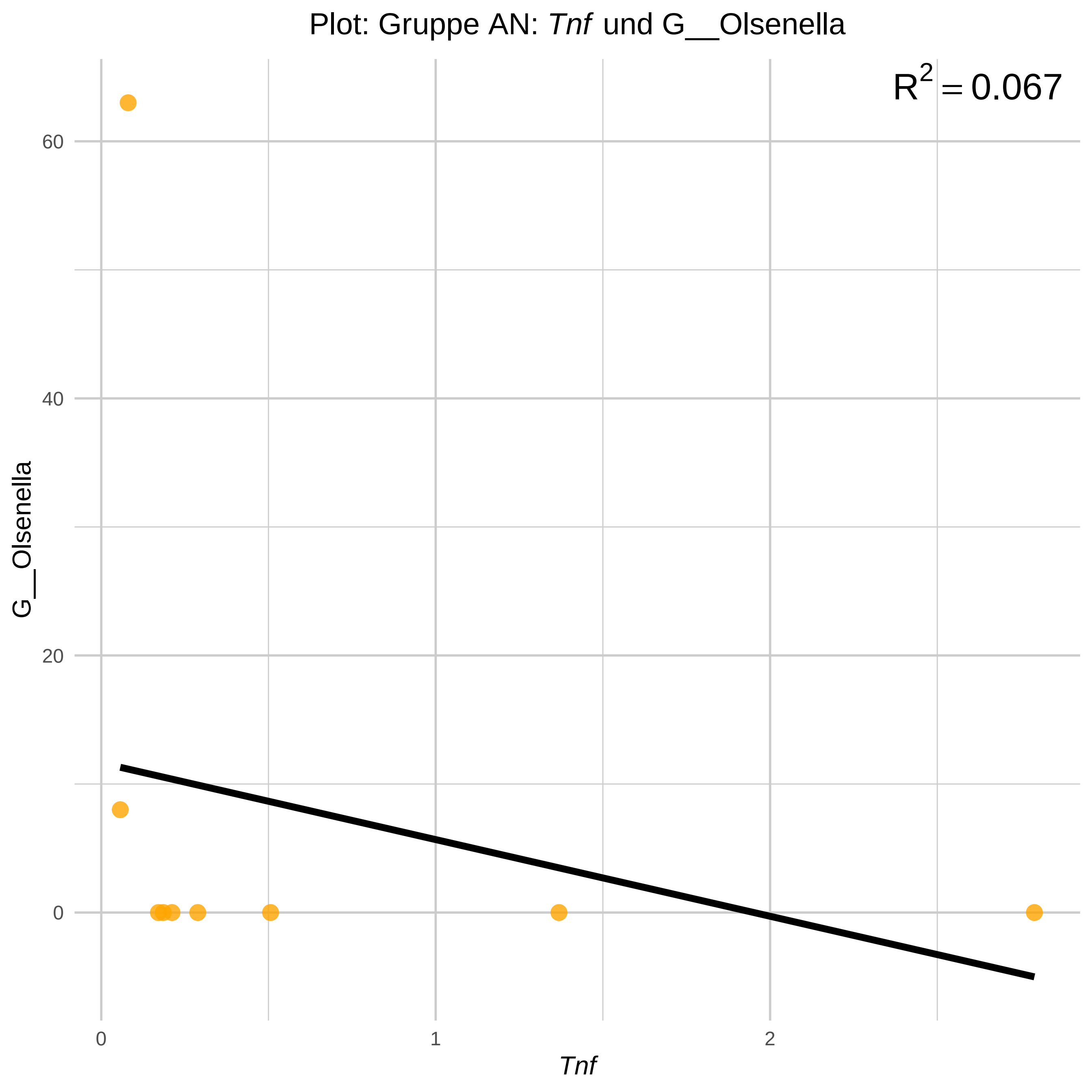

Plot: Gruppe AN: Tnf und G__Olsenella
R2 = 0.067
60
40
20
0
G__Olsenella
0
1
2
Tnf

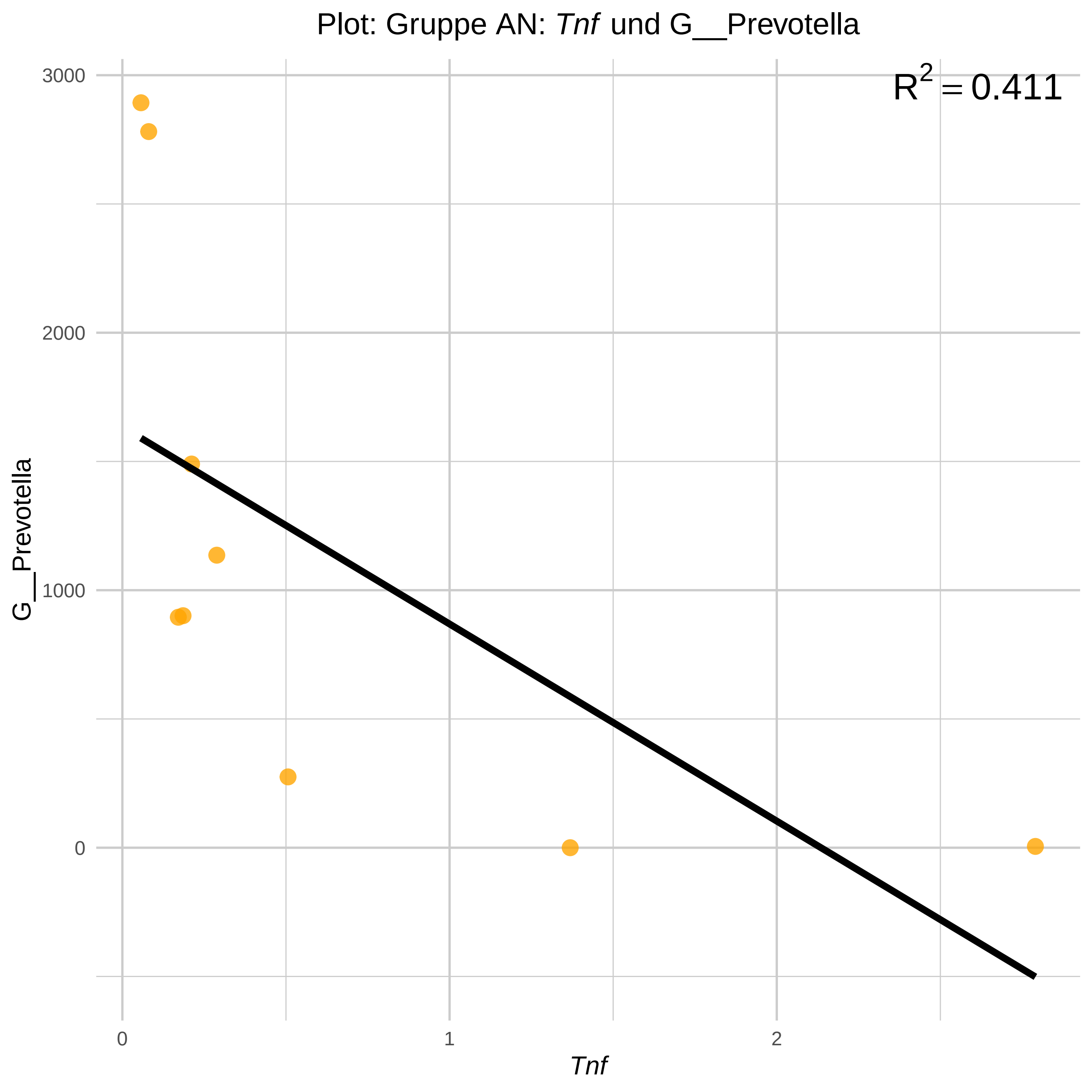

Plot: Gruppe AN: Tnf und G__Prevotella
R2 = 0.411
3000
2000
1000
0
G__Prevotella
0
1
2
Tnf

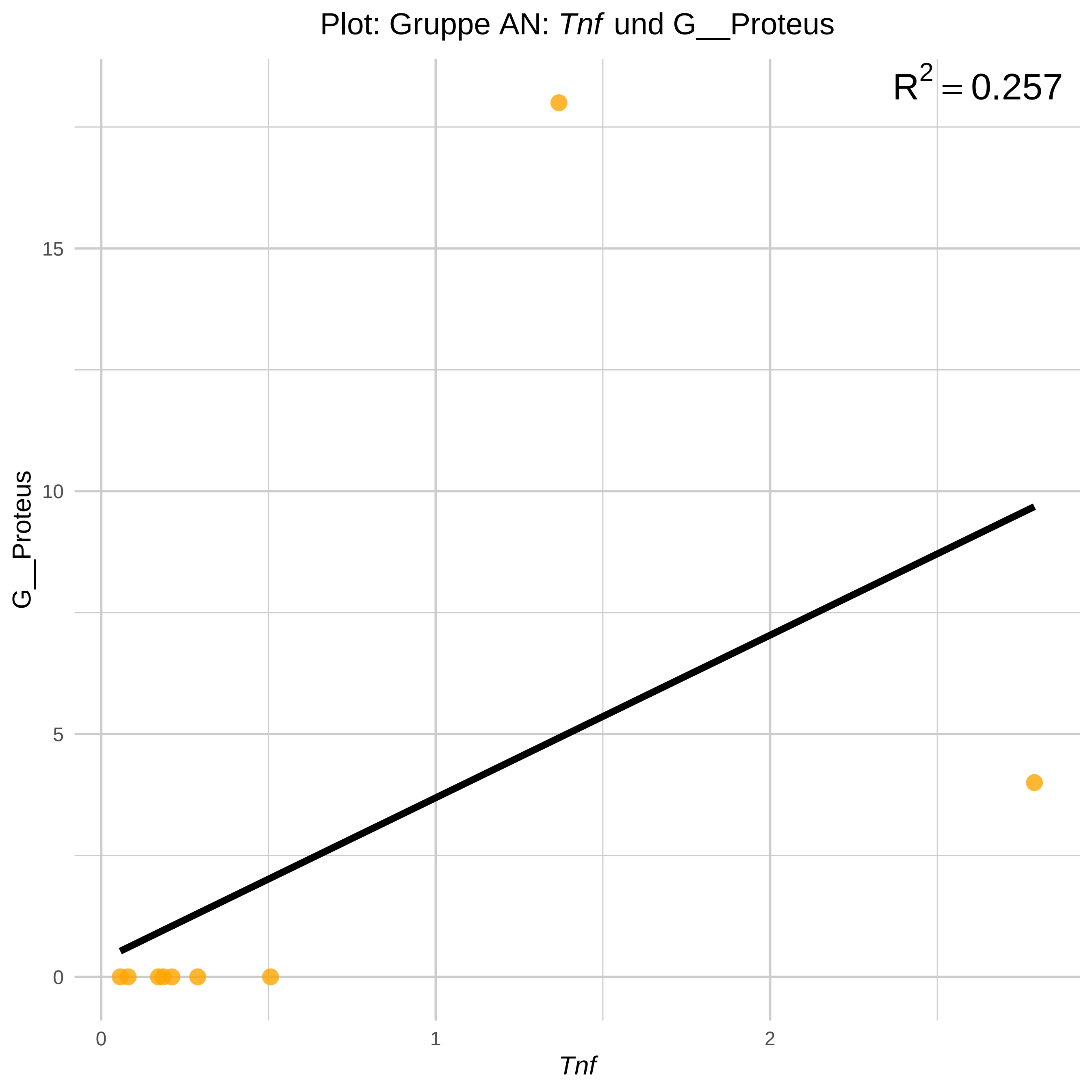

Plot: Gruppe AN: *Tnf* und G__Proteus
$R^2 = 0.257$
G__Proteus
0
5
10
15
0
1
2
Tnf

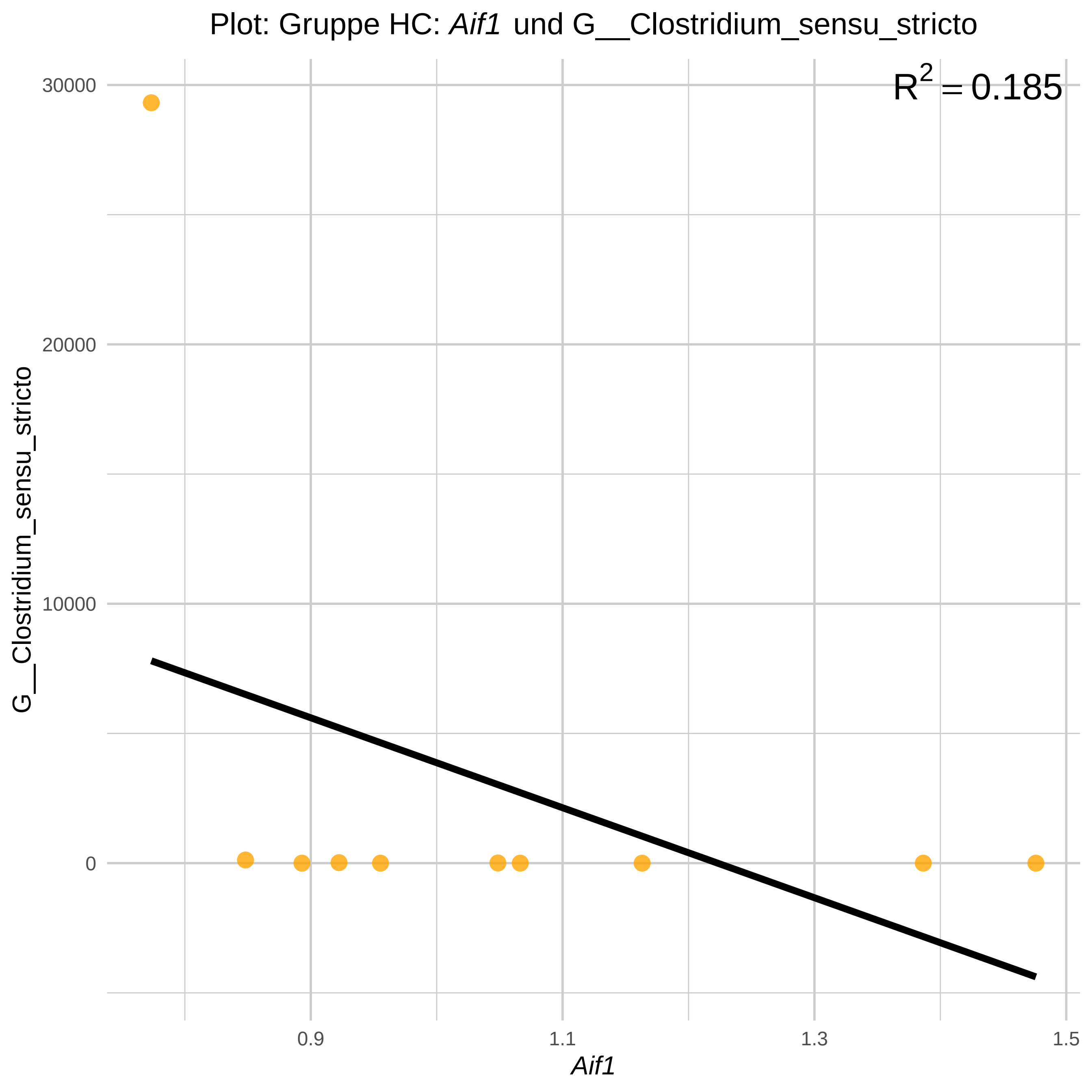
Plot: Gruppe HC: Aif1 und G__Clostridium_sensu_stricto
R2 = 0.185
30000
20000
10000
0
0.9
1.1
1.3
1.5
Aif1
G__Clostridium_sensu_stricto

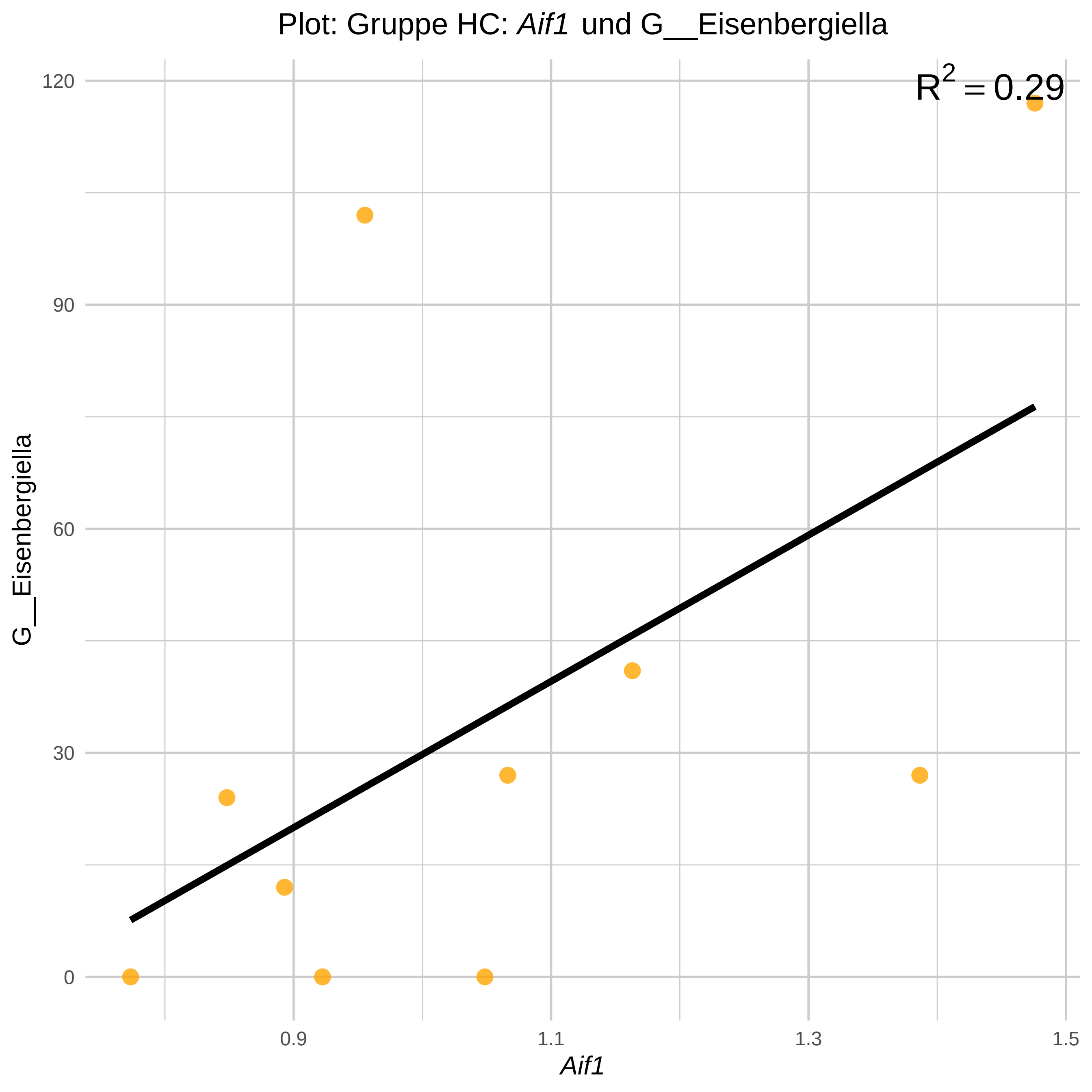

Plot: Gruppe HC: Aif1 und G__Eisenbergiella
$R^2 = 0.29$
120
90
60
30
0
G__Eisenbergiella
0.9
1.1
1.3
1.5
Aif1

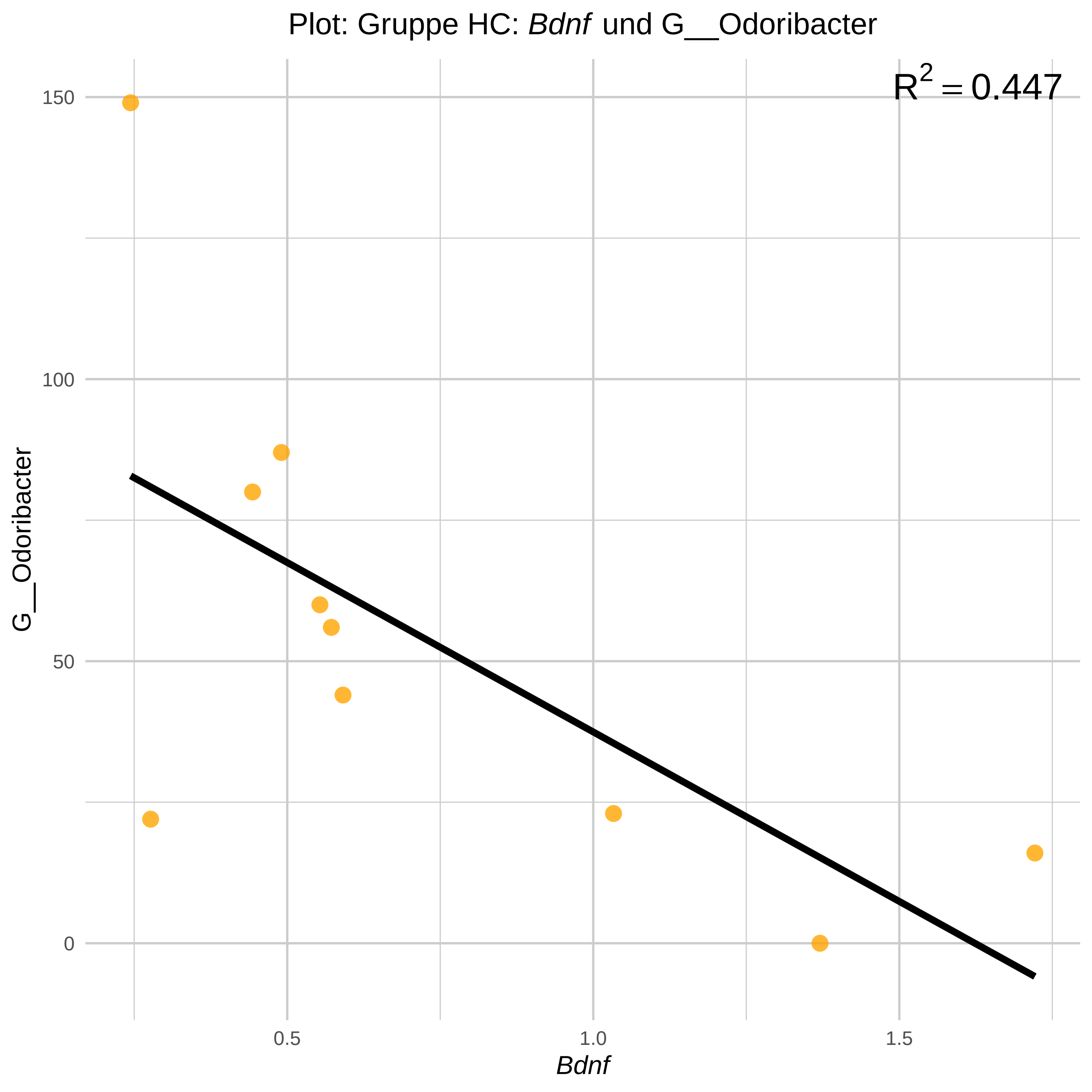

Plot: Gruppe HC: *Bdnf* und G__Odoribacter
$R^2 = 0.447$
150
100
50
0
G__Odoribacter
0.5
1.0
1.5
Bdnf

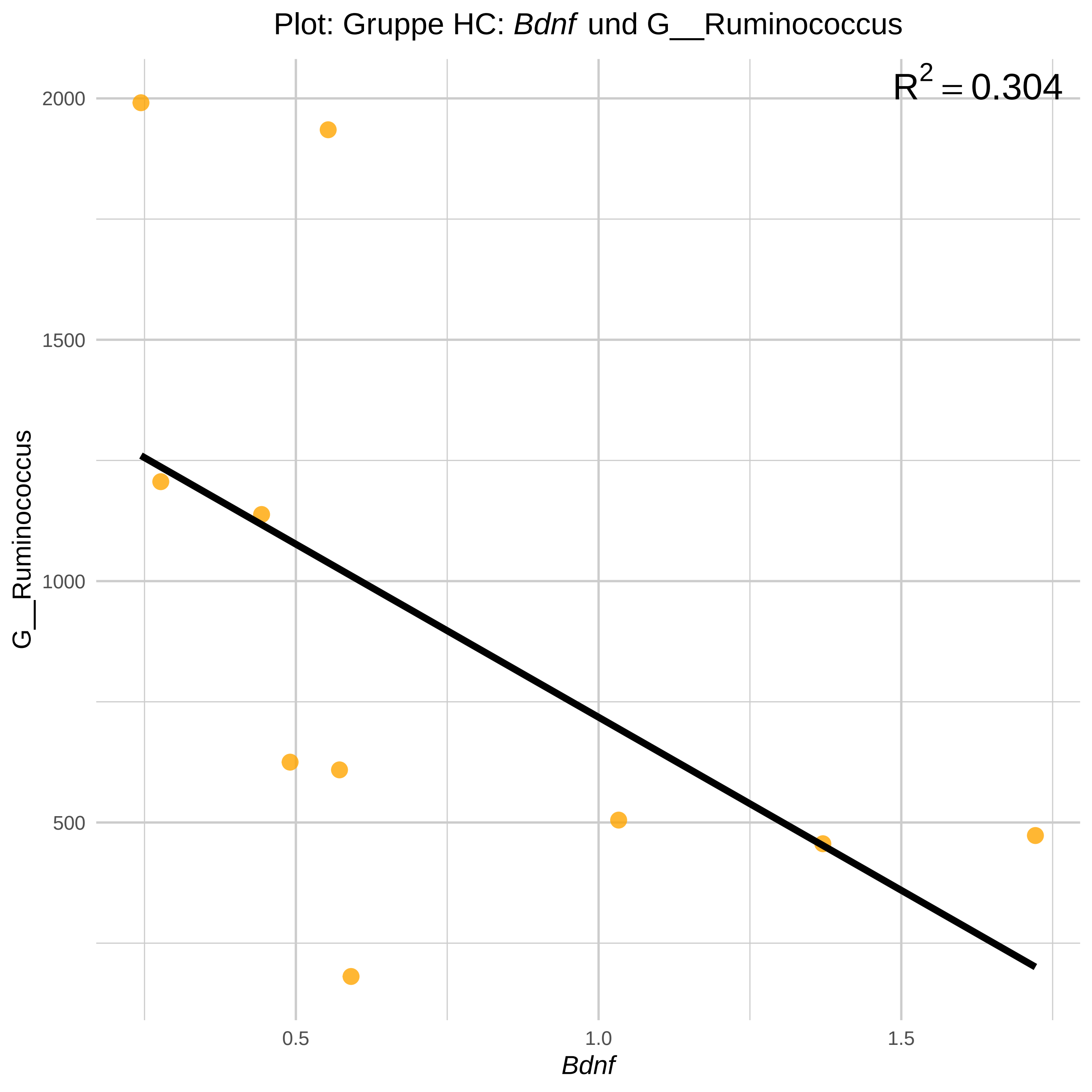

Plot: Gruppe HC: *Bdnf* und G__Ruminococcus
$R^2 = 0.304$
2000
1500
1000
500
G__Ruminococcus
0.5
1.0
1.5
Bdnf

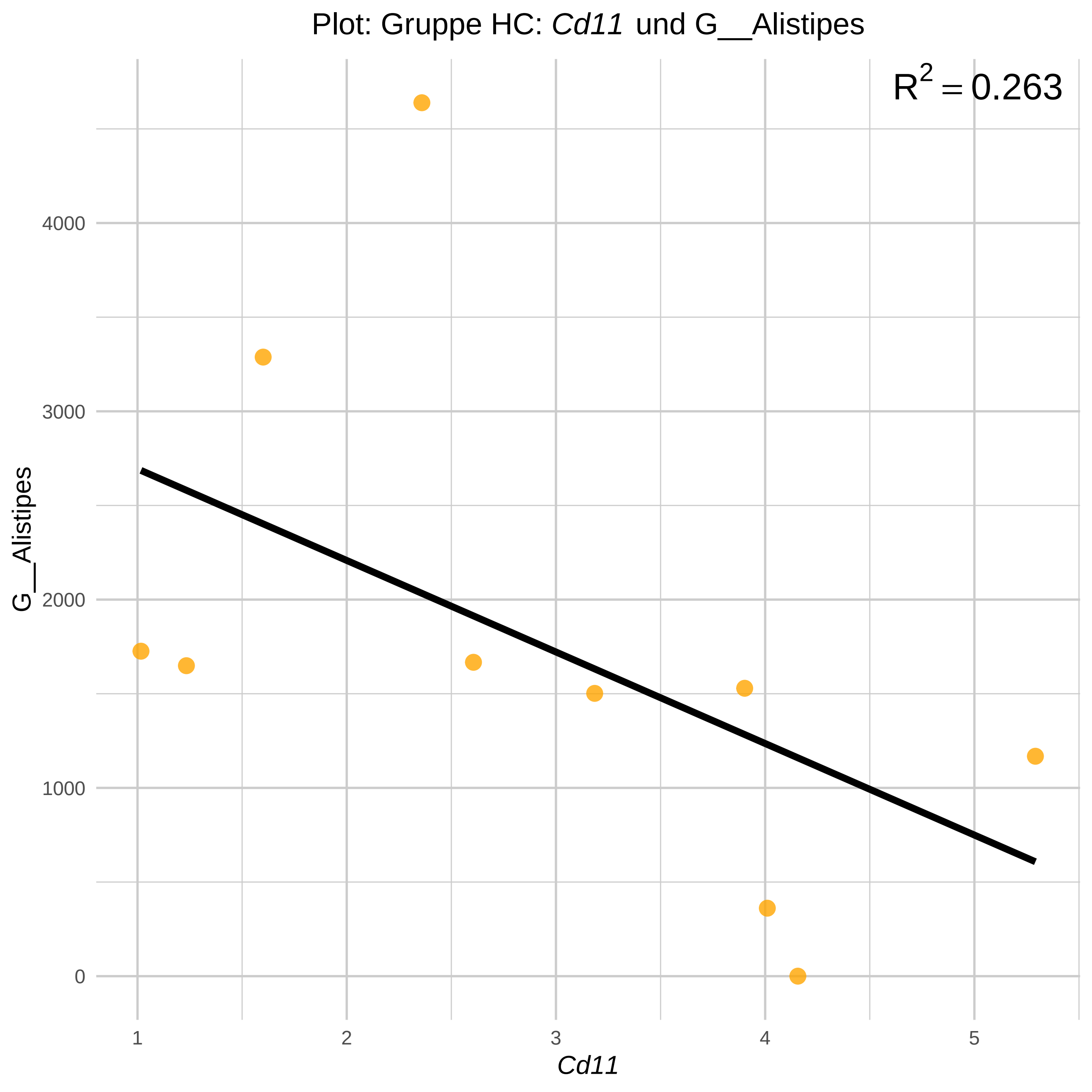

Plot: Gruppe HC: Cd11 und G__Alistipes
$R^2 = 0.263$
4000
3000
2000
1000
0
G__Alistipes
1
2
3
4
5
Cd11

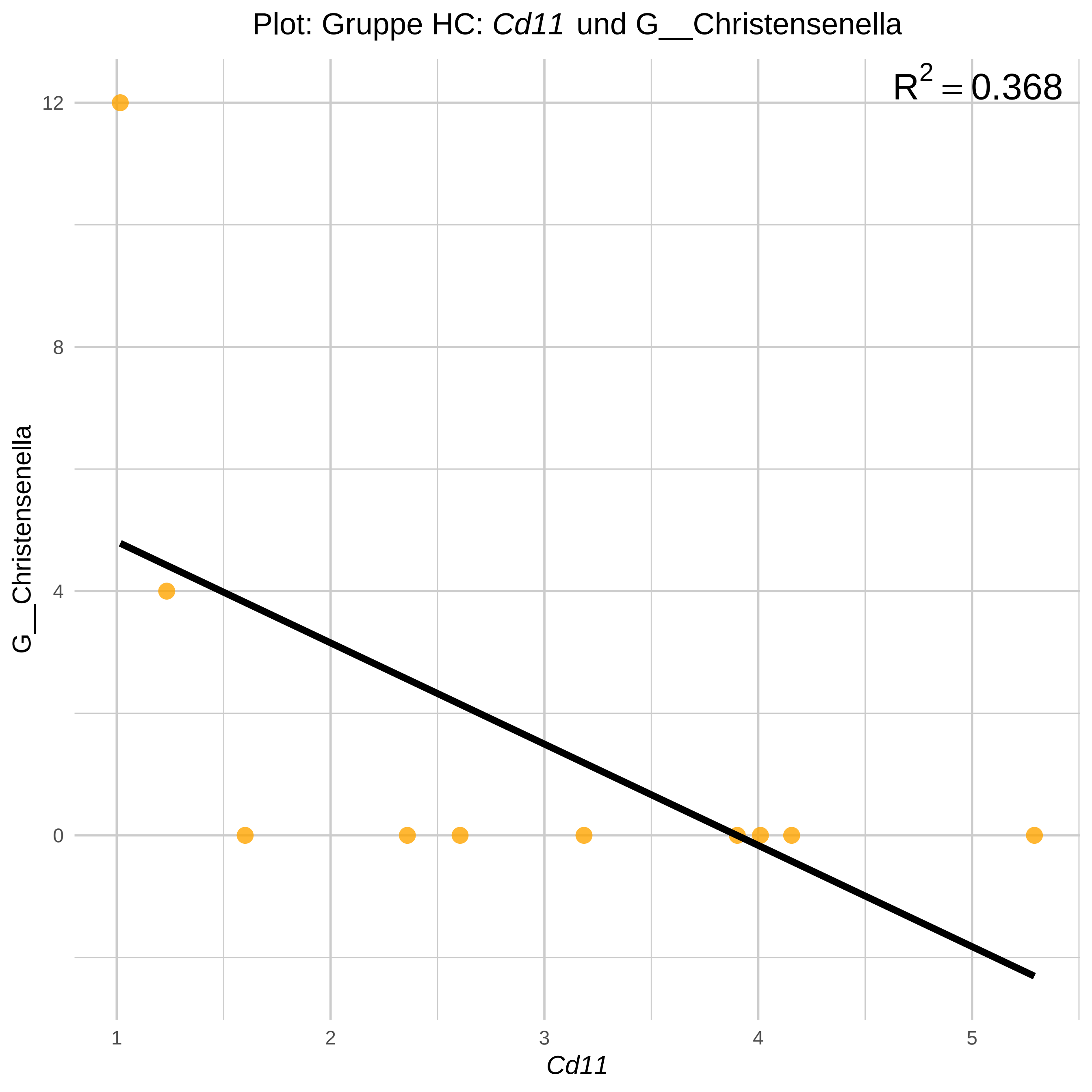

Plot: Gruppe HC: *Cd11* und G__Christensenella
$R^2 = 0.368$
12
8
4
0
G__Christensenella
1
2
3
4
5
Cd11

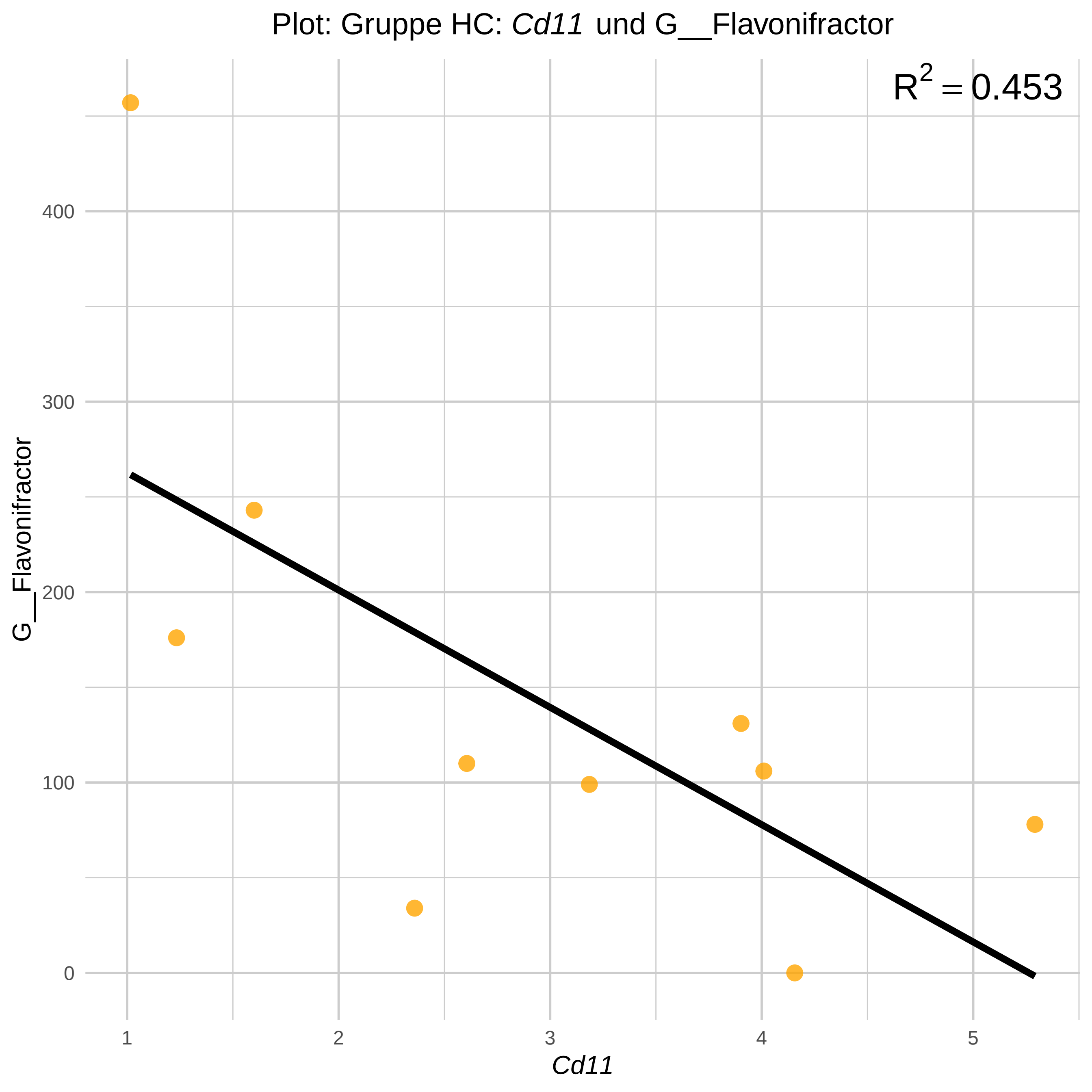

Plot: Gruppe HC: *Cd11* und G__Flavonifractor
$R^2 = 0.453$
400
300
200
100
0
G__Flavonifractor
1
2
3
4
5
Cd11

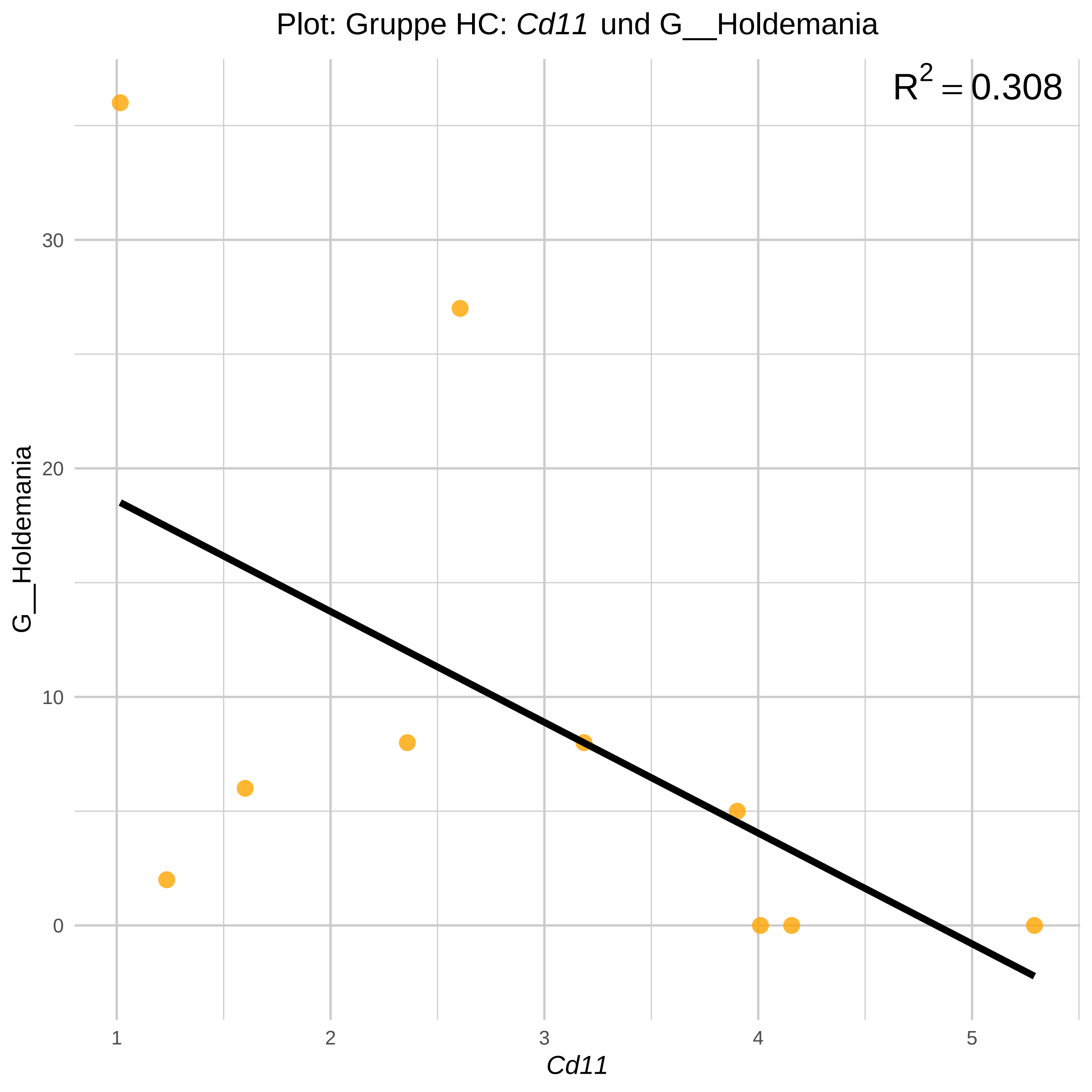

Plot: Gruppe HC: Cd11 und G__Holdemania
$R^2 = 0.308$
G__Holdemania
Cd11
0
10
20
30
1
2
3
4
5

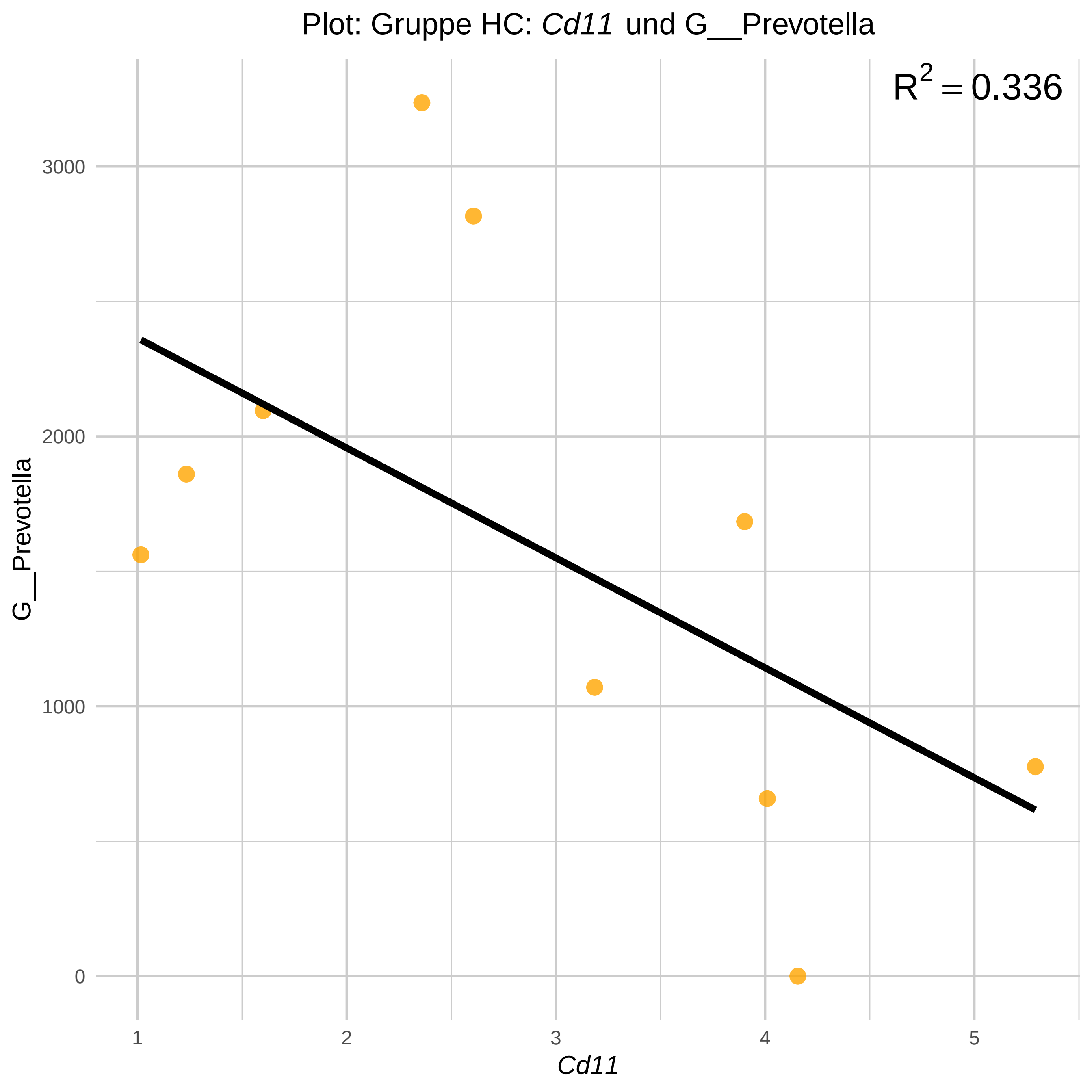

Plot: Gruppe HC: *Cd11* und G__Prevotella
$R^2 = 0.336$
G__Prevotella
3000
2000
1000
0
1
2
3
4
5
Cd11

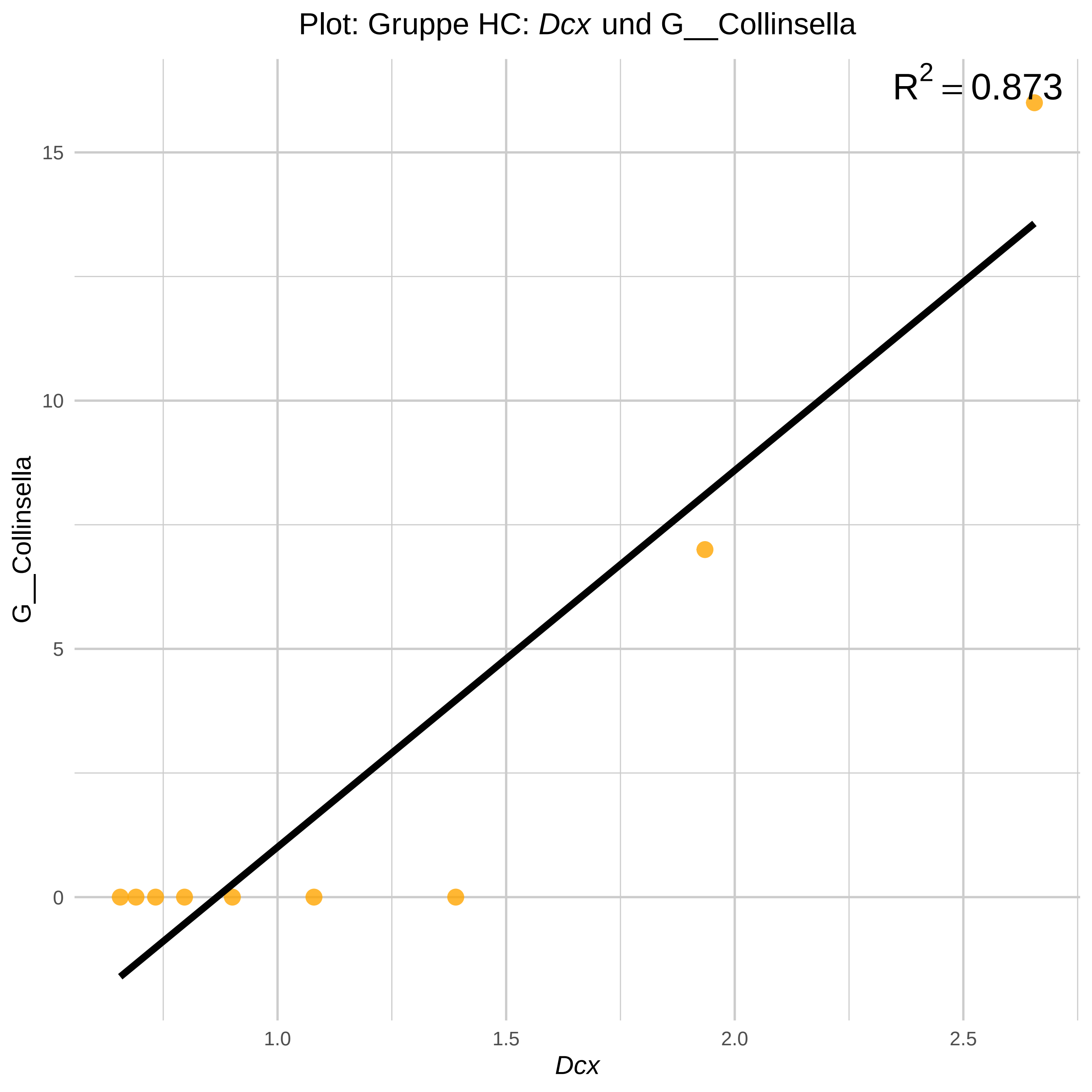

Plot: Gruppe HC: *Dcx* und G__Collinsella
$R^2 = 0.873$
15
10
5
0
G__Collinsella
1.0
1.5
2.0
2.5
Dcx

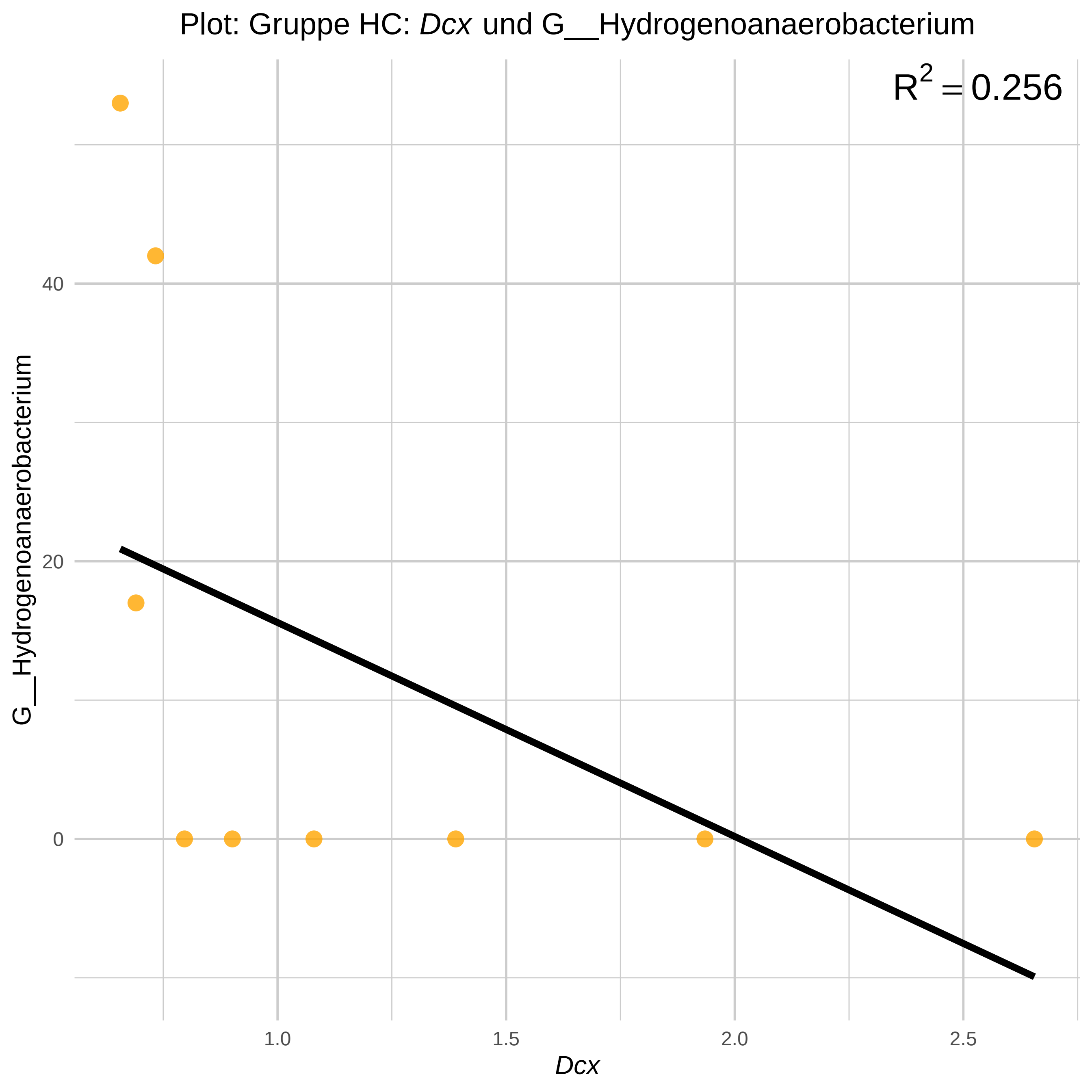

Plot: Gruppe HC: *Dcx* und G__Hydrogenoanaerobacterium
$R^2 = 0.256$
40
20
0
G__Hydrogenoanaerobacterium
1.0
1.5
2.0
2.5
Dcx

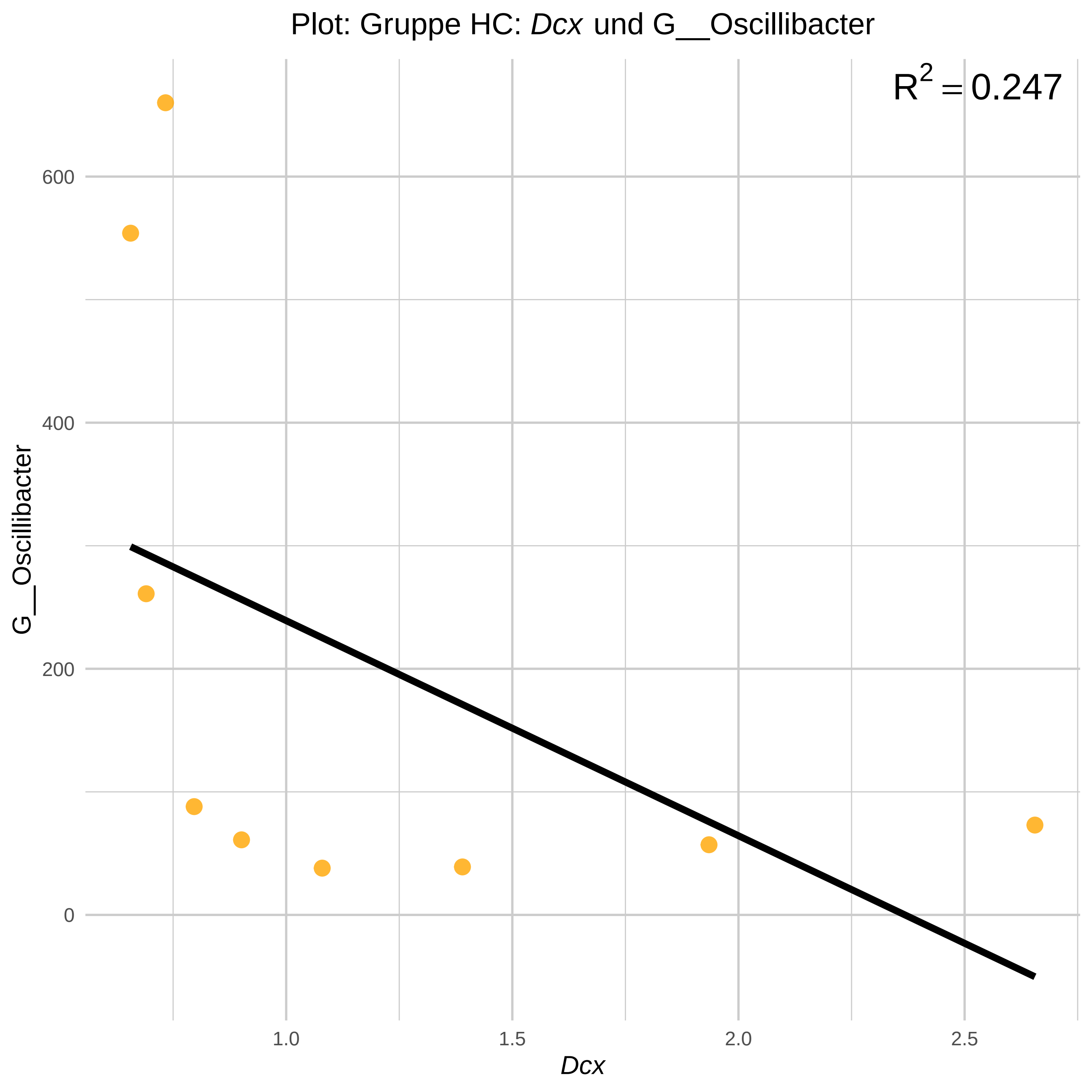

Plot: Gruppe HC: *Dcx* und G__Oscillibacter
$R^2 = 0.247$
600
400
200
0
G__Oscillibacter
1.0
1.5
2.0
2.5
Dcx

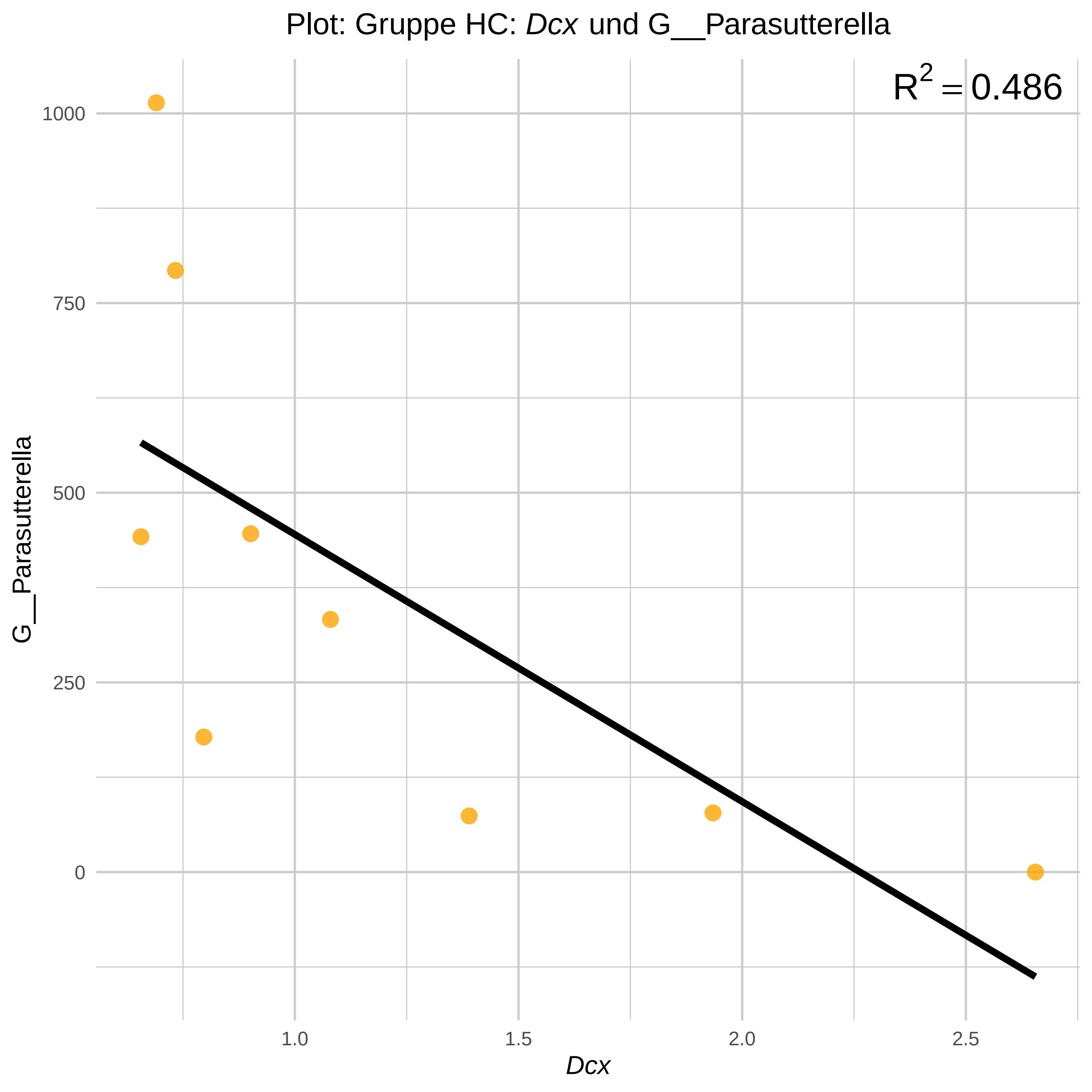

Plot: Gruppe HC: *Dcx* und G__Parasutterella
$R^2 = 0.486$
1000
750
500
250
0
G__Parasutterella
1.0
1.5
2.0
2.5
Dcx

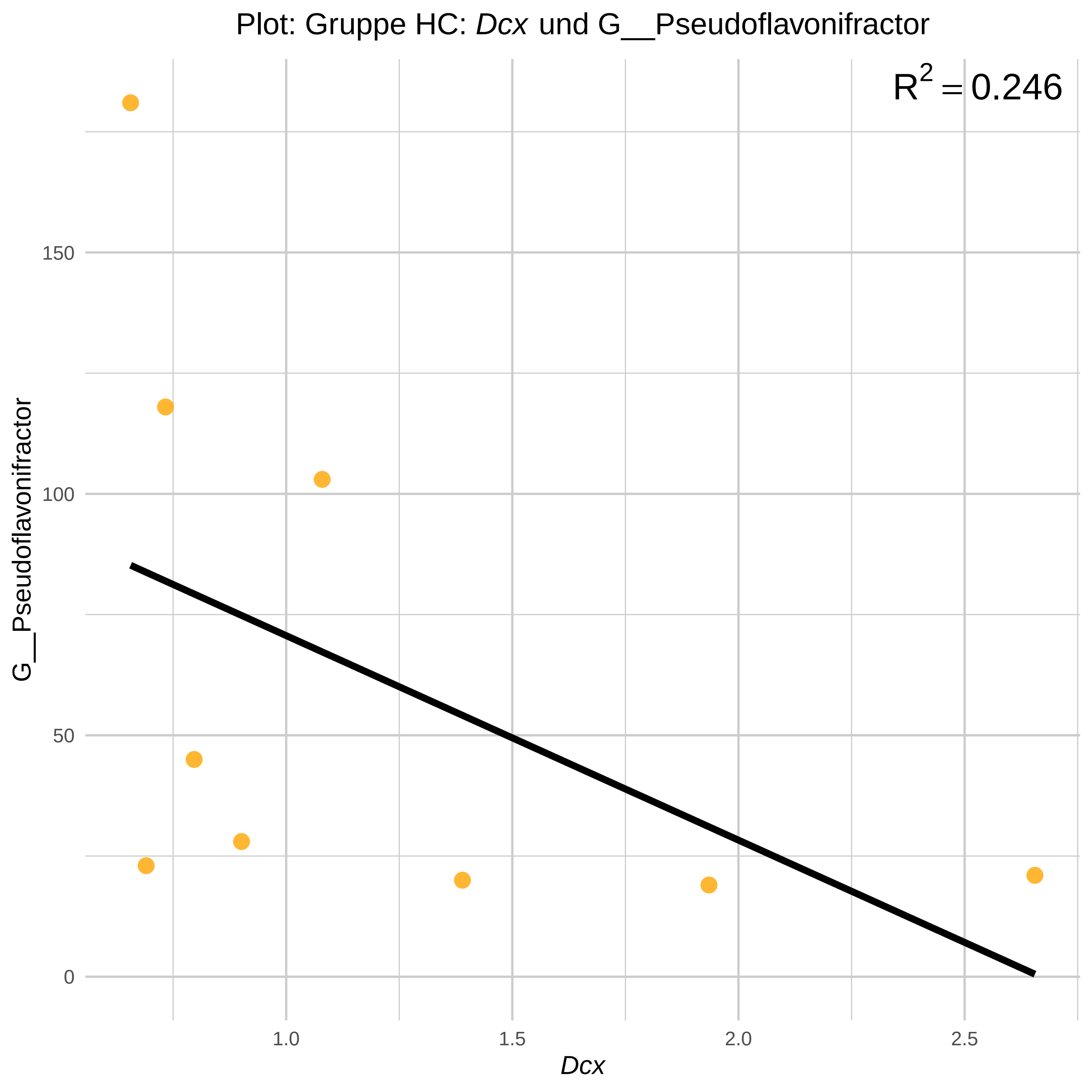

Plot: Gruppe HC: Dcx und G__Pseudoflavonifractor
R2 = 0.246
G__Pseudoflavonifractor
150
100
50
0
1.0
1.5
2.0
2.5
Dcx

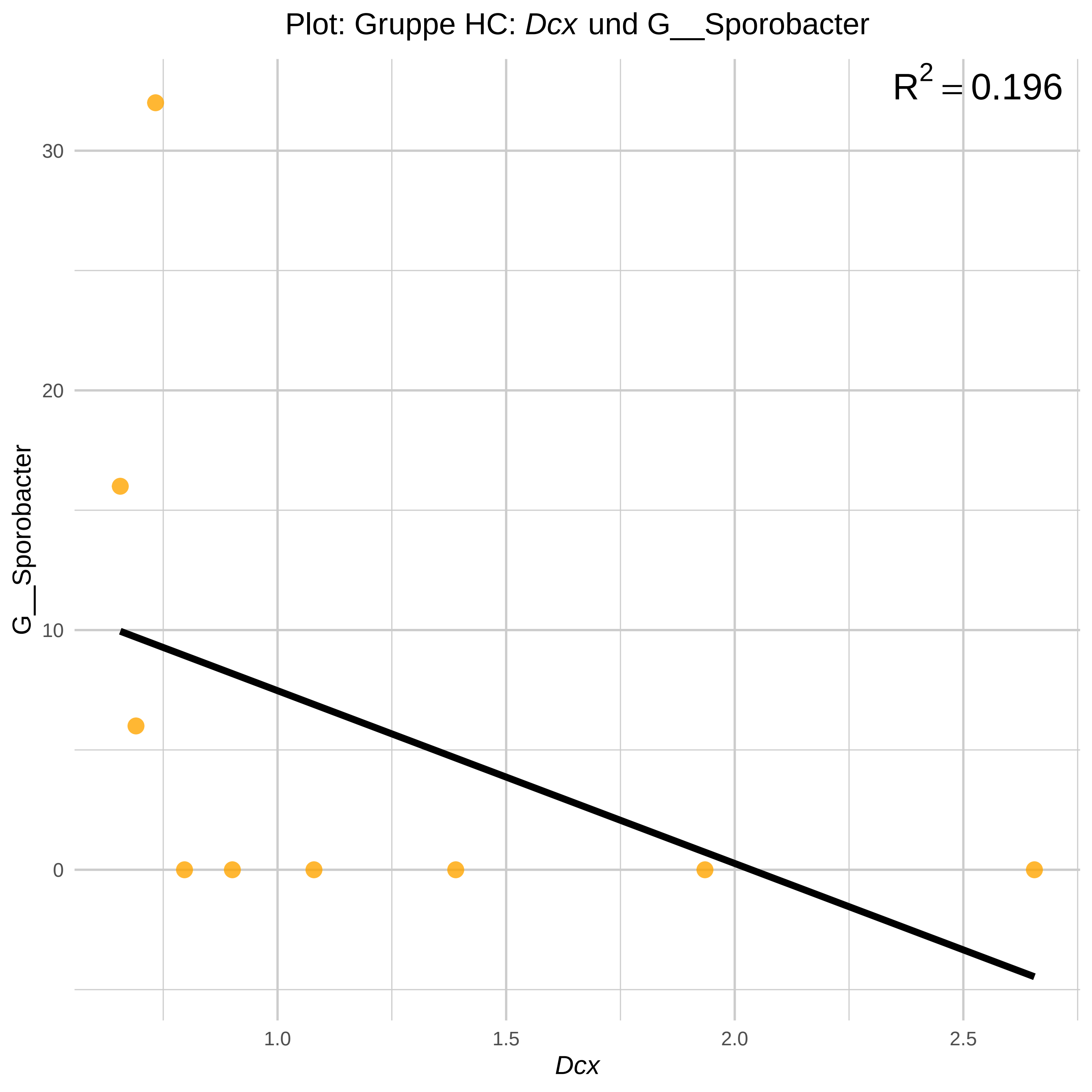

Plot: Gruppe HC: Dcx und G__Sporobacter
R2 = 0.196
30
20
10
0
G__Sporobacter
1.0
1.5
2.0
2.5
Dcx

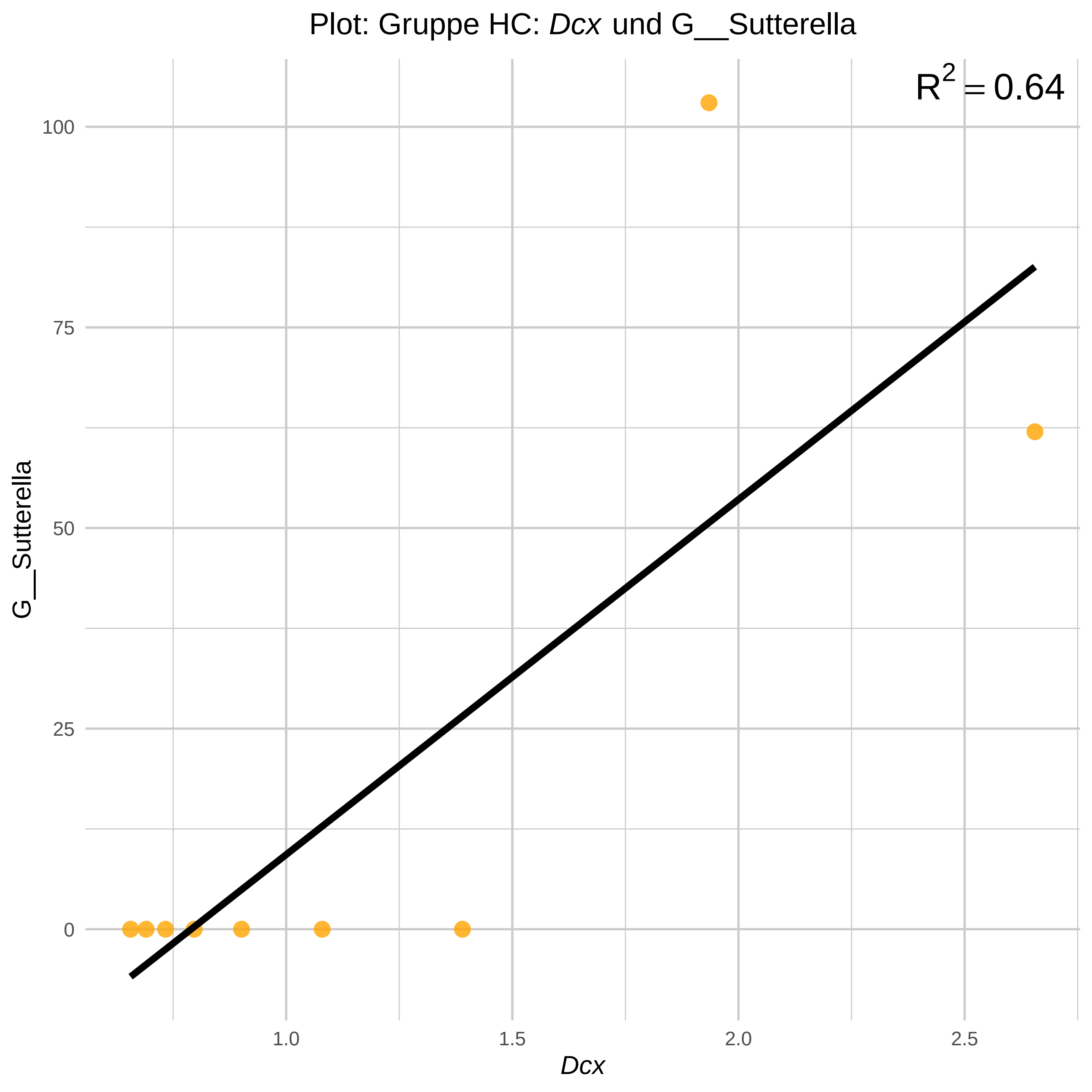
Plot: Gruppe HC: Dcx und G__Sutterella
R² = 0.64
100
75
50
25
0
1.0
1.5
2.0
2.5
Dcx
G__Sutterella

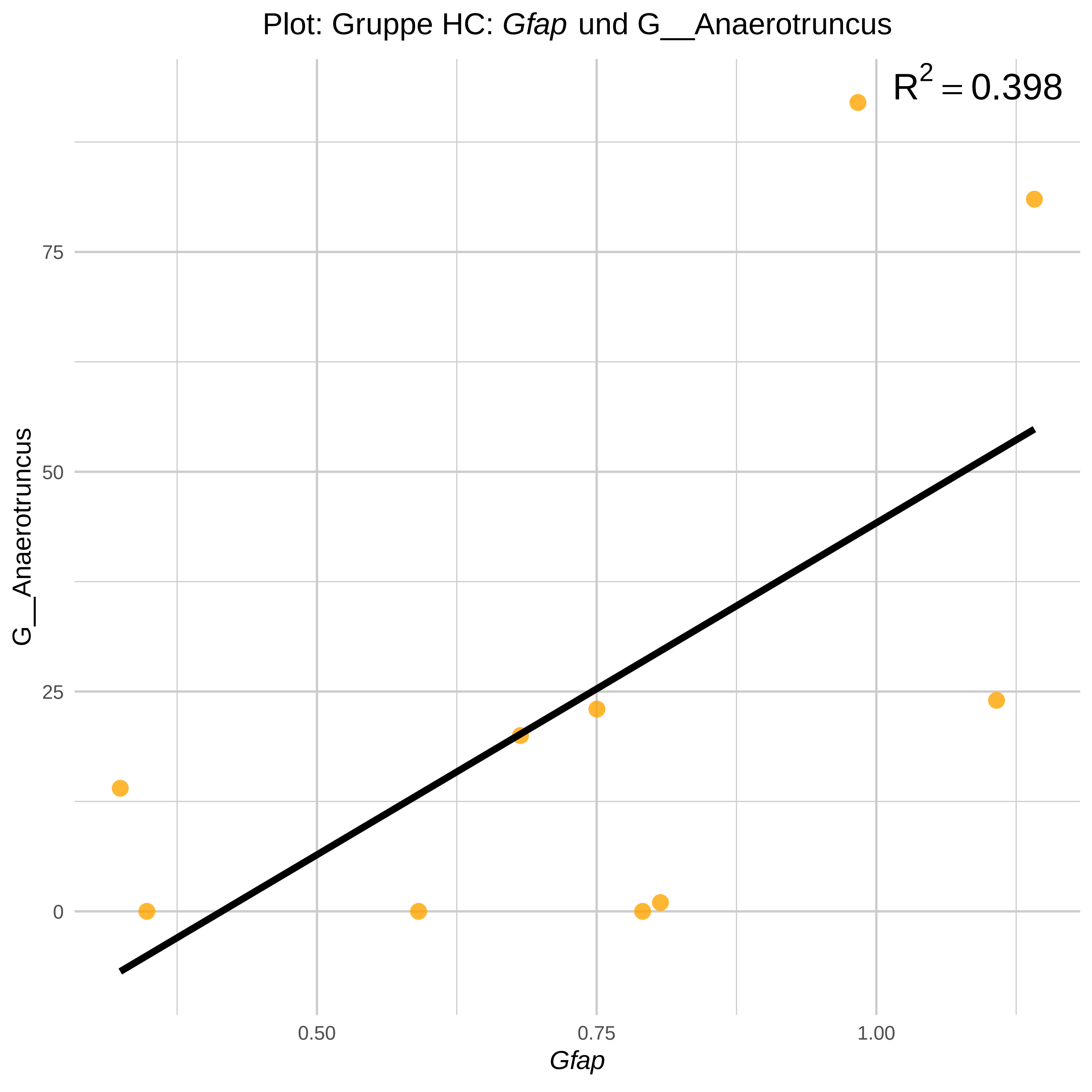
Plot: Gruppe HC: *Gfap* und G__Anaerotruncus
$R^2 = 0.398$
G__Anaerotruncus
75
50
25
0
0.50
0.75
1.00
Gfap

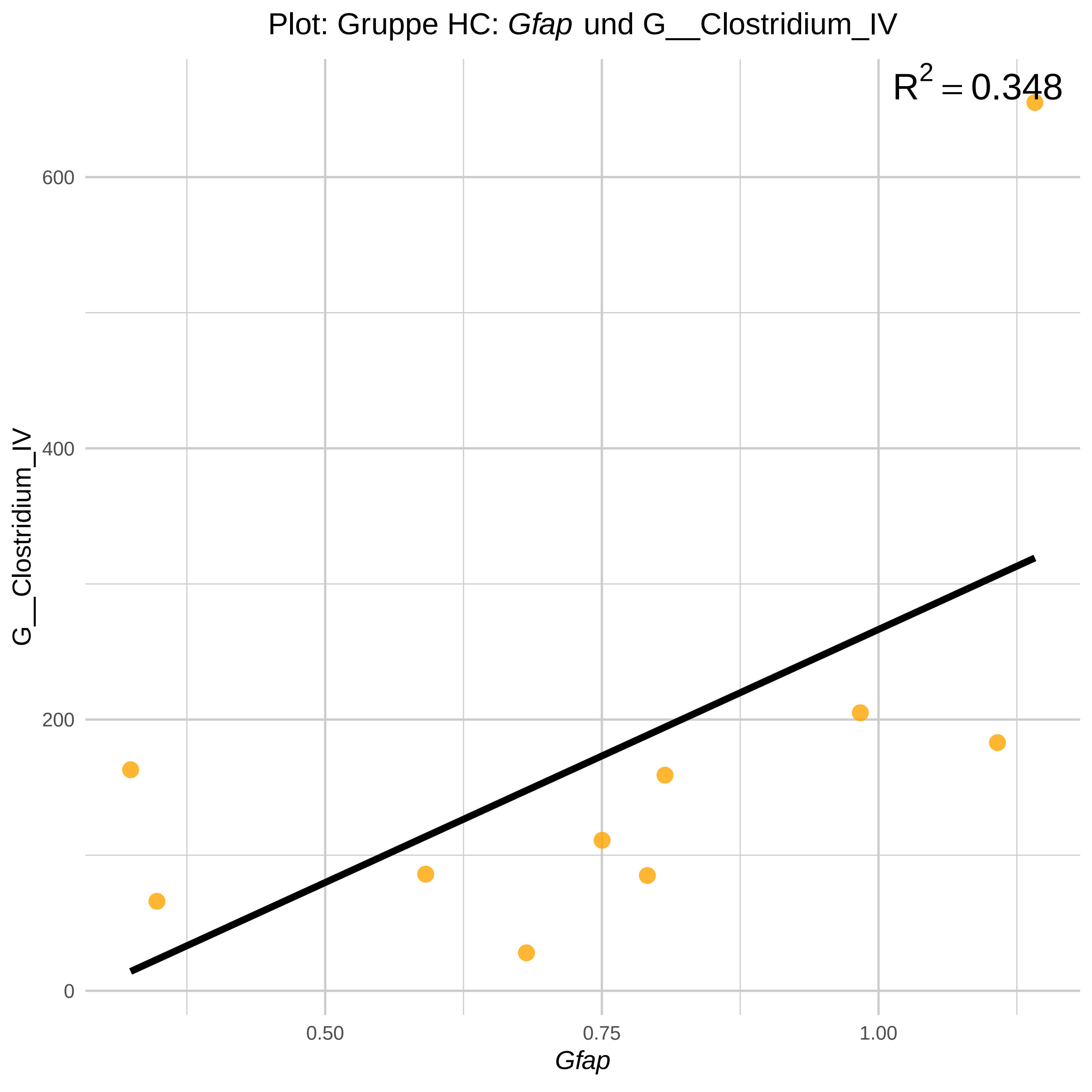

Plot: Gruppe HC: Gfap und G__Clostridium_IV
R² = 0.348
600
400
200
0
0.50
0.75
1.00
Gfap
G__Clostridium_IV

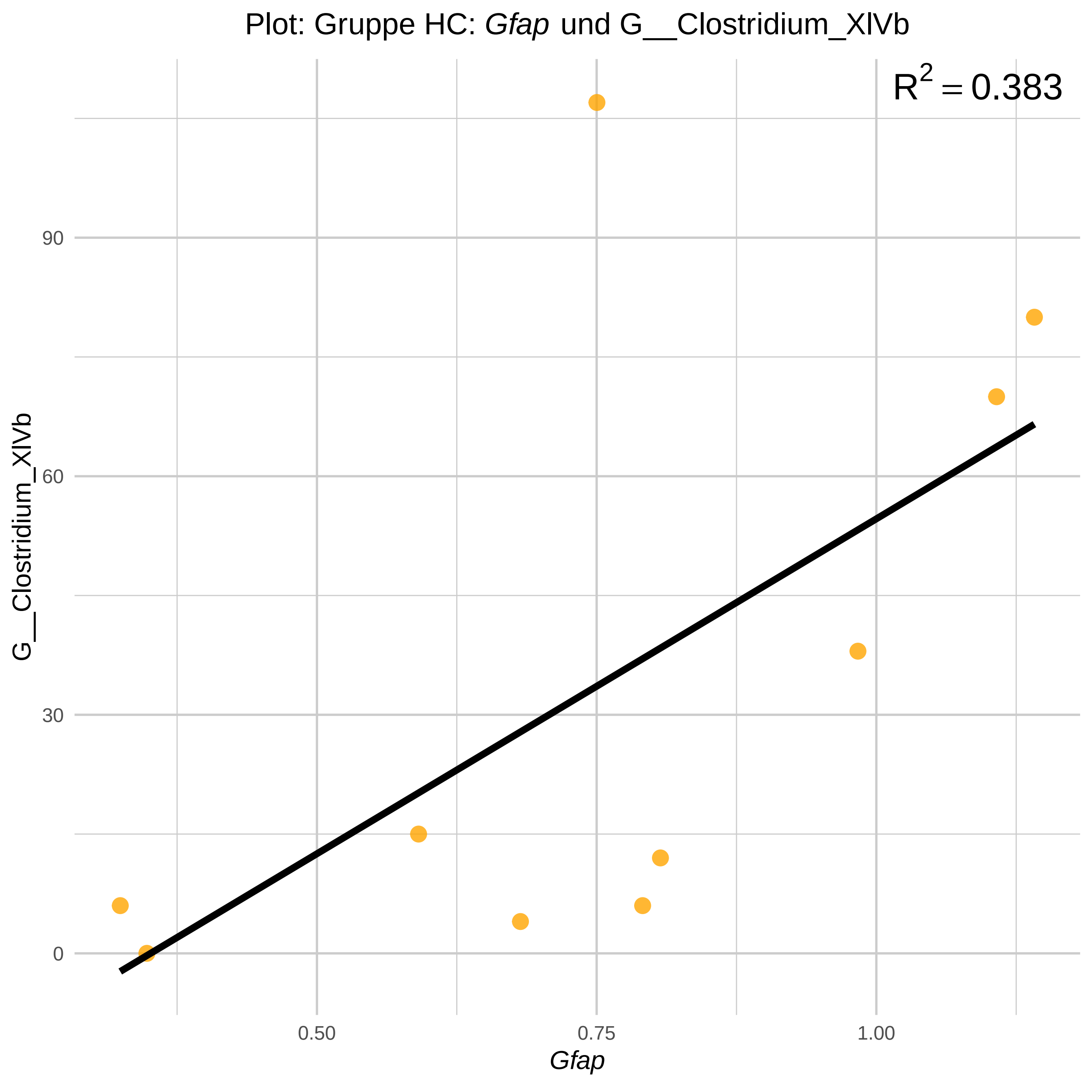
Plot: Gruppe HC: *Gfap* und G__Clostridium_XlVb
$R^2 = 0.383$
90
60
30
0
0.50
0.75
1.00
Gfap
G__Clostridium_XlVb

Plot: Gruppe HC: *Gfap* und G__Enterococcus

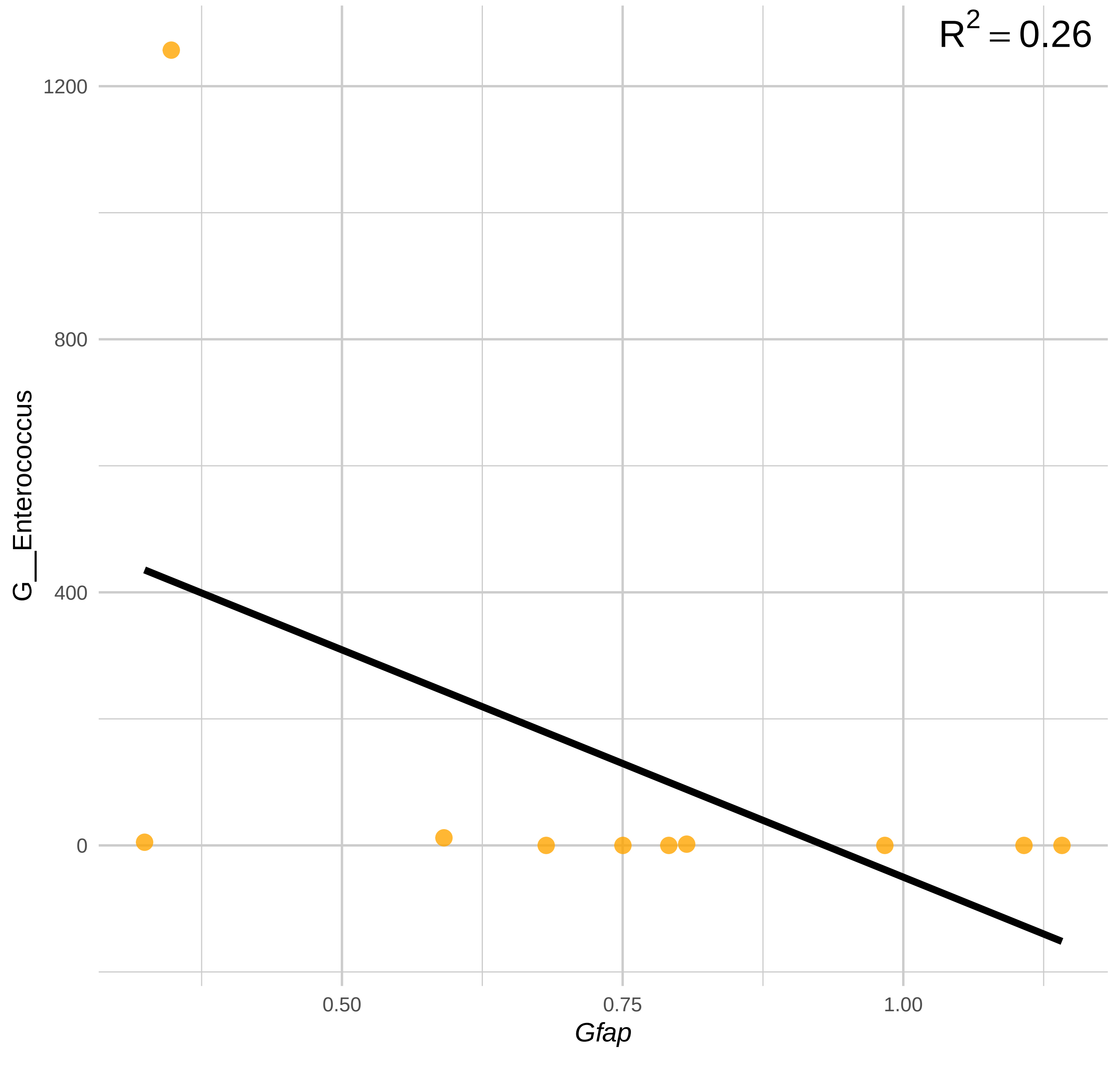

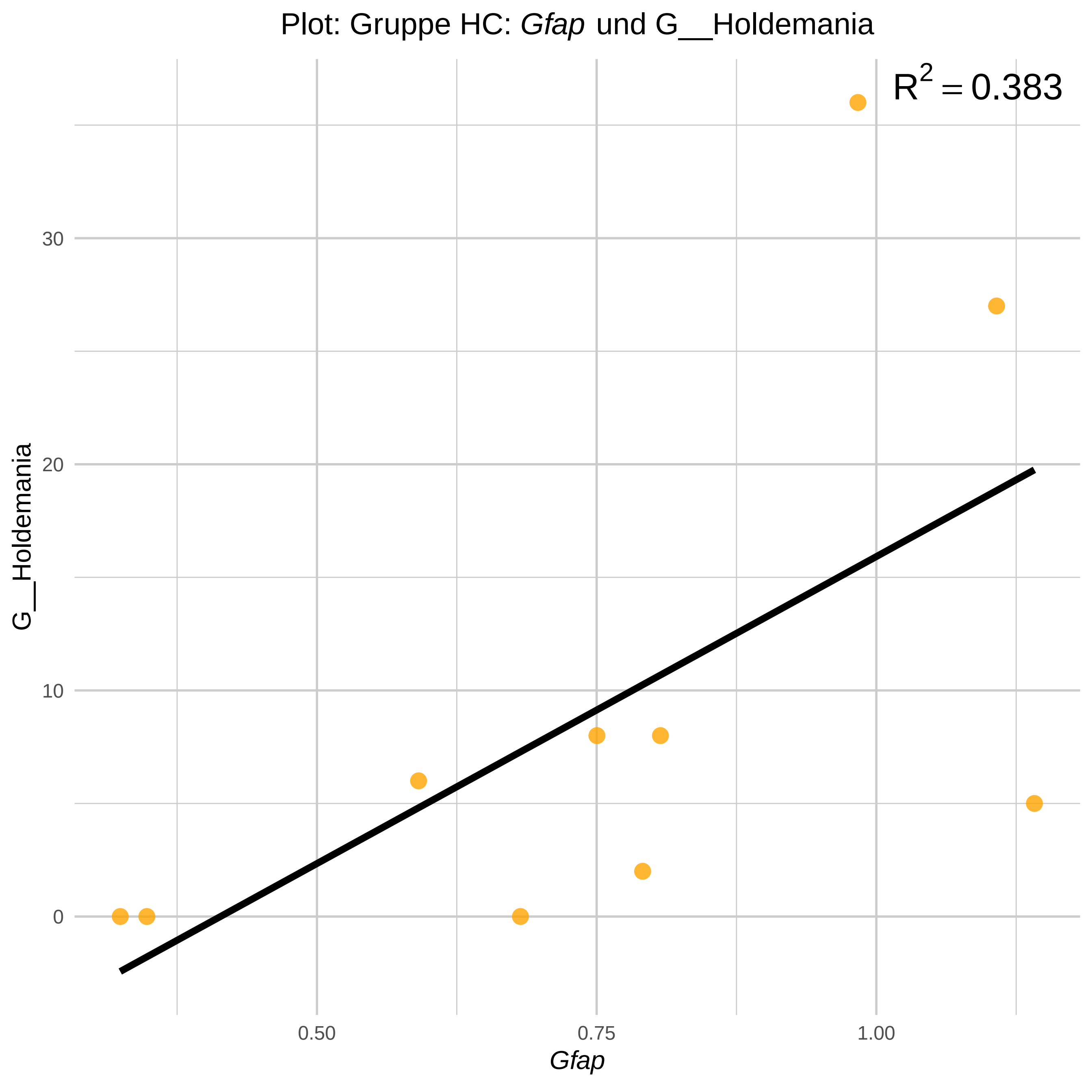

Plot: Gruppe HC: *Gfap* und G__Holdemania
$R^2 = 0.383$
30
20
10
0
G__Holdemania
0.50
0.75
1.00
Gfap

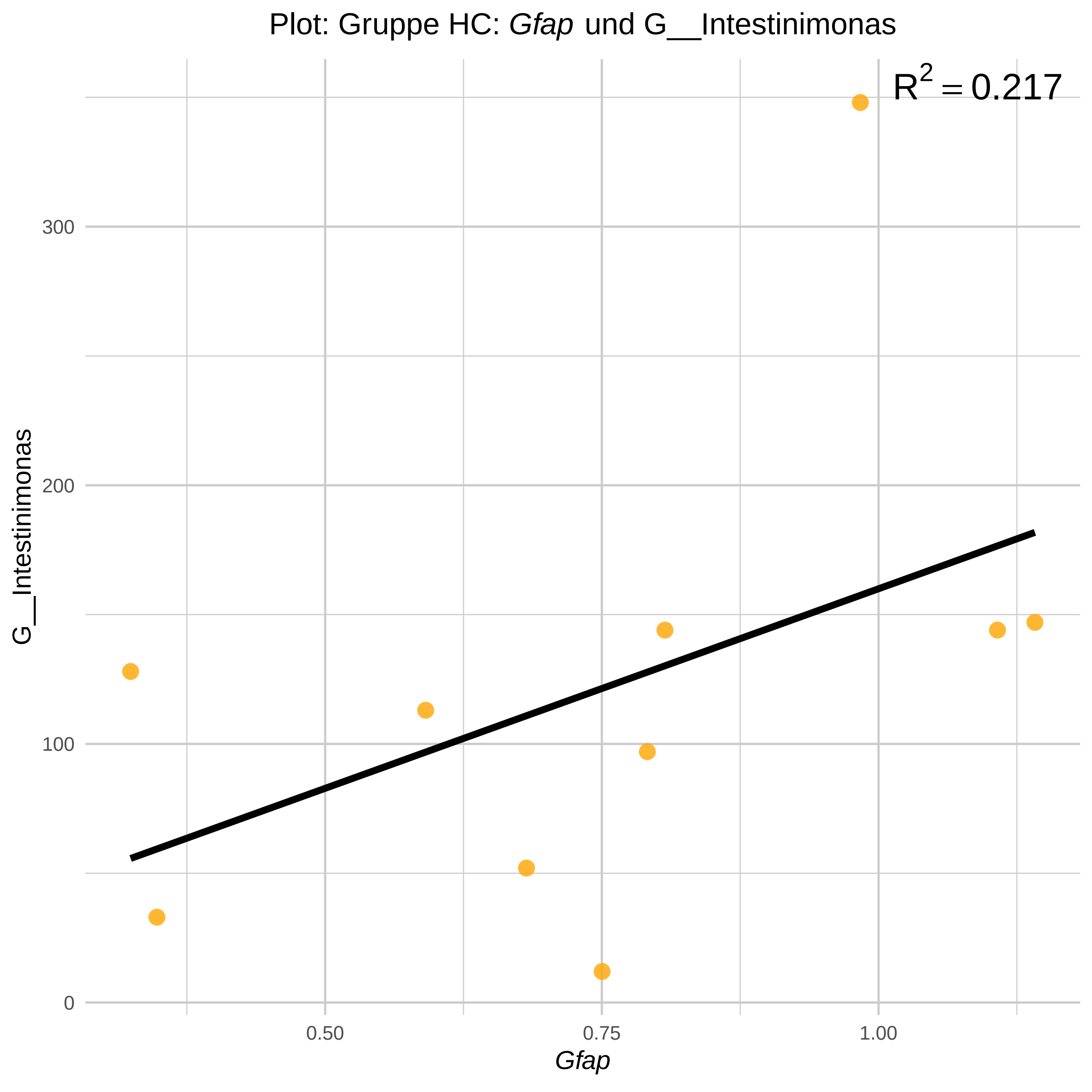

Plot: Gruppe HC: *Gfap* und G__Intestinimonas
$R^2 = 0.217$
G__Intestinimonas
300
200
100
0
0.50
0.75
1.00
Gfap

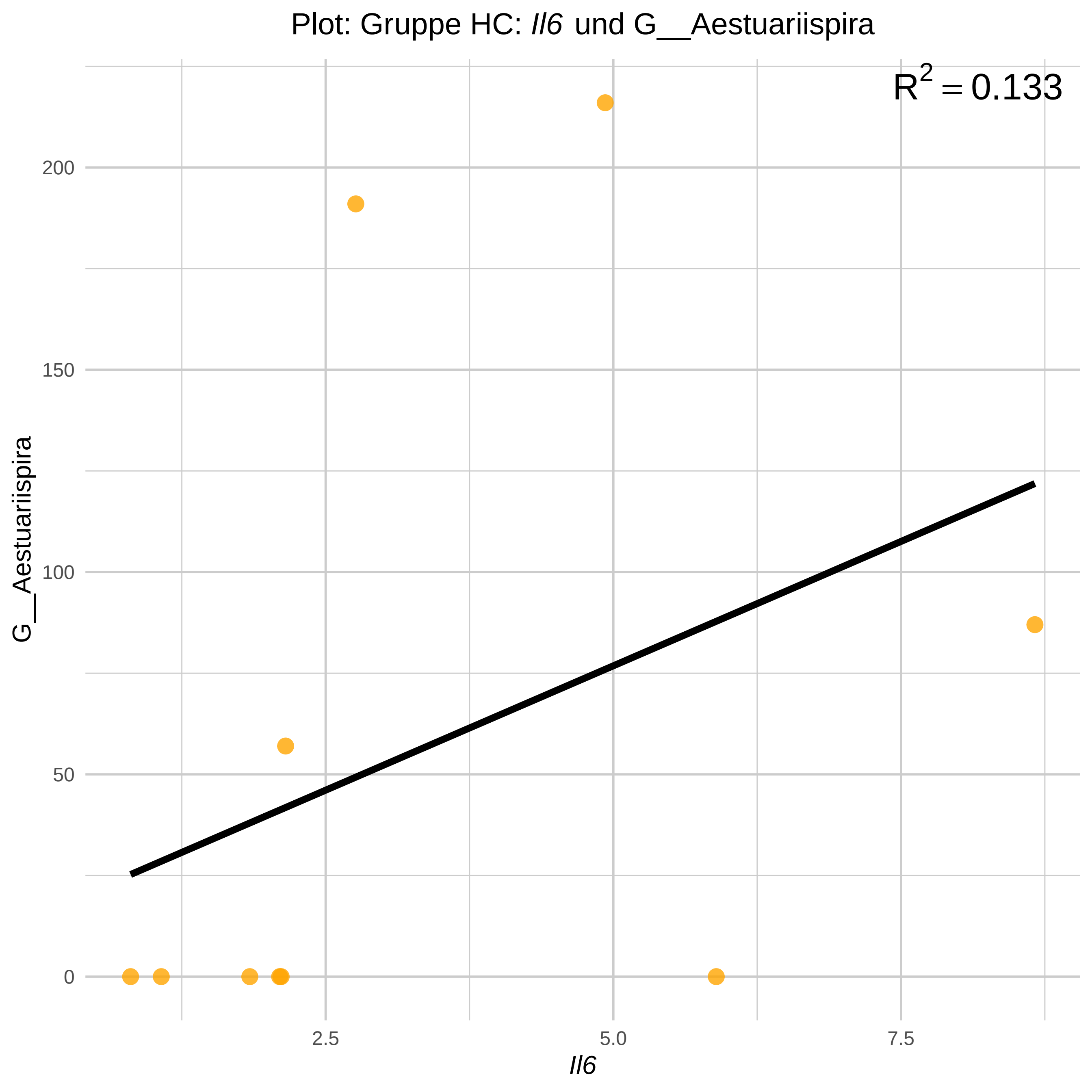

Plot: Gruppe HC: Il6 und G__Aestuariispira
$R^2 = 0.133$
200
150
100
50
0
G__Aestuariispira
2.5
5.0
7.5
Il6

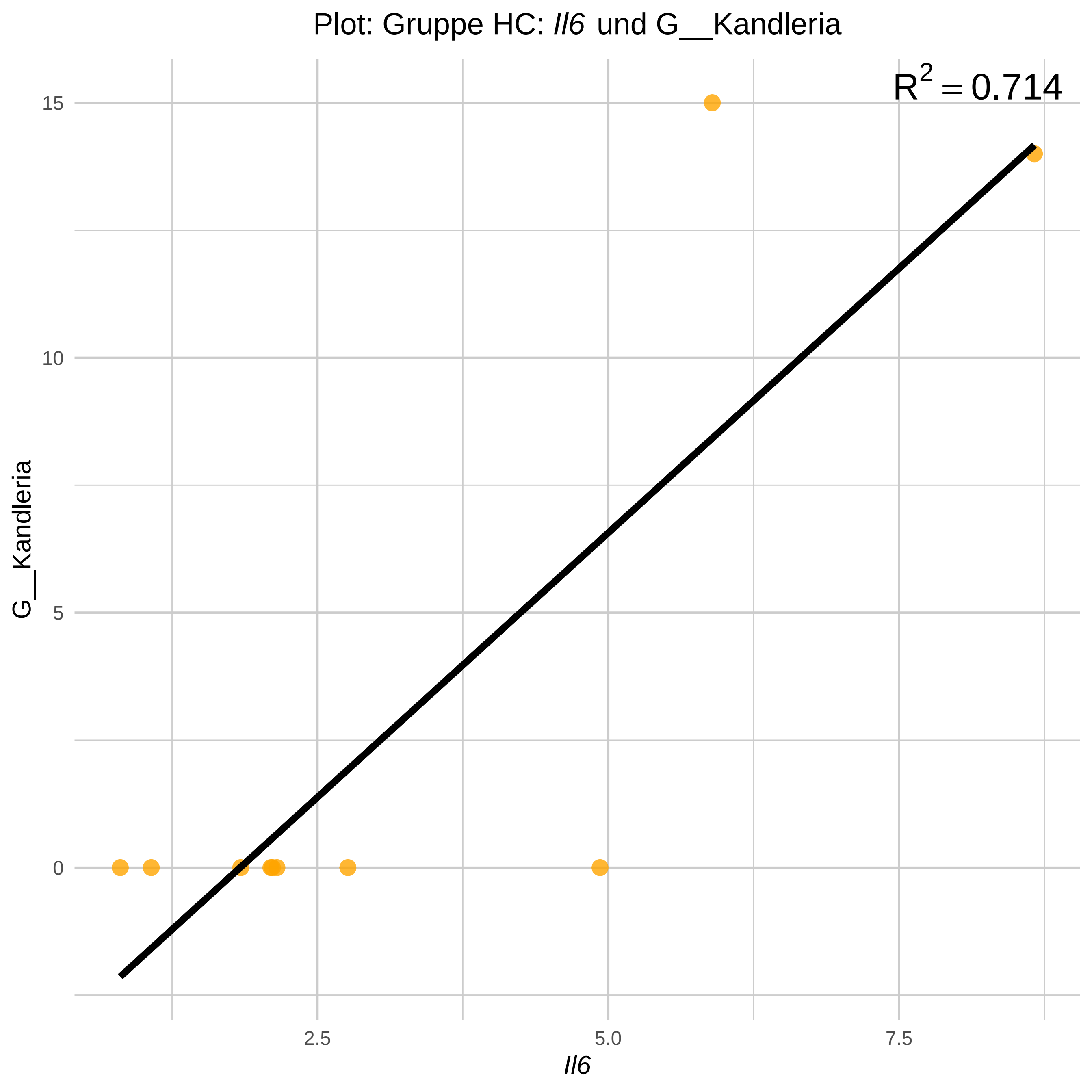

Plot: Gruppe HC: *Il6* und G__Kandleria
$R^2 = 0.714$
15
10
5
0
G__Kandleria
2.5
5.0
7.5
Il6

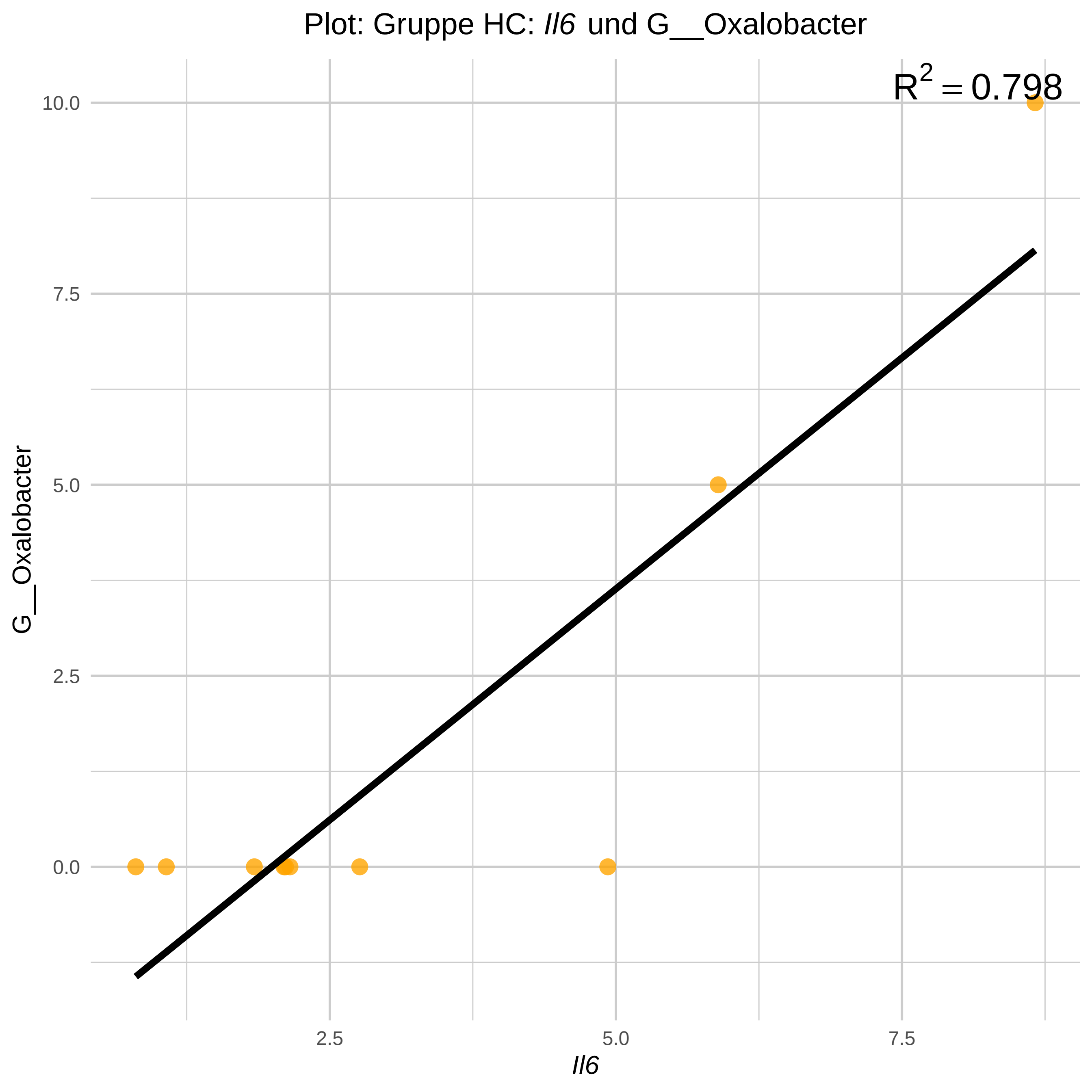
Plot: Gruppe HC: *Il6* und G__Oxalobacter
$R^2 = 0.798$
G__Oxalobacter
10.0
7.5
5.0
2.5
0.0
2.5
5.0
7.5
Il6

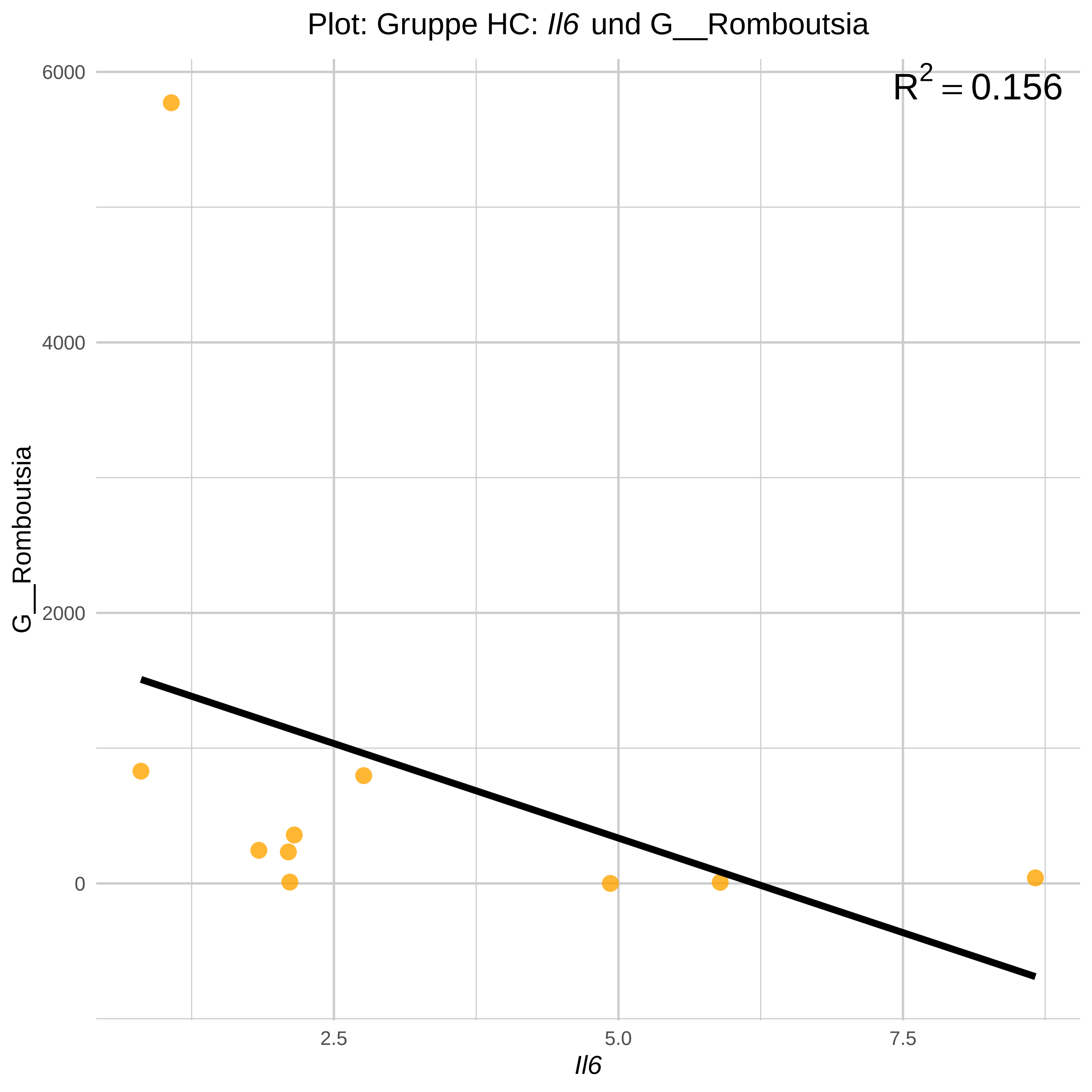
Plot: Gruppe HC: Il6 und G__Romboutsia
$R^2 = 0.156$
6000
4000
2000
0
G__Romboutsia
2.5
5.0
7.5
Il6

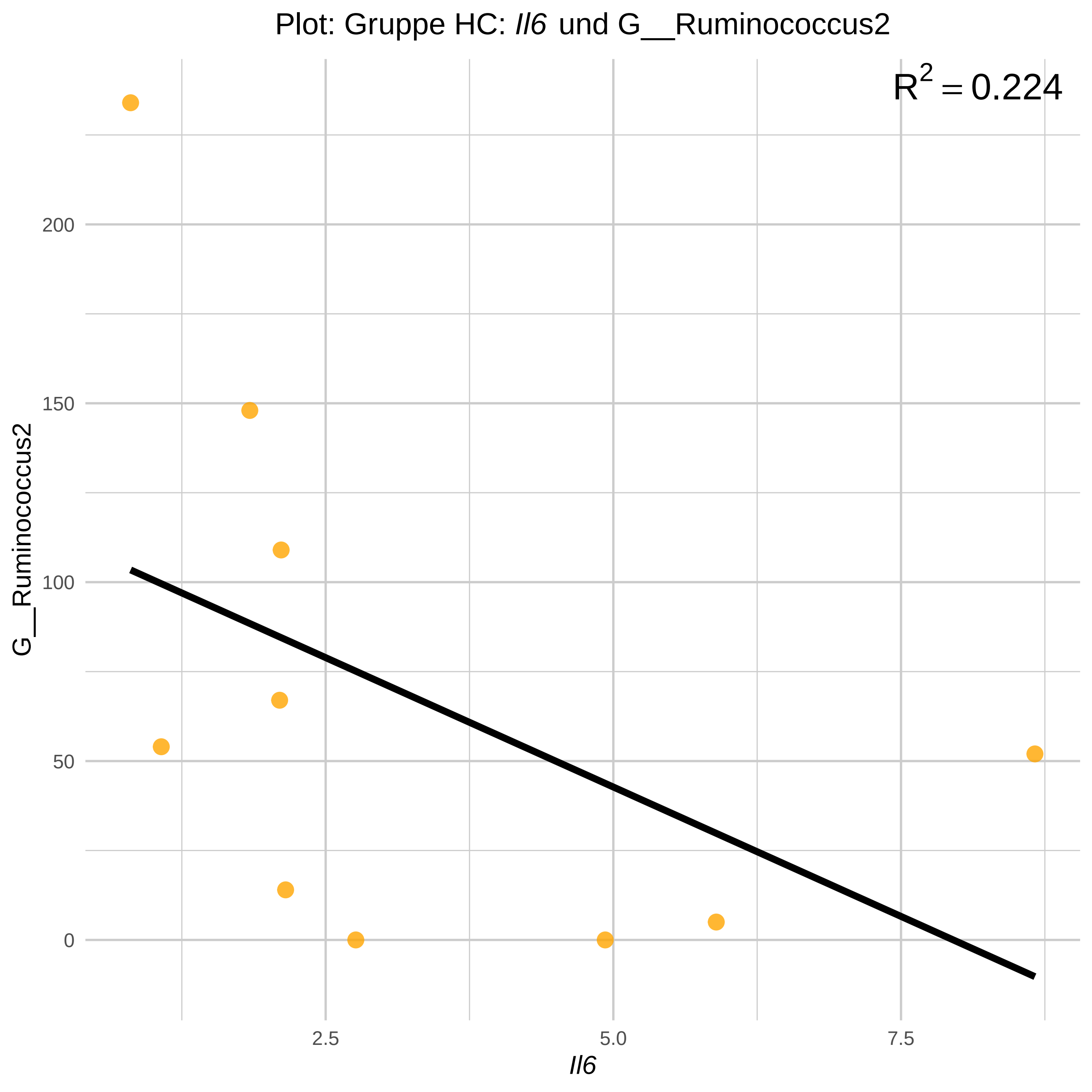
Plot: Gruppe HC: Il6 und G__Ruminococcus2
R2 = 0.224
200
150
100
50
0
G__Ruminococcus2
2.5
5.0
7.5
Il6

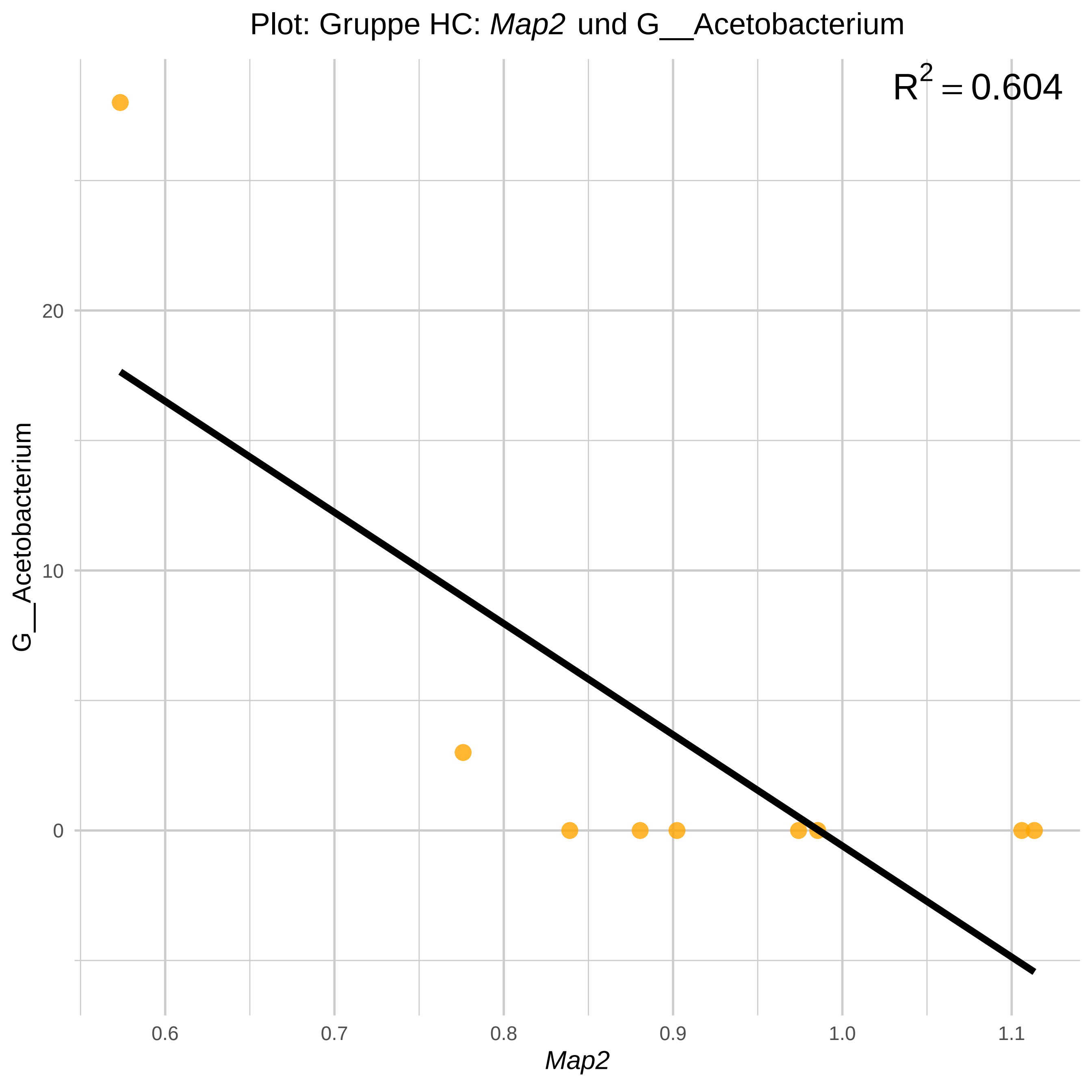

Plot: Gruppe HC: *Map2* und G__Acetobacterium
$R^2 = 0.604$
G__Acetobacterium
20
10
0
0.6
0.7
0.8
0.9
1.0
1.1
Map2

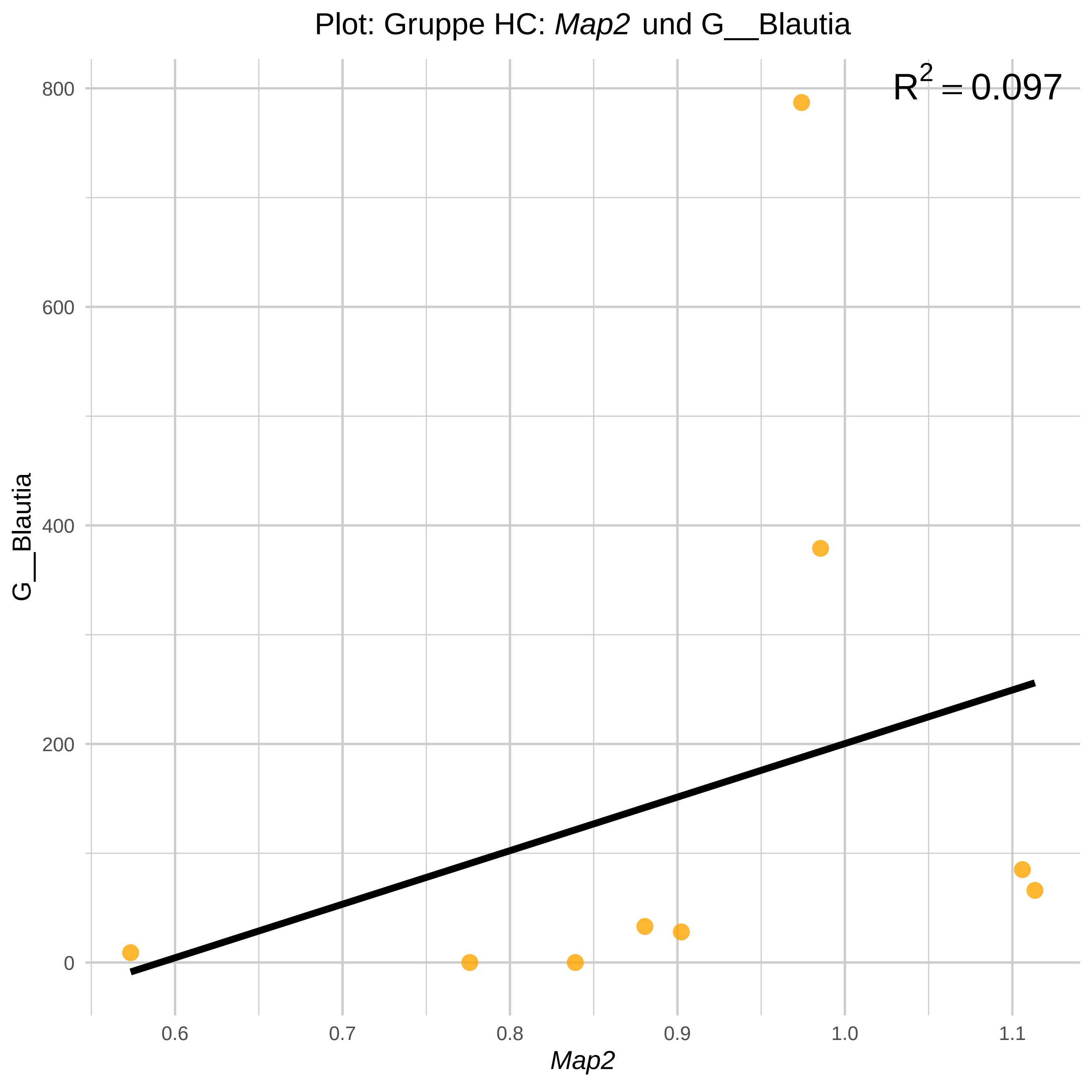

Plot: Gruppe HC: *Map2* und G__Blautia
$R^2 = 0.097$
800
600
400
200
0
G__Blautia
0.6
0.7
0.8
0.9
1.0
1.1
Map2

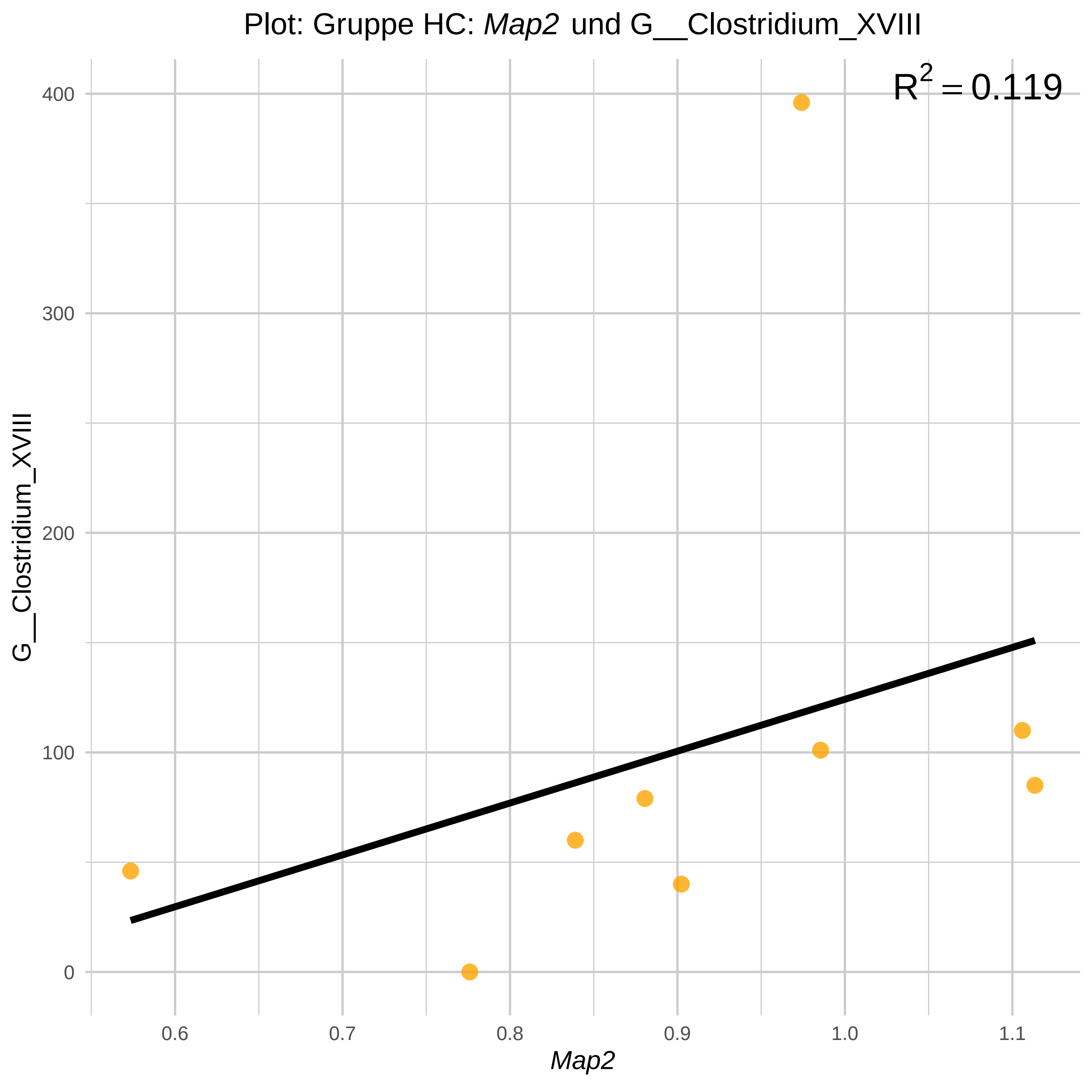

Plot: Gruppe HC: *Map2* und G__Clostridium_XVIII
$R^2 = 0.119$
400
300
200
100
0
G__Clostridium_XVIII
0.6
0.7
0.8
0.9
1.0
1.1
Map2

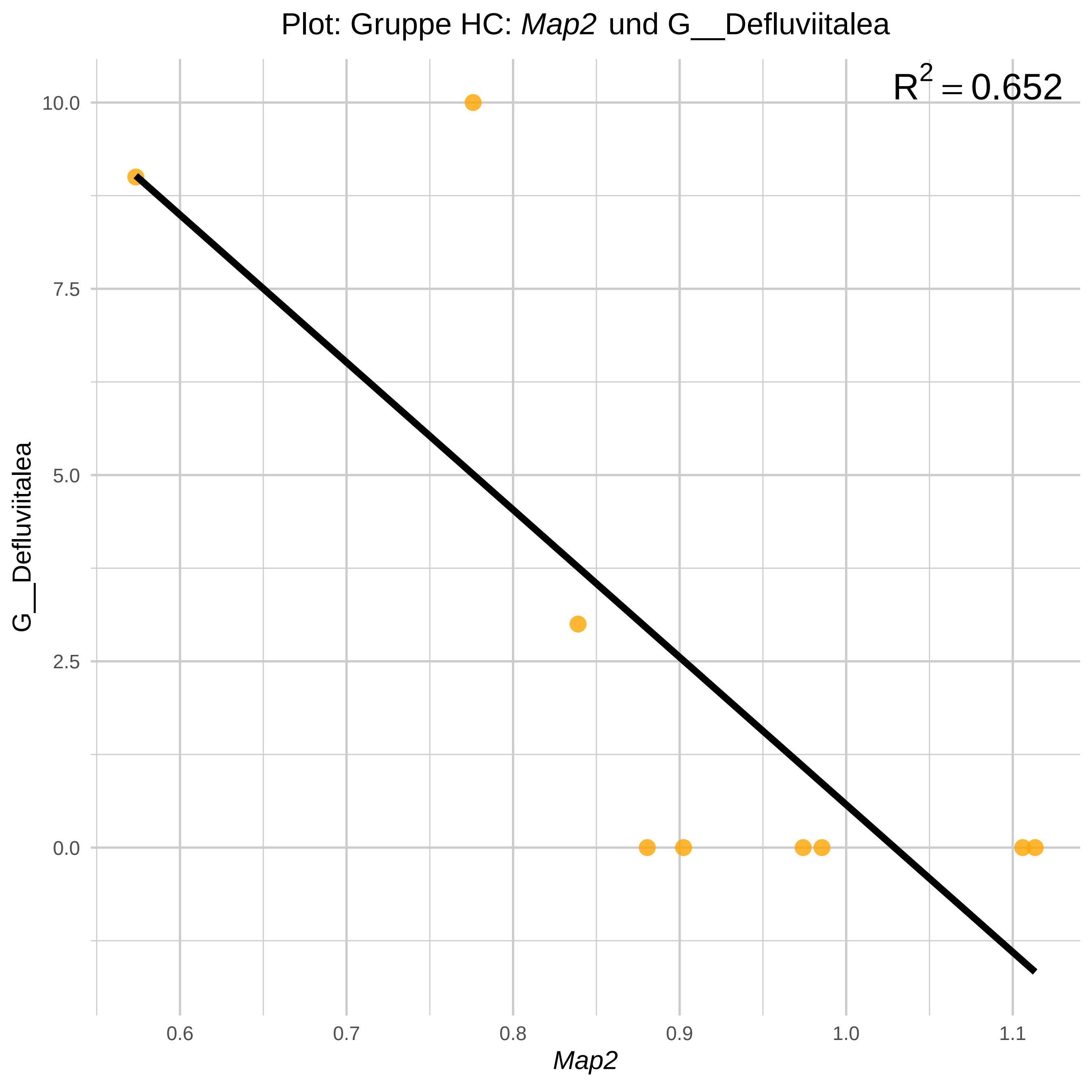
Plot: Gruppe HC: *Map2* und G__Defluviitalea
$R^2 = 0.652$
10.0
7.5
5.0
2.5
0.0
G__Defluviitalea
0.6
0.7
0.8
0.9
1.0
1.1
Map2

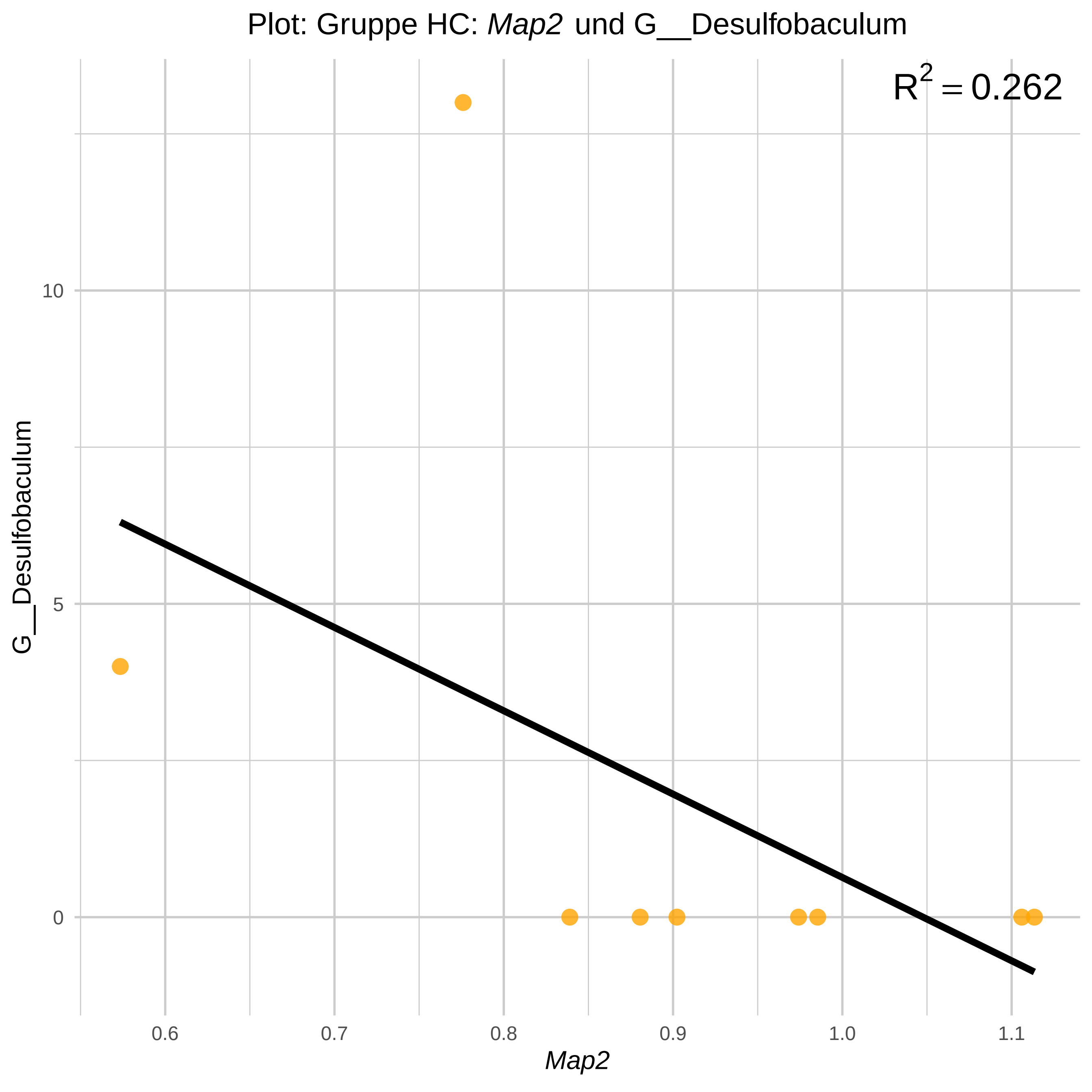
Plot: Gruppe HC: *Map2* und G__Desulfobaculum
$R^2 = 0.262$
G__Desulfobaculum
10
5
0
0.6
0.7
0.8
0.9
1.0
1.1
Map2

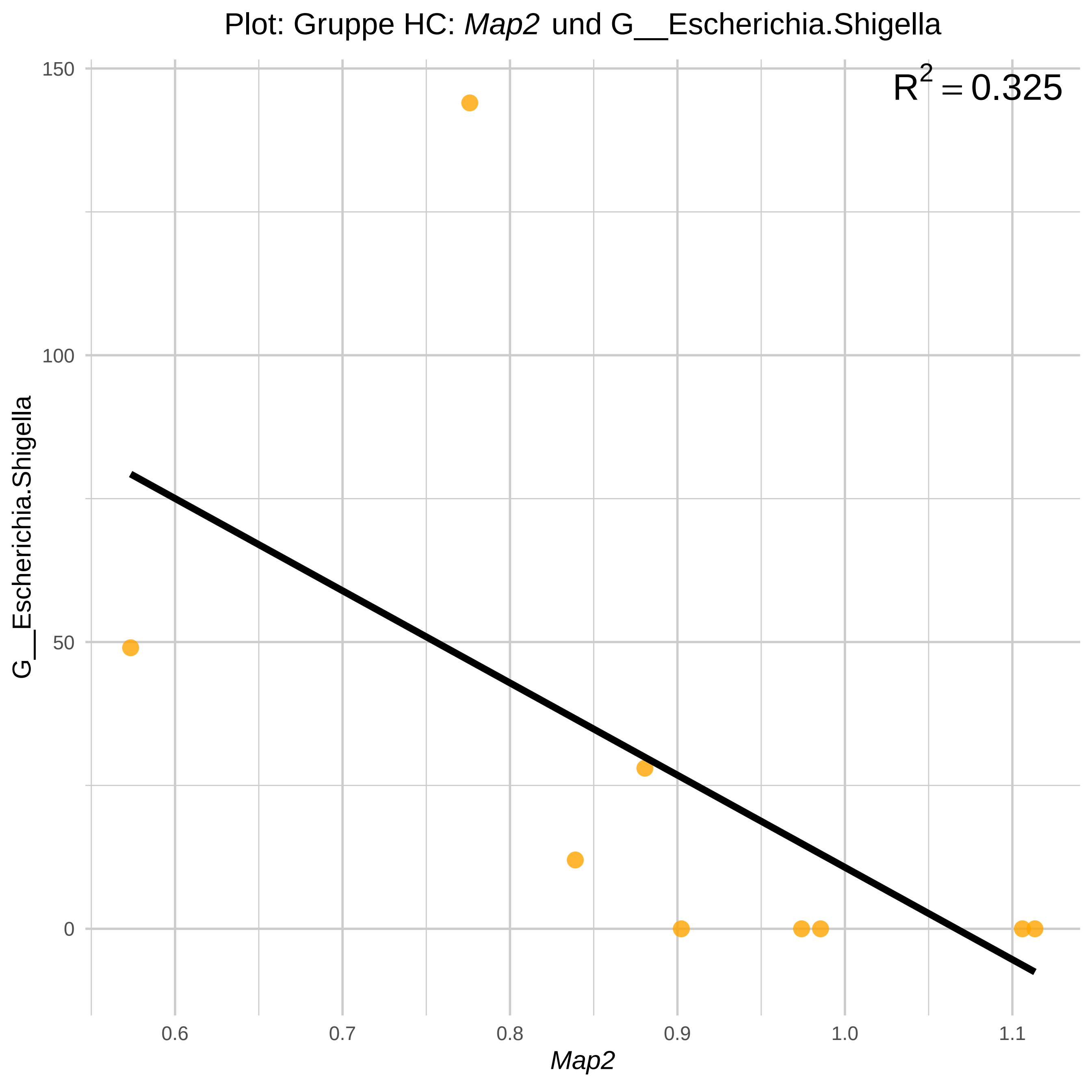

Plot: Gruppe HC: Map2 und G__Escherichia.Shigella
R2 = 0.325
150
100
50
0
0.6
0.7
0.8
0.9
1.0
1.1
Map2
G__Escherichia.Shigella

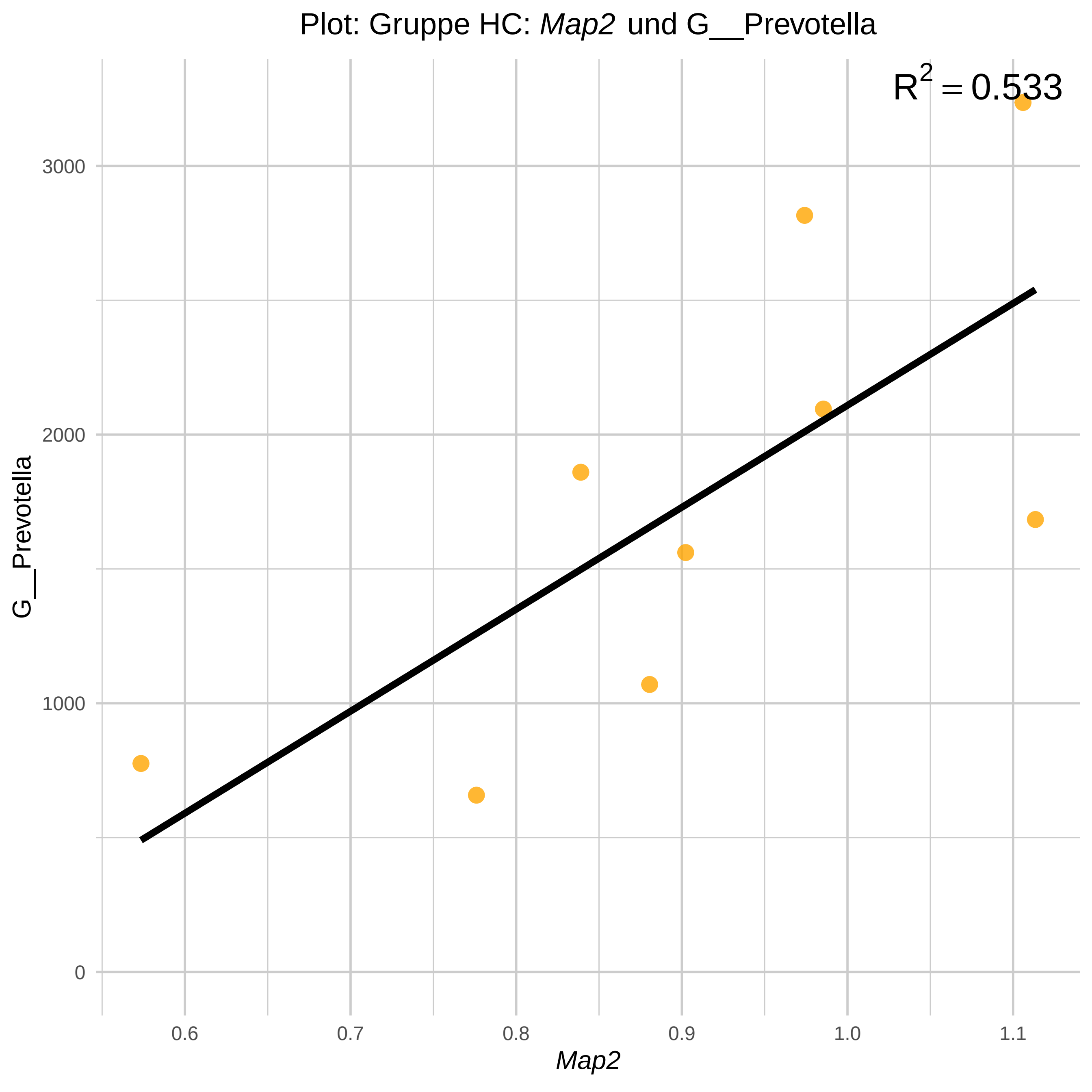
Plot: Gruppe HC: Map2 und G__Prevotella
R2 = 0.533
G__Prevotella
3000
2000
1000
0
0.6
0.7
0.8
0.9
1.0
1.1
Map2

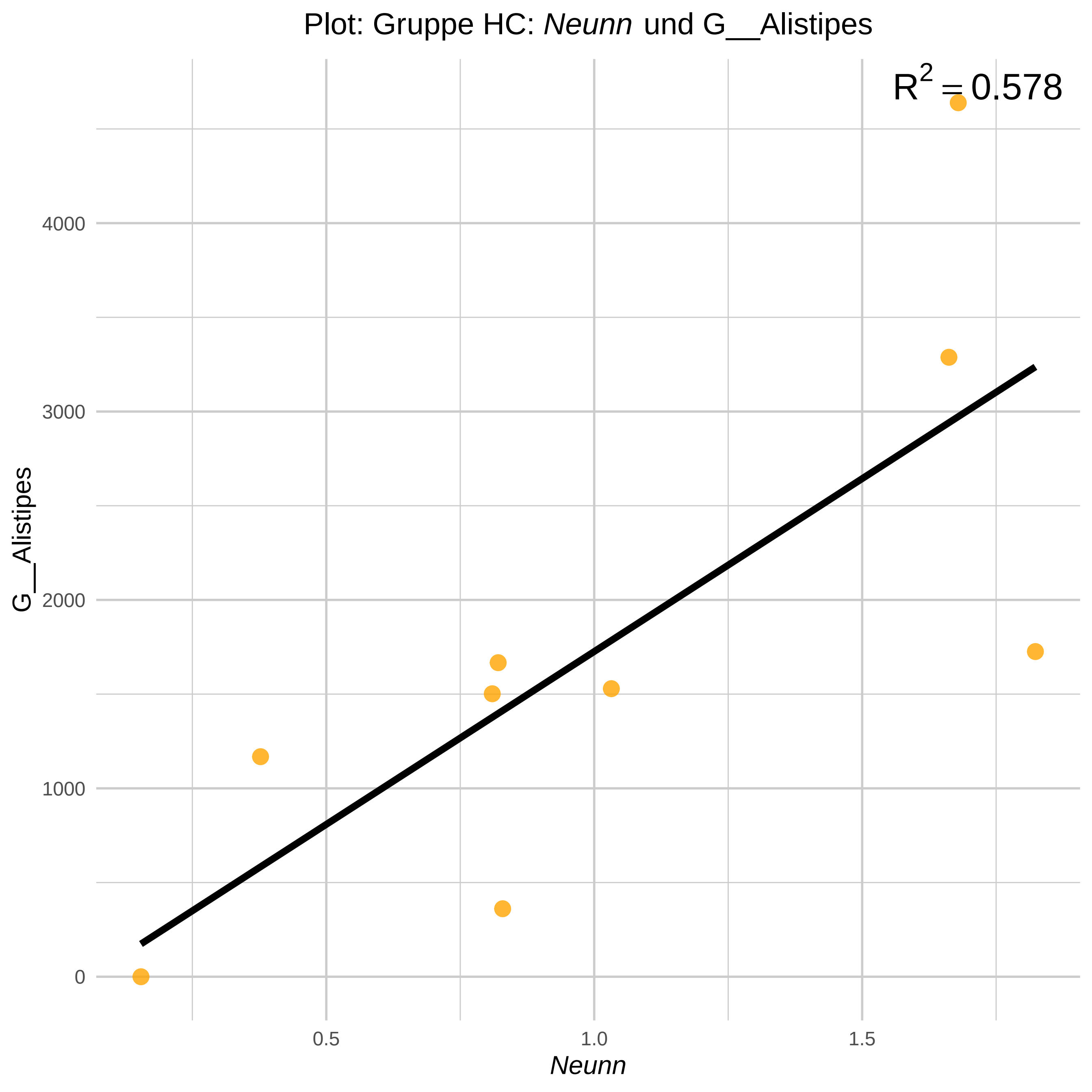

Plot: Gruppe HC: Neunn und G__Alistipes
$R^2 = 0.578$
4000
3000
2000
1000
0
G__Alistipes
0.5
1.0
1.5
Neunn

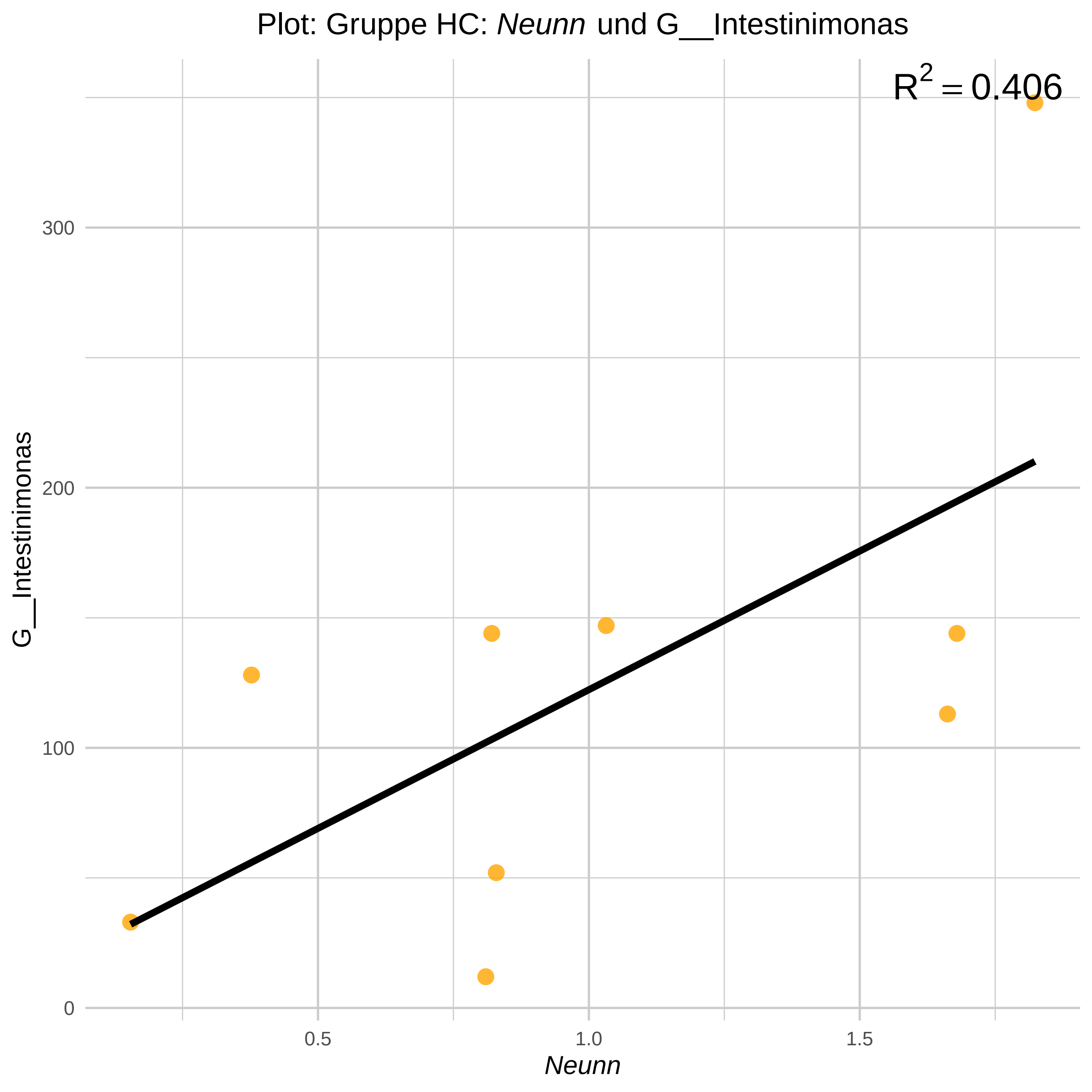

Plot: Gruppe HC: *Neunn* und G__Intestinimonas
$R^2 = 0.406$
300
200
100
0
0.5
1.0
1.5
Neunn
G__Intestinimonas

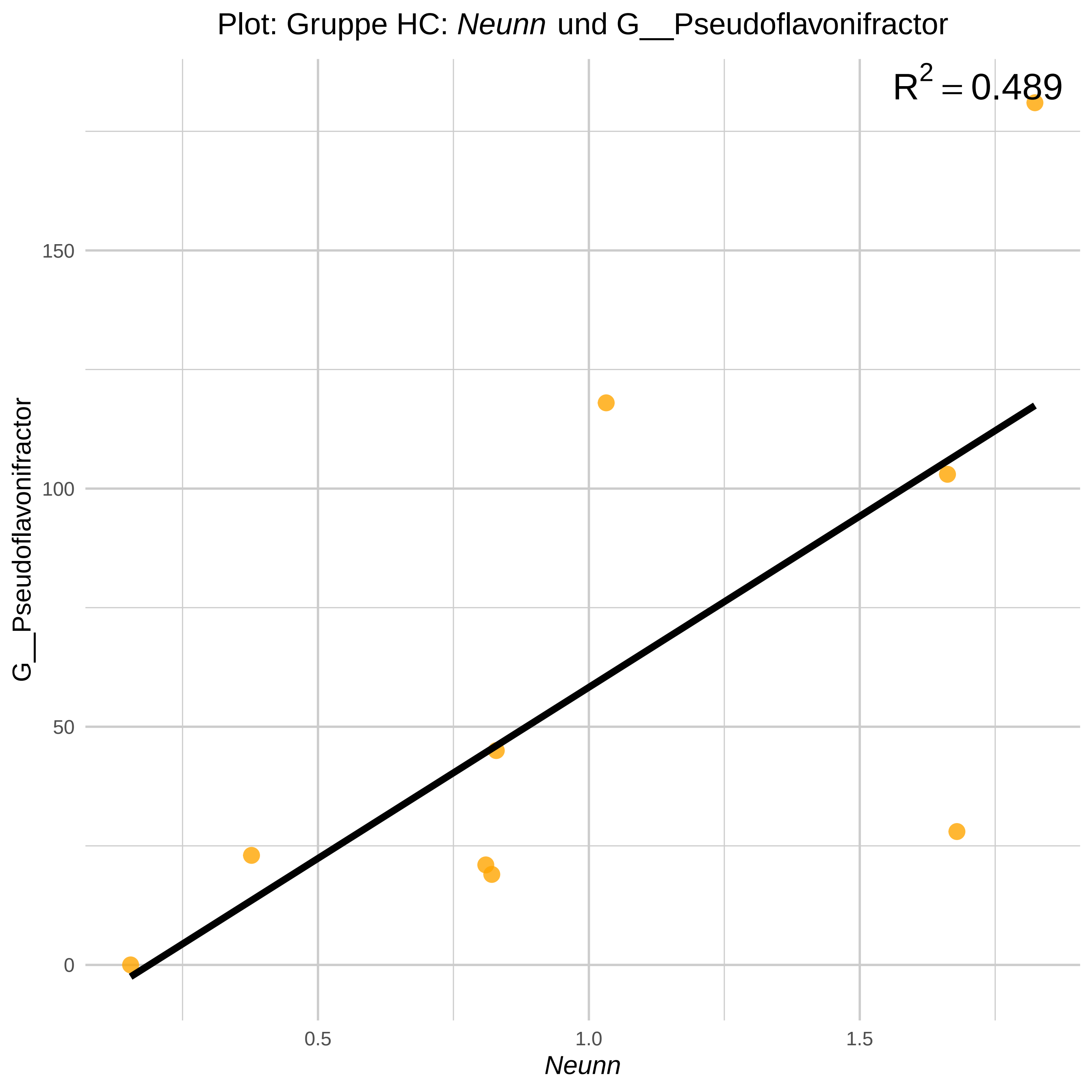

Plot: Gruppe HC: *Neunn* und G__Pseudoflavonifractor
$R^2 = 0.489$
150
100
50
0
0.5
1.0
1.5
Neunn
G__Pseudoflavonifractor

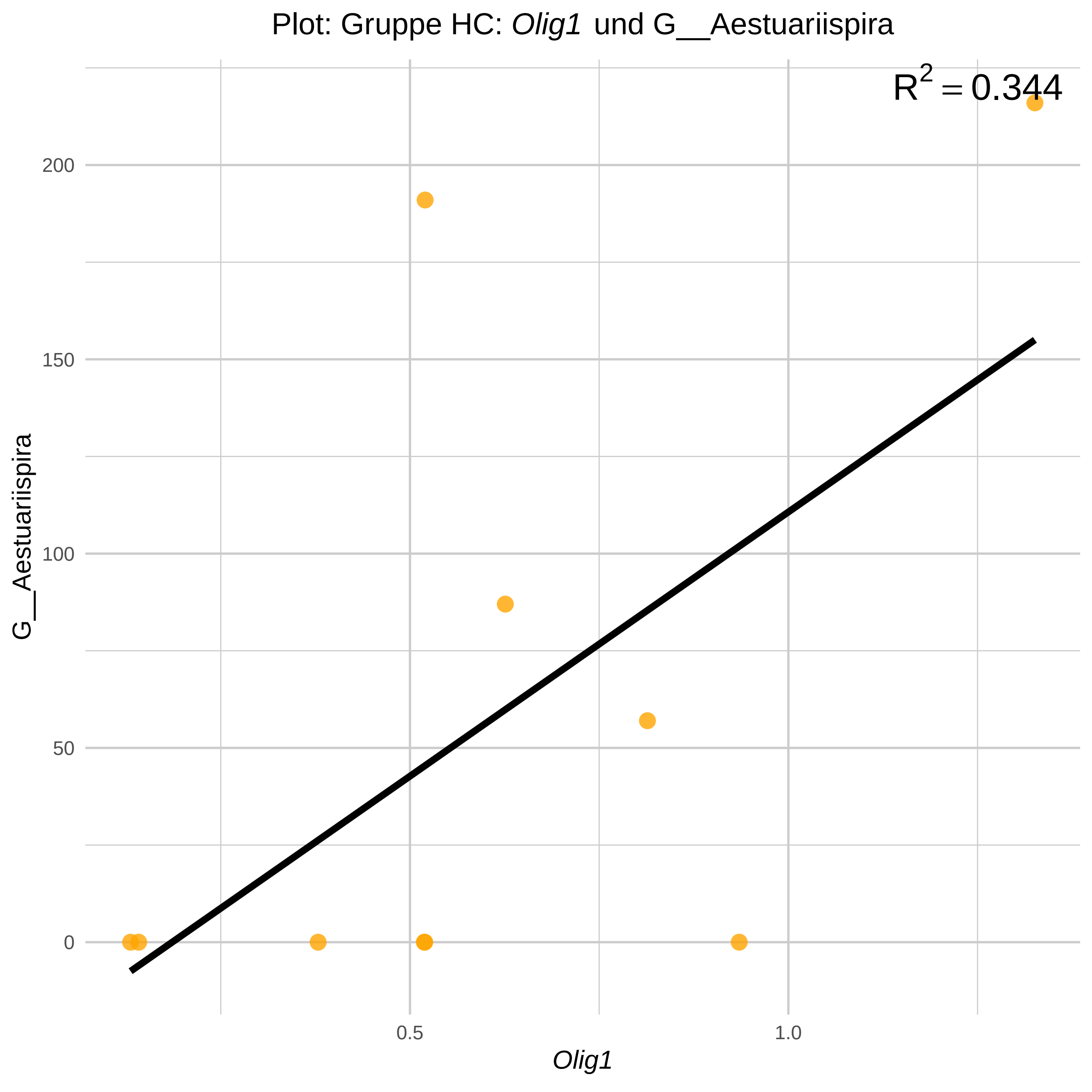

Plot: Gruppe HC: Olig1 und G__Aestuariispira
R2 = 0.344
200
150
100
50
0
0.5
1.0
Olig1
G__Aestuariispira

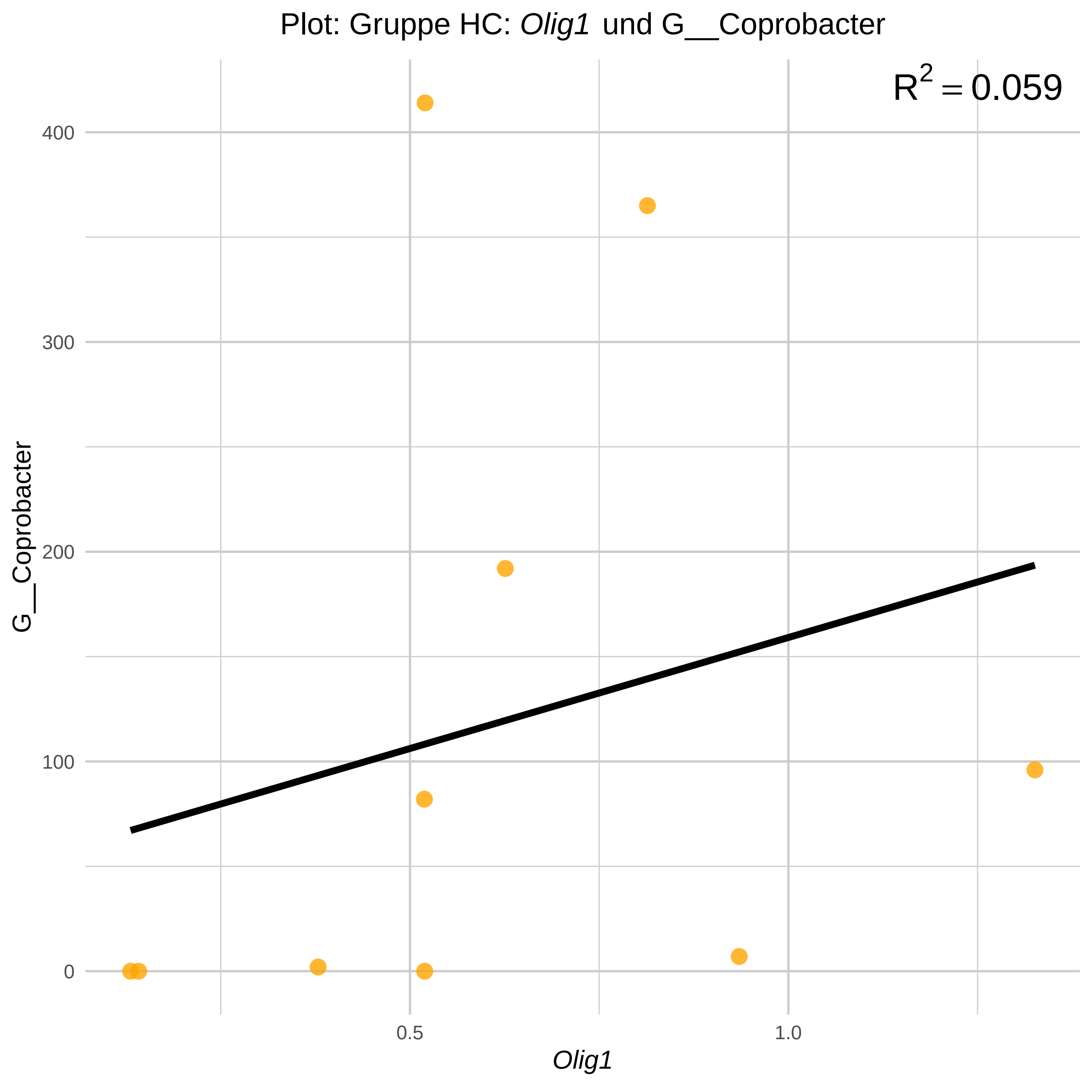

Plot: Gruppe HC: Olig1 und G__Coprobacter
R2 = 0.059
400
300
200
100
0
0.5
1.0
Olig1
G__Coprobacter

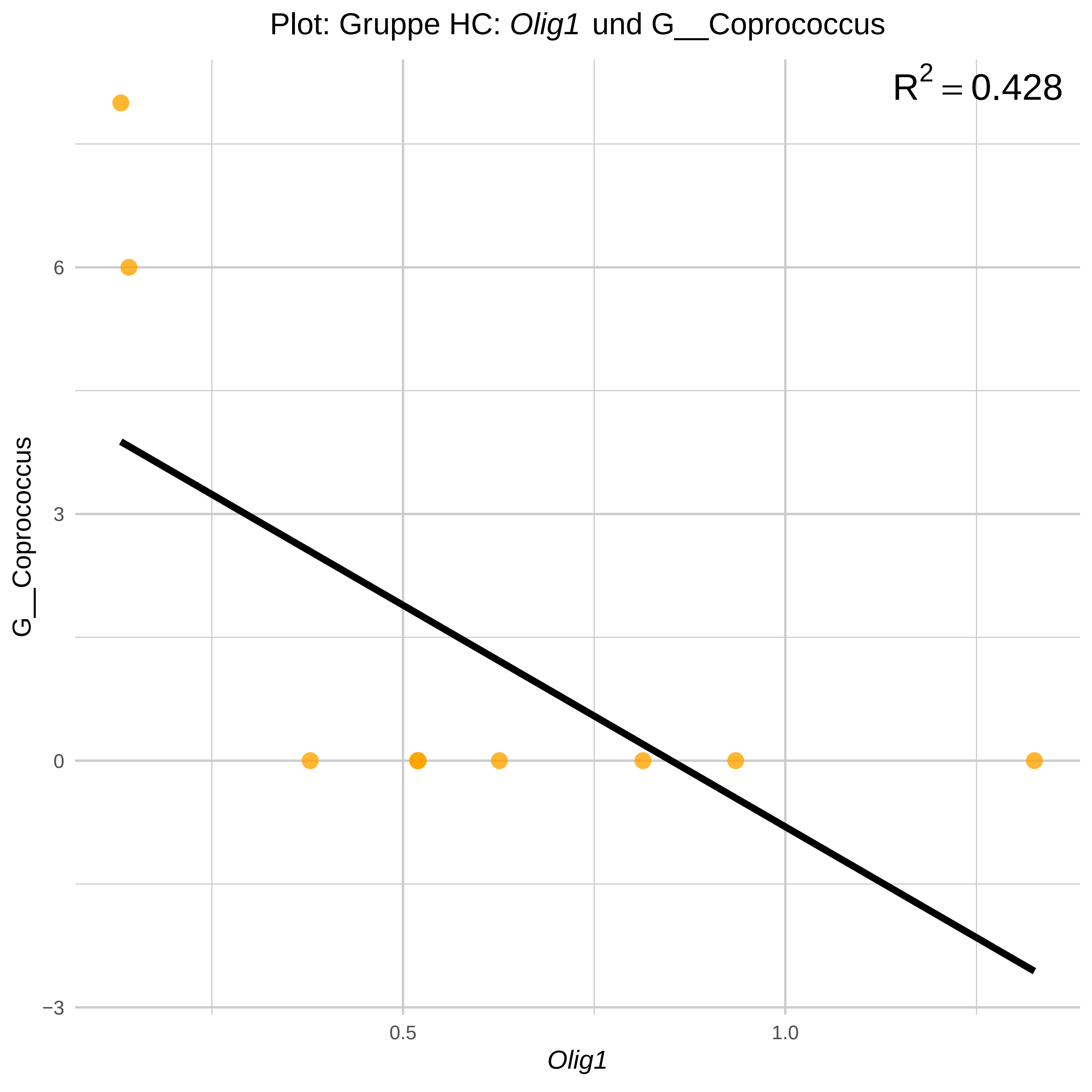

Plot: Gruppe HC: *Olig1* und G__Coprococcus
$R^2 = 0.428$
6
3
0
−3
0.5
1.0
Olig1
G__Coprococcus

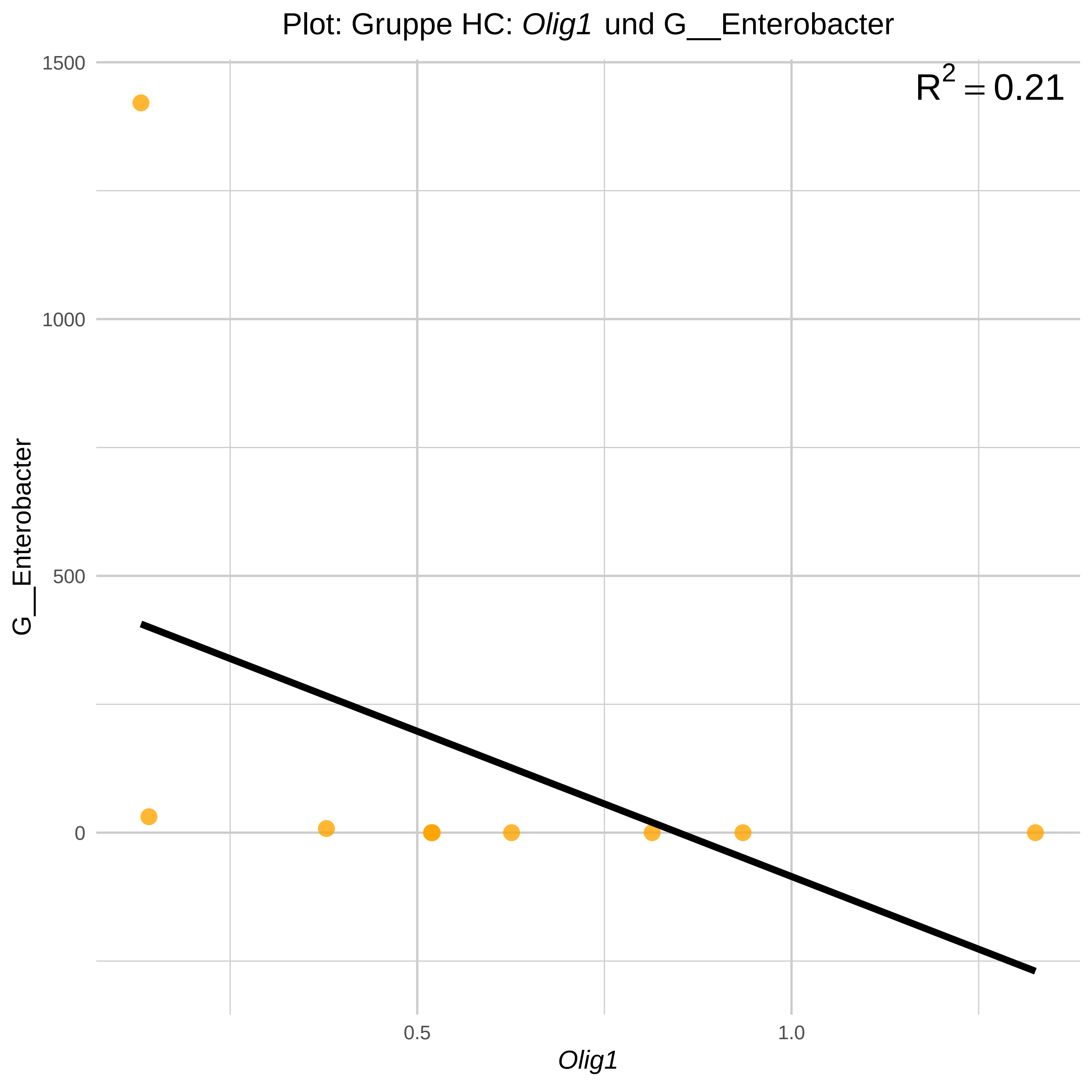

Plot: Gruppe HC: *Olig1* und G__Enterobacter
$R^2 = 0.21$
1500
1000
500
0
G__Enterobacter
0.5
1.0
Olig1

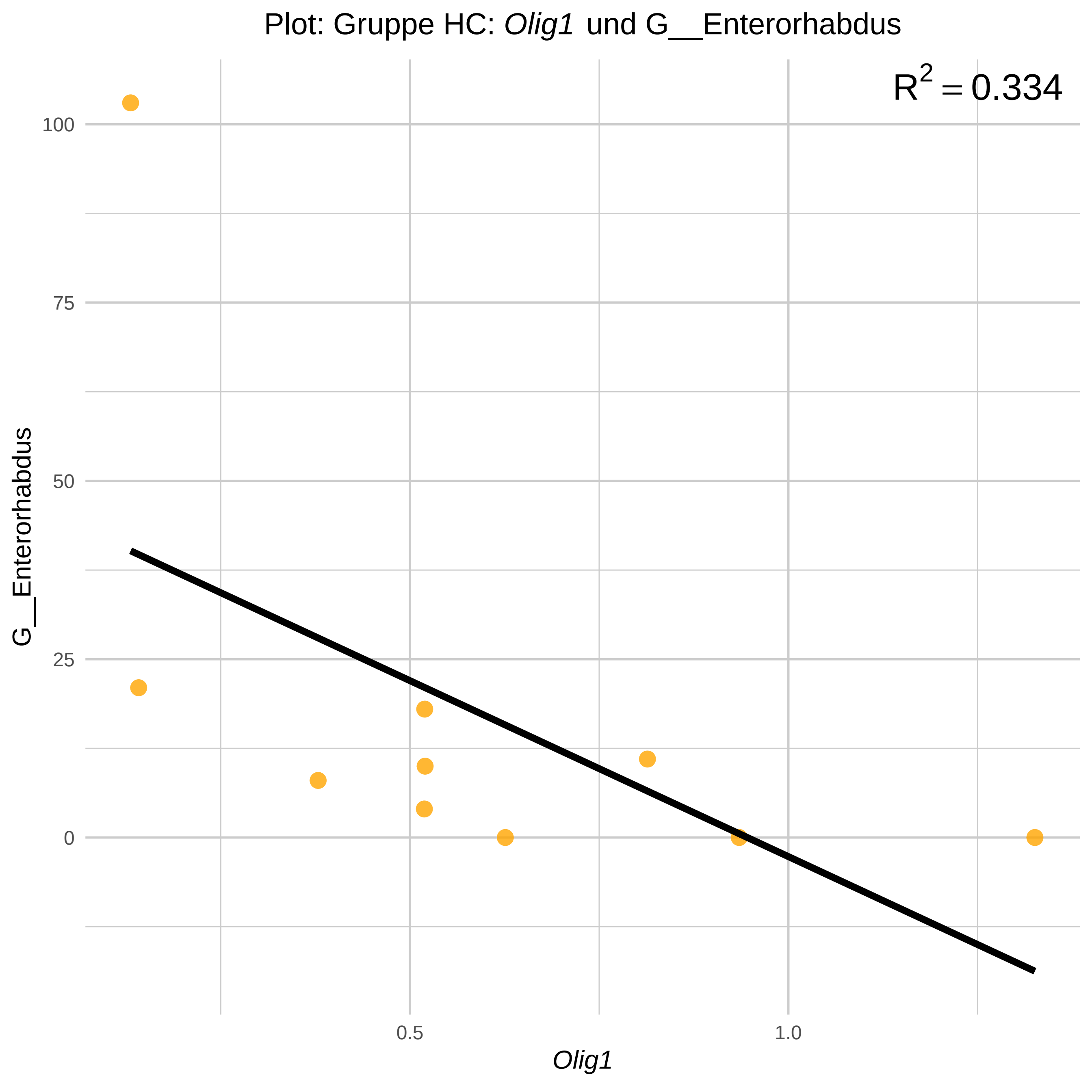

Plot: Gruppe HC: *Olig1* und G__Enterorhabdus
$R^2 = 0.334$
100
75
50
25
0
G__Enterorhabdus
0.5
1.0
Olig1

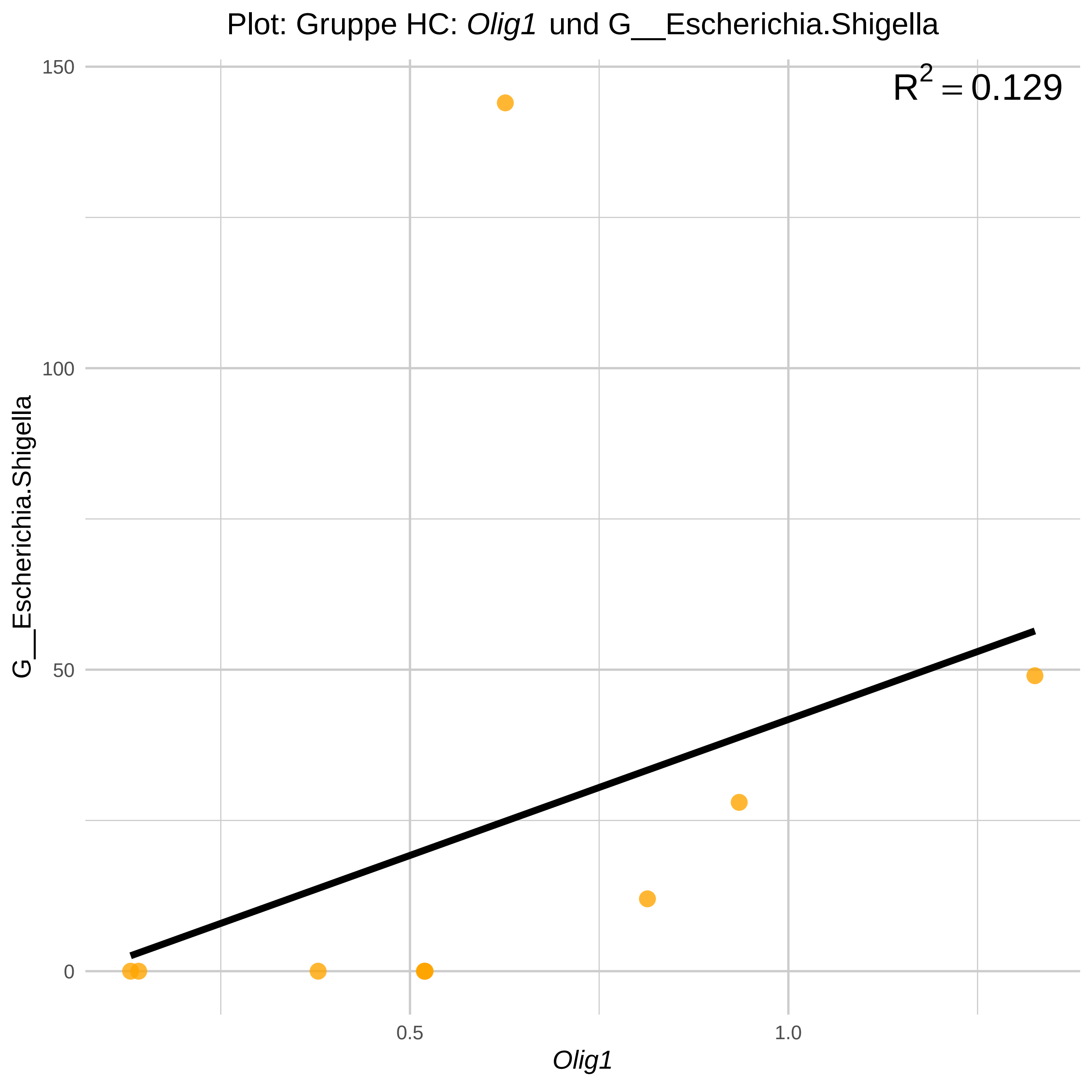

Plot: Gruppe HC: Olig1 und G__Escherichia.Shigella
$R^2 = 0.129$
150
100
50
0
0.5
1.0
Olig1
G__Escherichia.Shigella

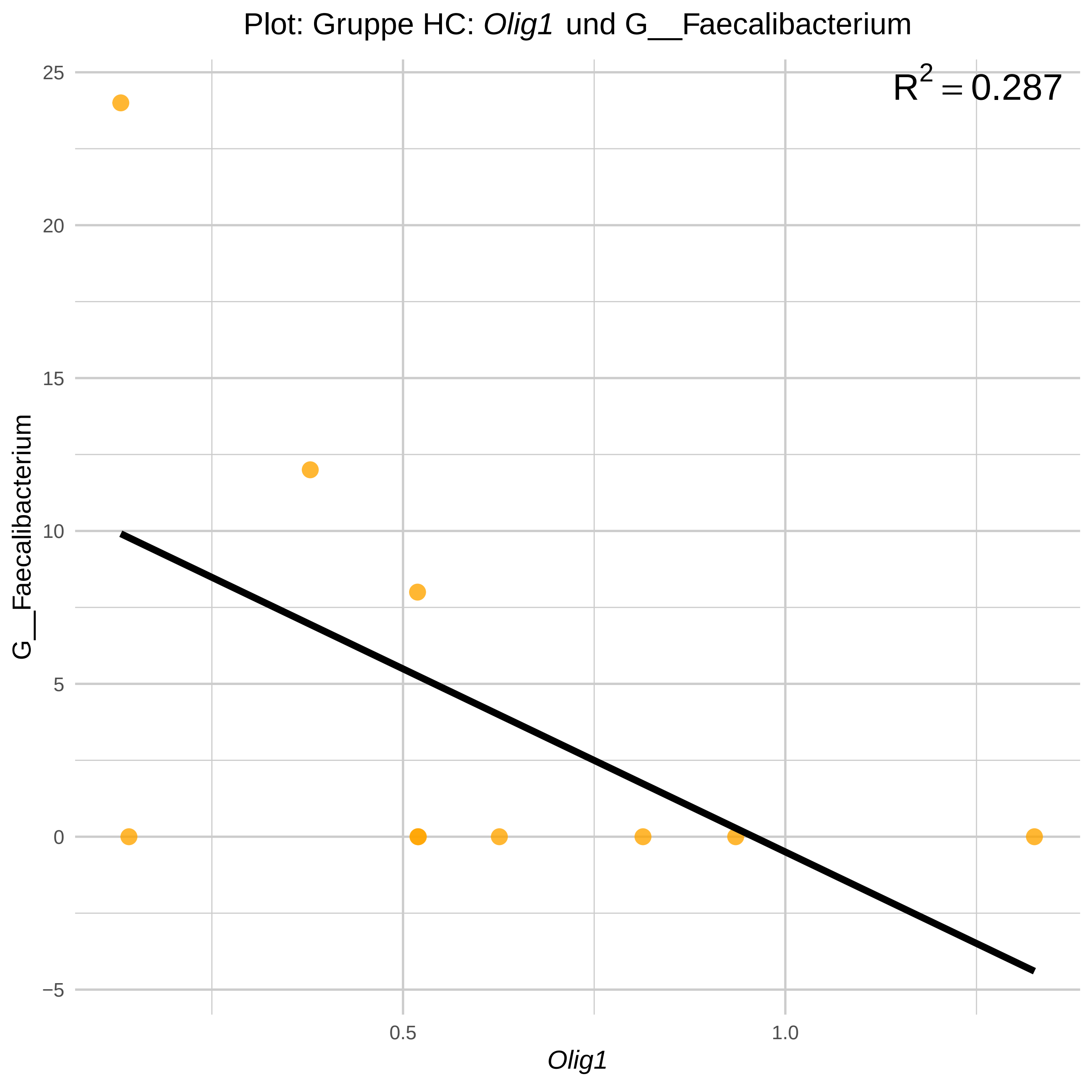

Plot: Gruppe HC: Olig1 und G__Faecalibacterium
R2 = 0.287
25
20
15
10
5
0
−5
G__Faecalibacterium
0.5
1.0
Olig1

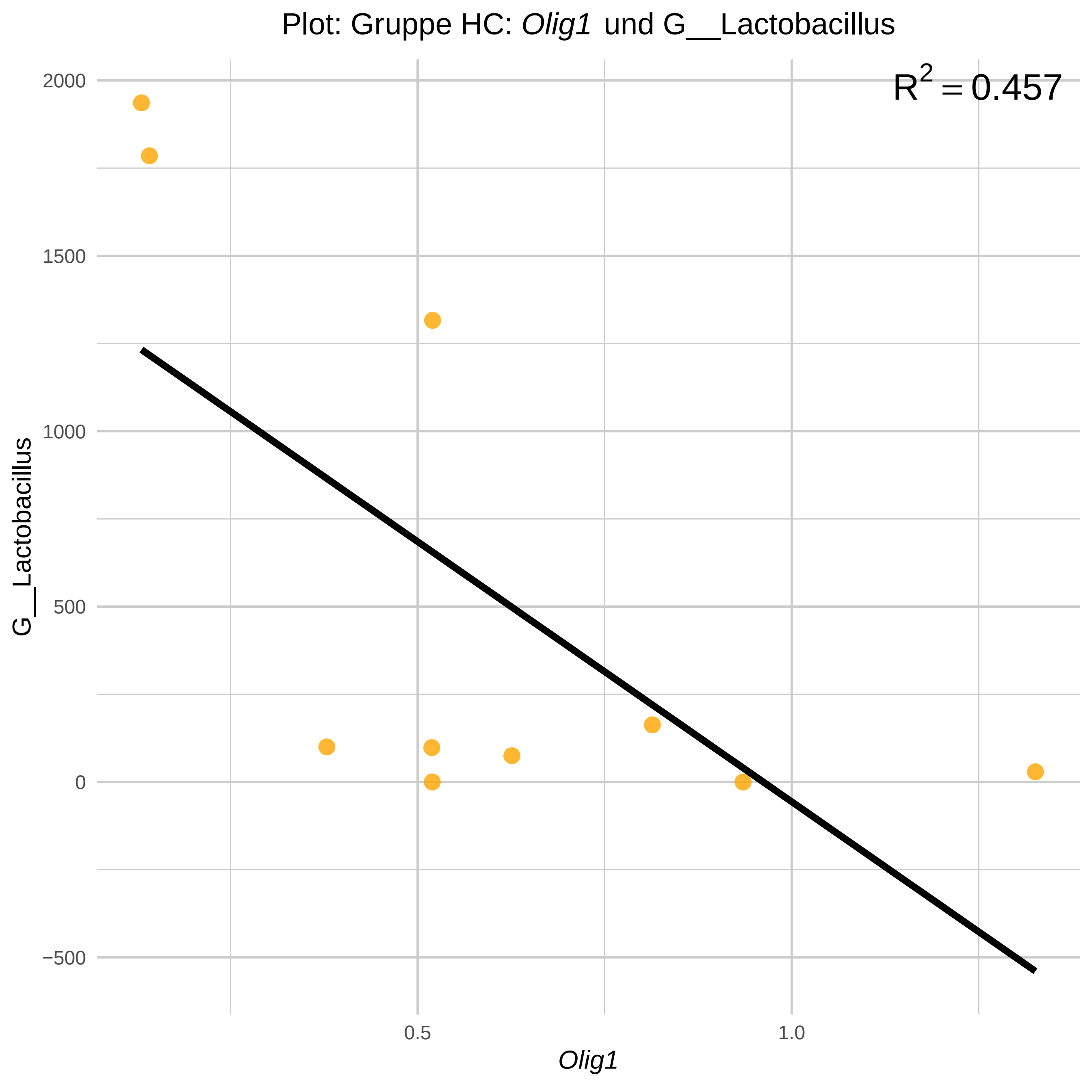

Plot: Gruppe HC: *Olig1* und G__Lactobacillus
$R^2 = 0.457$
2000
1500
1000
500
0
−500
G__Lactobacillus
0.5
1.0
Olig1

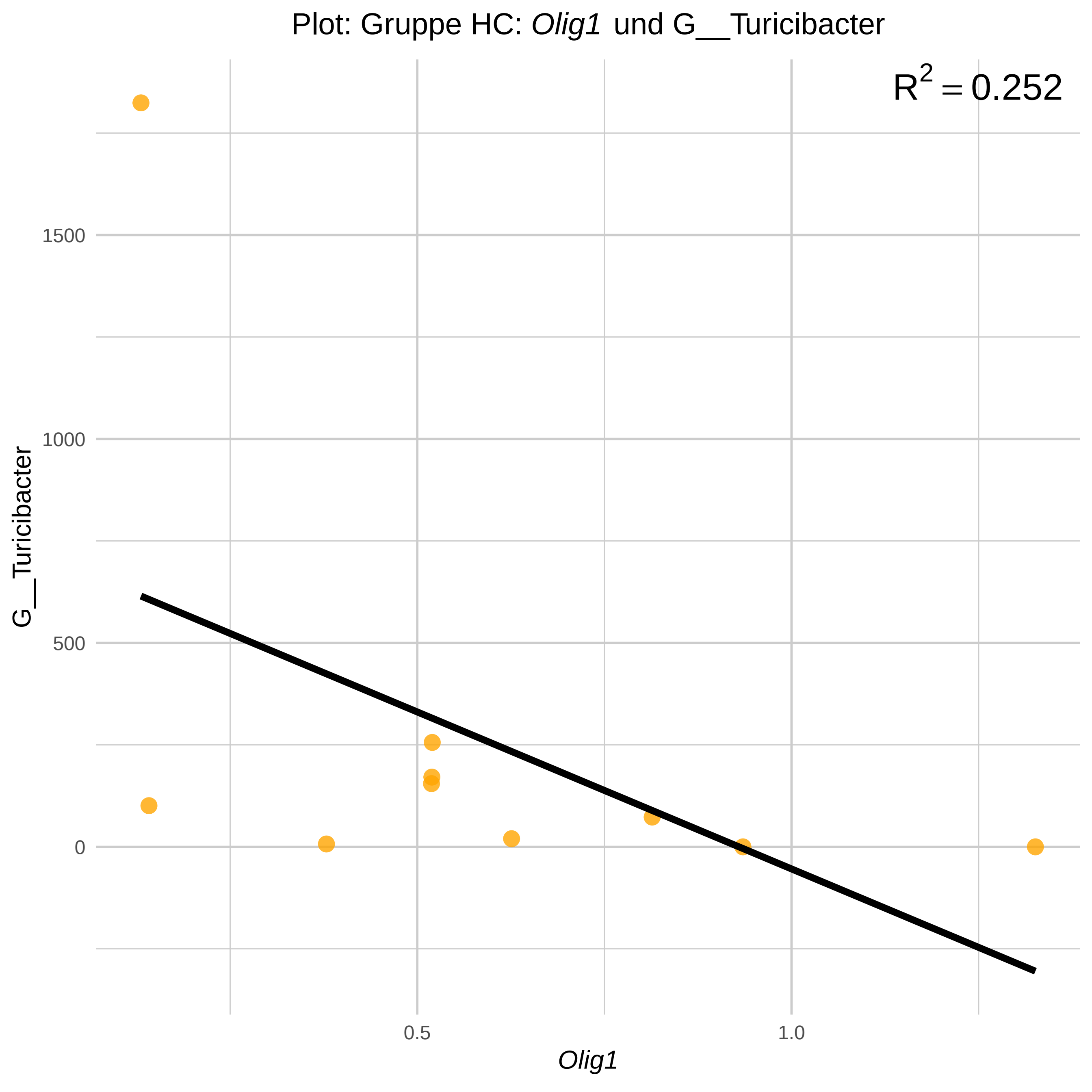

Plot: Gruppe HC: *Olig1* und G__Turicibacter
$R^2 = 0.252$
G__Turicibacter
1500
1000
500
0
0.5
1.0
Olig1

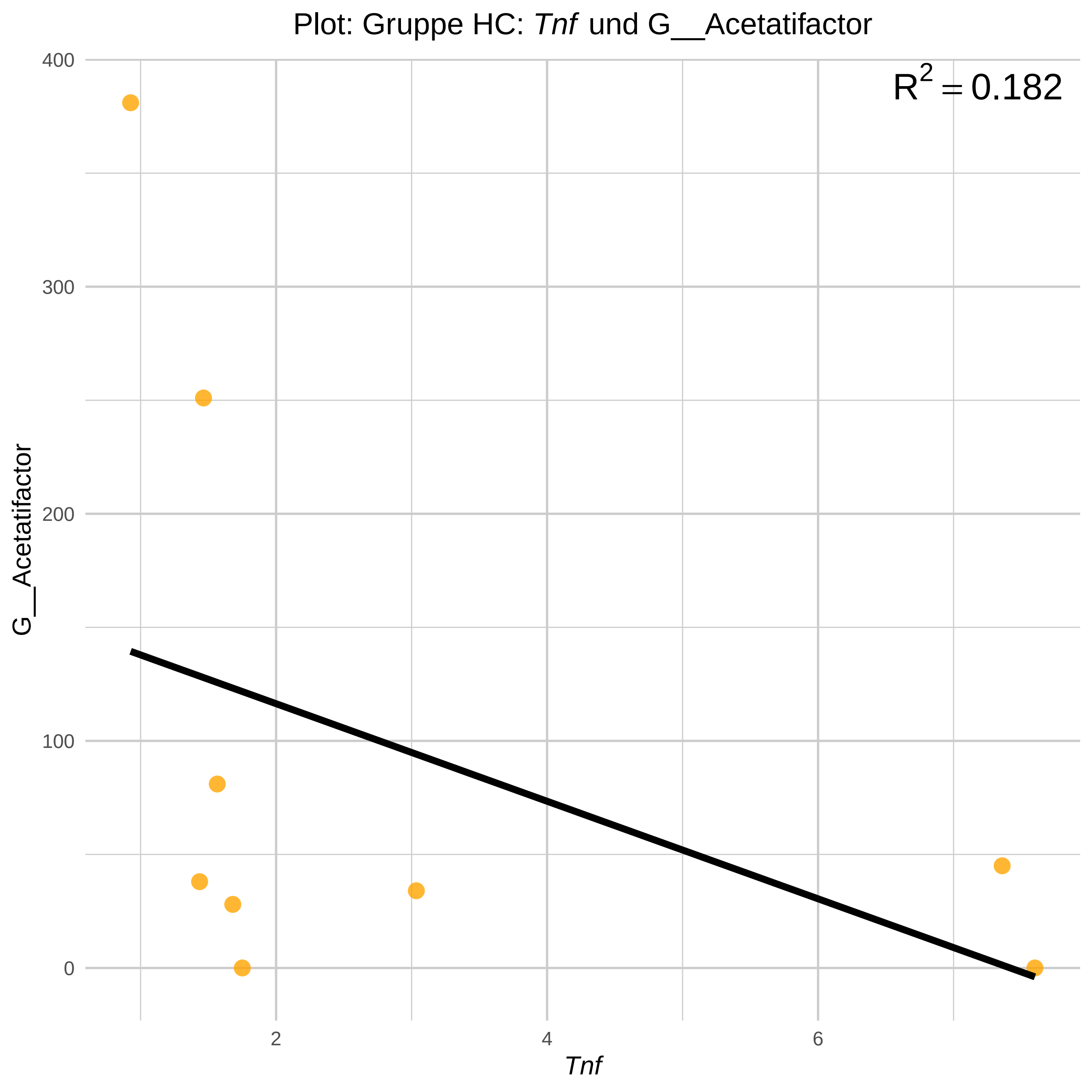

Plot: Gruppe HC: *Tnf* und G__Acetatifactor
$R^2 = 0.182$
400
300
200
100
0
G__Acetatifactor
2
4
6
Tnf

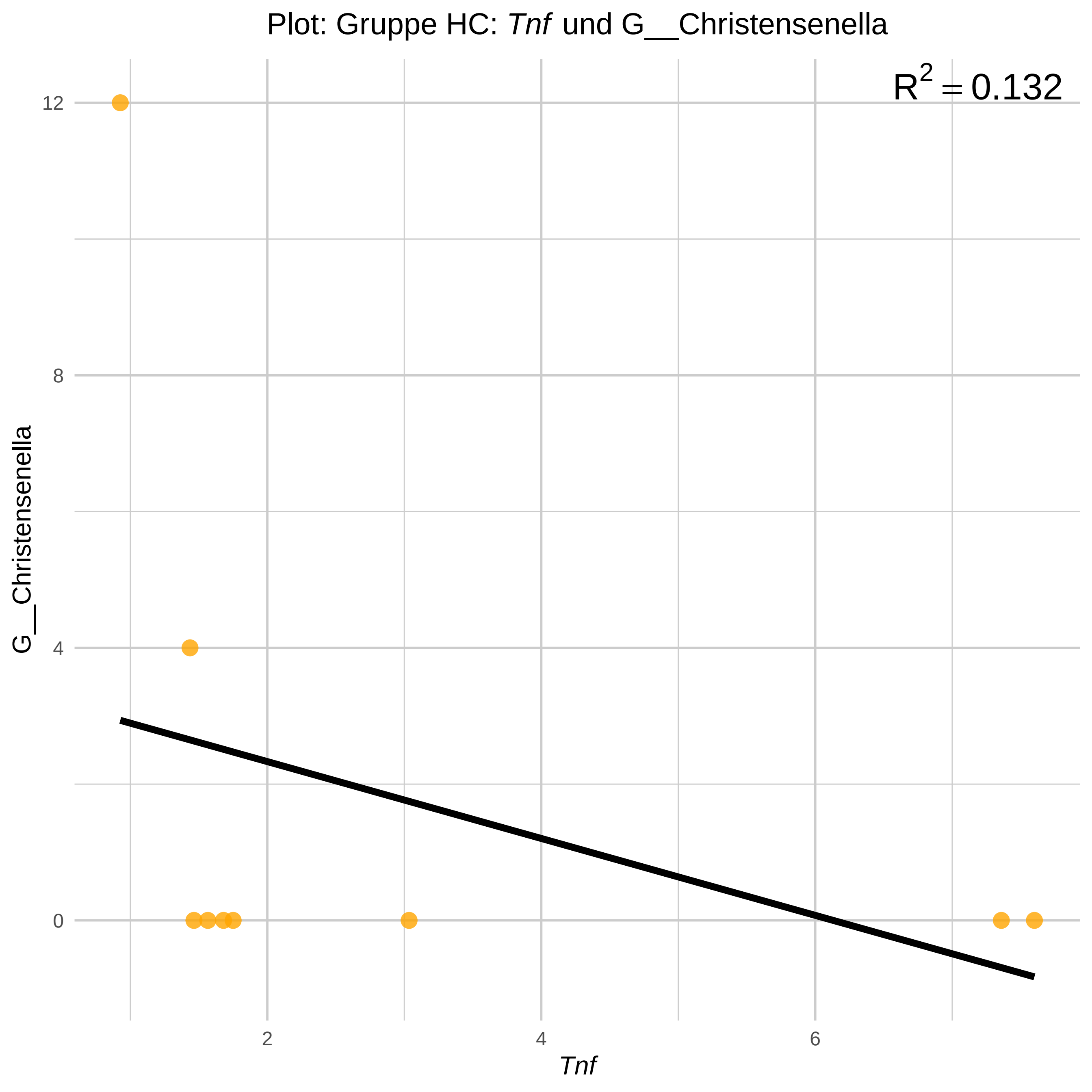

Plot: Gruppe HC: *Tnf* und G__Christensenella
$R^2 = 0.132$
12
8
4
0
G__Christensenella
2
4
6
Tnf

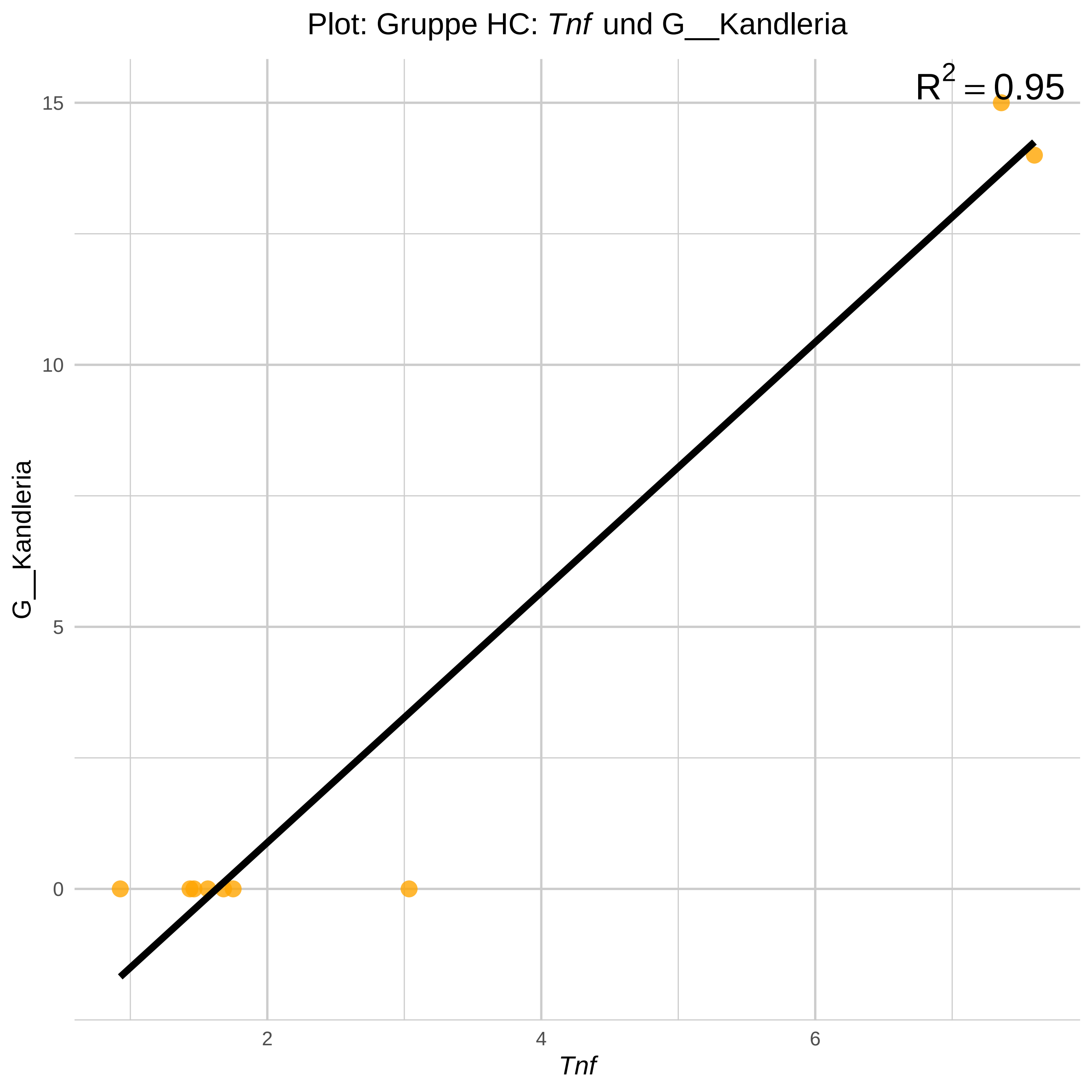

Plot: Gruppe HC: Tnf und G__Kandleria
R² = 0.95
G__Kandleria
Tnf
15
10
5
0
2
4
6

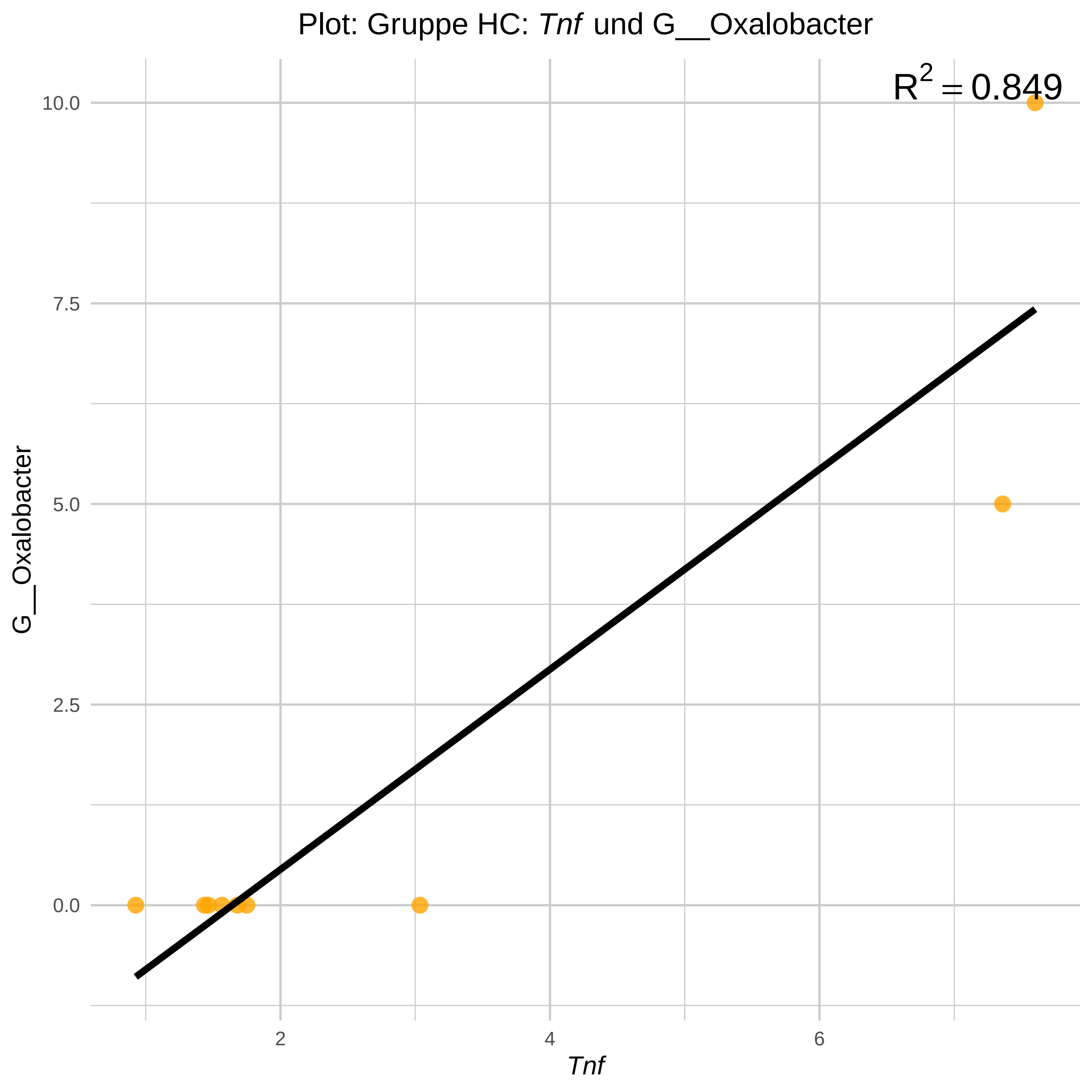
Plot: Gruppe HC: *Tnf* und G__Oxalobacter
$R^2 = 0.849$
10.0
7.5
5.0
2.5
0.0
G__Oxalobacter
2
4
6
Tnf
