# Supplementary material for: From gut to brain: effects of fecal microbiota transplants from humans to rats on hippocampal gene regulation - a study on anorexia nervosa
Source: Transl Psychiatry. 2026 Apr 30;16:238. doi: 10.1038/s41398-026-04056-9 (PMC13133121; doi:10.1038/s41398-026-04056-9)
Supplement: Supplementary file 4 — List of applied antibodies [file 41398_2026_4056_MOESM4_ESM.pdf]

Supplementary Tab.1: Information on applied antibodies.

**primary antibody**	**target**	**company**	**order number**	**host**	**clone**	**secondary antibody**	**antigen retrieval**	**concentration**
GFAP (glial fibrillary acidic protein)	astrocytes	Santa Cruz	sc33673	mouse	monoclonal	anti-mouse	Tris-EDTA, 10 min	1:2000
AIF1 (ionized calcium-binding-adapter molecule)	microglia	Wako	01919741	rabbit	polyclonal	anti-rabbit	Tris-EDTA, 10 min	1:10000
MAP2 (microtubule-associated protein 2)	neurons	Cell Signaling	D5G1	rabbit	polyclonal	anti-rabbit	Citrat, 20 min	1:1000
OLIG2 (oligodendrocyte-transcription-factor 2)	oligodendrocyte-lineage	Millipore	MABN50	mouse	monoclonal	anti-mouse	Tris-EDTA, 20 min	1:1000
