# Supplementary material for: From gut to brain: effects of fecal microbiota transplants from humans to rats on hippocampal gene regulation - a study on anorexia nervosa
Source: Transl Psychiatry. 2026 Apr 30;16:238. doi: 10.1038/s41398-026-04056-9 (PMC13133121; doi:10.1038/s41398-026-04056-9)
Supplement: Supplementary file 5 — List of applied primers [file 41398_2026_4056_MOESM5_ESM.pdf]

Supplementary Tab.2: Information on applied primers.

**gene of interest**	**target information (gene expression)**	**forward primer 5´-3´**	**reverse primer 5´-3´**	**annealing temperature**
Aif1 (ionized calcium-binding-adapter molecule)	quantification of microglial cell associated genes	TGGAGTTTGATCTGAATGGCAATG	AGCCACTGGACACCTCTCTA	65°C
B2m (beta-2-microglobulin)	reference gene, used for check PCR	ACGTGTCTCAGTTCCACCCACCT	TGTCTCGGTCCCAGGTGACGG	63°C
Bdnf (brain-derived neurotrophic factor)	neuroneogenesis	GCCACTGAAATGCGACTGAA	CACATCATTCCAGACCTGC	61°C
Cd11b (Itgam) (cluster of differentiation molecule 11b)	inflammation	GAGAACTGGTTCTGGCTTGC	TCAGTTCGAGCCTTCTT	60°C
Dcx (Doublecortin)	neuroneogenesis	AACGACCAAGACGCAAATGGA	GGGCTTGTGGGTGTAGAGAT	64°C
Gfap (glial fibrillary acidic protein)	quantification of astrocyte associated genes	AGAAAACCGCATCACCATTC	ACACCTCACATCACATCC	61°C
Il6 (Interleukin 6)	inflammation	GGTCTGTTGTGGGTGGTATCC	CCAGTTGCCTTCTTGGGACT	65°C
Map2 (microtubule associated protein 2)	neuroneogenesis	AAGCGGAAAACCACAGCAAC	GGTCTTGGGAGGGAAGAACG	58°C
Mki67 (Kiel-antigen 67)	proliferation	CTGCAGAGAAGGTTGGGATAAA	CTGACTTTGCCCAGAGATGAA	64°C
Olig1 (oligodendrocyte-transcription-factor 1)	quantification of oligodendrocyte associated genes	CGAGCGGAAGCGCATGCAGGA	AGAGCGAACTGGCGCACG	63°C
Ppia (Cyclophilin A)	reference gene	GGCAAATGCTGGACCAAACAC	TTAGAGTTGTCCACAGTCGGAGATG	65°C
Rbfox3 (neuron-specific nuclear protein)	neuroneogenesis	GTGCTGACCTCTATGGTGGA	TGTGTACACCCTGCCGTAAC	65°C
Tnf (Tumor necrosis factor alpha)	inflammation	TGCTTGGTGGTTTGCTACGA	TGATCGGTCCCACAAGGA	64°C
