# Supplementary material for: From gut to brain: effects of fecal microbiota transplants from humans to rats on hippocampal gene regulation - a study on anorexia nervosa
Source: Transl Psychiatry. 2026 Apr 30;16:238. doi: 10.1038/s41398-026-04056-9 (PMC13133121; doi:10.1038/s41398-026-04056-9)
Supplement: Supplementary file 6 — numbers of animal samples included per group in each analysis as n(analysis)/n(total) [file 41398_2026_4056_MOESM6_ESM.pdf]

Figure	Analysis	Control (n)	Vehicle (n)	FMT HC (n)	FMT AN (n)
2	physiological data (body weight, food intake, running wheel activity)	14/14	14/14	14/14	14/14
3	microbial analysis	T1: 14/14<br>T2: 14/14<br>T3: 14/14<br>T4: 10/14	T1: 14/14<br>T2: 14/14<br>T3: 13/14<br>T4: 12/14	T1: 14/14<br>T2: 13/14<br>T3: 14/14<br>T4: 11/14	T1: 14/14<br>T2: 14/14<br>T3: 14/14<br>T4: 11/14
4B	IHC GFAP	13/14	14/14	14/14	14/14
4C	IHC AIF1	14/14	14/14	13/14	14/14
4D	IHC OLIG2	14/14	13/14	13/14	13/14
4E	RT-qPCR *Gfap*	14/14	14/14	13/14	14/14
4F	RT-qPCR *Aif1*	14/14	14/14	12/14	14/14
4G	RT-qPCR *Olig1*	14/14	14/14	13/14	14/14
5A	RT-qPCR *Bdnf*	13/14	14/14	13/14	14/14
5B	IHC MAP	14/14	14/14	14/14	13/14
5C	RT-qPCR *Map*	12/14	14/14	12/14	13/14
5D	RT-qPCR *Rbfox3*	13/14	11/14	12/14	11/14
5E	RT-qPCR *Dcx*	12/14	14/14	12/14	13/14
5F	RT-qPCR *Mki67*	13/14	13/14	12/14	13/14
5G	RT-qPCR *Cd11b*	12/14	13/14	12/14	14/14
5H	RT-qPCR *Il6*	12/14	13/14	12/14	13/14
5I	RT-qPCR *Tnf*	12/12	12/14	11/14	12/14
