# Supplementary material for: From gut to brain: effects of fecal microbiota transplants from humans to rats on hippocampal gene regulation - a study on anorexia nervosa
Source: Transl Psychiatry. 2026 Apr 30;16:238. doi: 10.1038/s41398-026-04056-9 (PMC13133121; doi:10.1038/s41398-026-04056-9)
Supplement: Supplementary file 8 — Correlation of proliferatory markers and gene expression in the hippocampus of controls (C) and antibiotics treated group (V) [file 41398_2026_4056_MOESM8_ESM.pdf]

C	*Cd11b*	*Il6*	*Olig1*	*Tnf*
Bdnf		0.62	-0.77*	-0.8*
Mki67	0.68			

V	*Cd11b*	*Il6*	*Olig1*	*Tnf*
Bdnf	0.75*			0.59
Mki67	0.66*	0.73*		0.72*
