# Supplementary material for: From gut to brain: effects of fecal microbiota transplants from humans to rats on hippocampal gene regulation - a study on anorexia nervosa
Source: Transl Psychiatry. 2026 Apr 30;16:238. doi: 10.1038/s41398-026-04056-9 (PMC13133121; doi:10.1038/s41398-026-04056-9)
Supplement: Supplementary file 9 — Figure legends of Supplementary information [file 41398_2026_4056_MOESM9_ESM.pdf]

Supplementary Material

Supplementary File 1: ***16s rRNA sequencing.***

Protocol of 16s rRNA gene sequencing and processing.

Supplementary File 2: ***representative images of histolgical stainings (GFAP, AIF1, OLIG2, MAP).***

*Supplementary Table 1****: List of applied antibodies.***

*Supplementary Table 2****: List of applied primers.***

Supplementary Table 3: ***numbers of animal samples included per group in each analysis as n(analysis)/n(total).***

Supplementary Table 4a: ***Association of bacterial taxa abundance with hippocampal gene expression of FMT AN group (left) and FMT HC group (right).***

Supplementary Table 4b: ***Correlation of proliferatory markers and gene expression in the hippocampus of controls (C) and antibiotics treated group (V).***

Supplementary File 3: ***Correlation plots of association of bacterial taxa abundance with hippocampal gene expression of FMT AN group and FMT HC group.***
