# Supplementary material for: From gut to brain: effects of fecal microbiota transplants from humans to rats on hippocampal gene regulation - a study on anorexia nervosa
Source: Transl Psychiatry. 2026 Apr 30;16:238. doi: 10.1038/s41398-026-04056-9 (PMC13133121; doi:10.1038/s41398-026-04056-9)
Supplement: Supplementary file 7 — Association of bacterial taxa abundance with hippocampal gene expression of FMT AN group (left) and FMT HC group (right) [file 41398_2026_4056_MOESM7_ESM.pdf]

	hippocampal gene expression of FMT AN group											hippocampal gene expression of FMT HC group										
	Aif1	Bdnf	Cd11b	Dcx	Gfap	Il6	Map2	Mki67	Olig1	Rbfox3	Tnf	Aif1	Bdnf	Cd11b	Dcx	Gfap	Il6	Map2	Mki67	Olig1	Rbfox3	Tnf
G__Acetatifactor		0.59	0.66*						-0.63*				-0.62									-0.68*
G__Akkermansia		-0.81**	-0.72*			-0.75*			0.58													
G__Acetobacterium														0.62			0.59	-0.73*	0.59			
G__Aestuariispira																	0.63*			0.65*		
G__Alistipes				0.65*									-0.64	-0.81**				0.68			0.82*	
G_Alloprevotella		-0.53																				
G__Anaerotruncus																0.66*						
G__Anaerofustis			0.58			0.80**			-0.58		0.64											
G_Anaerovorax	0.58																					
G__Asaccharobacter						0.70*					0.72*											
G_Bacteroides													-0.6									
G_Barnesiella								0.61		-0.66					0.67							
G_Bilophila				0.55																		
G_Blautia	0.68*				0.62*		0.80**											0.76*				
G__Butyricicoccus	-0.61		-0.54								-0.83**											
G__Butyricimonas						0.82**					0.68*											
G__Christensenella														-0.70*								-0.73*
G__Clostridium_IV																0.65*						
G__Clostridium_sensu_stricto					0.67*							-0.73*										
G__Clostridium_XlVa														-0.61								
G__Clostridium_XlVb																0.64*						
G__Clostridium_XVIII																		0.72*				
G__Collinsella	0.54	0.54					0.57								0.73*							
G__Coprobacter				0.54																0.64*		
G__Coprococcus																				-0.70*		
G__Defluviitalea																	0.62	-0.82**	0.56	0.62		
G__Desulfovibrio		-0.57				-0.87***					-0.73*											
G__Desulfobaculum														0.59			0.62	-0.71*	0.62			
G__Eisenbergiella				0.66*								0.67*										
G__Enterobacter																		0.59		-0.81**		
G__Enterococcus	0.59															-0.70*						
G__Enterorhabdus				-0.57	0.63*															-0.73*		
G__Escherichia.Shigella																	0.58	-0.88**		0.81**		
G_Eubacterium													-0.61	-0.61		0.58						
G__Faecalibacterium															-0.59					-0.68*		
G__Flavonifractor		-0.64*						-0.56						-0.72*							0.63	-0.65
G__Holdemania				0.5	0.86***						-0.61		-0.62	-0.71*		0.67 *						
G__Hydrogenoanaerobacterium				0.6							0.59				-0.82**							
G__Intestinimonas															-0.59	0.65*					0.67*	
G__Kandleria												0.62					0.68*					0.71*
G__Lactobacillus																				-0.65*		
G_Marvinbryantia																					0.6	
G__Olsenella		-0.54	-0.66*			-0.70*		-0.70*			-0.71*											
G__Odoribacter													-0.72*									
G__Oscillibacter					-0.63*										-0.72*							
G__Oxalobacter										-0.73*		0.59					0.70*					0.73*
G_Parasporobacterium																		0.59				
G__Parasutterella															-0.82*							
G__Peptococcus											-0.64											
G__Prevotella		-0.67*	-0.62*			-0.76*		-0.67*			-0.83**			-0.65*		0.58		0.72*			0.63	
G__Proteus						0.68*					0.71*											
G__Pseudoflavonifractor															-0.73*						0.82*	
G__Romboutsia												-0.62					-0.68*					
G__Ruminococcus													-0.84**									
G__Ruminococcus2																	-0.75*		-0.59			-0.63
G__Sporobacter				0.76**											-0.78*							
G__Sutterella		-0.54				-0.55									0.71*							
G__Turicibacter				-0.53	0.74*							-0.61					-0.56			-0.65*		
